# Unhealthy Food and Beverage Consumption in Children and Risk of Overweight and Obesity: A Systematic Review and Meta-Analysis

**DOI:** 10.1093/advances/nmac032

**Published:** 2022-04-01

**Authors:** E K Rousham, S Goudet, O Markey, P Griffiths, B Boxer, C Carroll, E S Petherick, R Pradeilles

**Affiliations:** Centre for Global Health and Human Development, School of Sport, Exercise and Health Sciences, Loughborough University, Loughborough, United Kingdom; Centre for Global Health and Human Development, School of Sport, Exercise and Health Sciences, Loughborough University, Loughborough, United Kingdom; Centre for Global Health and Human Development, School of Sport, Exercise and Health Sciences, Loughborough University, Loughborough, United Kingdom; Centre for Global Health and Human Development, School of Sport, Exercise and Health Sciences, Loughborough University, Loughborough, United Kingdom; Centre for Global Health and Human Development, School of Sport, Exercise and Health Sciences, Loughborough University, Loughborough, United Kingdom; School of Health and Related Research, The University of Sheffield, Sheffield, United Kingdom; Centre for Global Health and Human Development, School of Sport, Exercise and Health Sciences, Loughborough University, Loughborough, United Kingdom; National Institute for Health Research (NIHR) Leicester Biomedical Research Centre, University Hospitals of Leicester NHS Trust and University of Leicester, Leicester, United Kingdom; Centre for Global Health and Human Development, School of Sport, Exercise and Health Sciences, Loughborough University, Loughborough, United Kingdom

**Keywords:** complementary food, infant and young child nutrition, diet, ultraprocessed foods, sugar-sweetened beverages, infant, child, low-and middle-income countries, cohort, GRADE approach

## Abstract

This WHO-commissioned review contributed to the update of complementary feeding recommendations, synthesizing evidence on effects of unhealthy food and beverage consumption in children on overweight and obesity. We searched PubMed (Medline), Cochrane CENTRAL, and Embase for articles, irrespective of language or geography. Inclusion criteria were: *1*) randomized controlled trials (RCTs), non-RCTs, cohort studies, and pre/post studies with control; *2*) participants aged ≤10.9 y at exposure; *3*) studies reporting greater consumption of unhealthy foods/beverages compared with no or low consumption; *4*) studies assessing anthropometric and/or body composition; and *5*) publication date ≥1971. Unhealthy foods and beverages were defined using nutrient- and food-based approaches. Risk of bias was assessed using the ROBINS-I (risk of bias in nonrandomized studies of interventions version I) and RoB2 [Cochrane RoB (version 2)] tools for nonrandomized and randomized studies, respectively. Narrative synthesis was complemented by meta-analyses where appropriate. Certainty of evidence was assessed using Grading of Recommendations Assessment, Development, and Evaluation. Of 26,542 identified citations, 60 studies from 71 articles were included. Most studies were observational (59/60), and no included studies were from low-income countries. The evidence base was low quality, as assessed by ROBINS-I and RoB2 tools. Evidence synthesis was limited by the different interventions and comparators across studies. Evidence indicated that consumption of sugar-sweetened beverages (SSBs) and unhealthy foods in childhood may increase BMI/BMI *z*-score, percentage body fat, or odds of overweight/obesity (low certainty of evidence). Artificially sweetened beverages and 100% fruit juice consumption make little/no difference to BMI, percentage body fat, or overweight/obesity outcomes (low certainty of evidence). Meta-analyses of a subset of studies indicated a positive association between SSB intake and percentage body fat, but no association with change in BMI and BMI *z*-score. High-quality epidemiological studies that are designed to assess the effects of unhealthy food consumption during childhood on risk of overweight/obesity are needed to contribute to a more robust evidence base upon which to design policy recommendations.

This protocol was registered at https://www.crd.york.ac.uk/PROSPERO as CRD42020218109.

## Teaser

**Statement of Significance**: This systematic review was the first to synthesize evidence on the effects of consuming unhealthy foods and beverages in children aged ≤10.9 y across low-, middle-, and high-income country settings. This review identified a major evidence gap from low- and middle- income countries. Consumption of sugar-sweetened beverages and unhealthy foods in childhood may increase BMI, percentage body fat, or odds of overweight/obesity, whereas consumption of artificially sweetened beverages and 100% fruit juice has less demonstrable effects on these outcomes.

## Introduction

Infants and children are consuming increasing amounts of foods with added sugars, high in salt, and high in saturated or *trans* fats (1, 2). Commercially prepared foods are more likely to be high in energy, low in nutrients (energy-dense, nutrient-poor), and ultraprocessed (3, 4). Globally, the consumption of sugary and savory snacks and refined foods has been increasing across all socioeconomic groups (5). These foods can have direct consequences on health, as well as indirect consequences through displacement of healthy foods in the diet (6). Consumption of foods that are energy-dense and nutrient-poor is a particular risk for malnutrition in socioeconomically disadvantaged groups and urban communities in low- and middle-income countries (LMIC) leading to increasing disparities in health globally (7). Exposures to sugar-sweetened beverages (SSBs) and sweet foods in childhood can contribute to sweet food preferences in later life (7). Diet quality in early life is also important for child development (6), and suboptimal diet is a preventable risk factor for noncommunicable diseases (NCDs) (8).

Existing complementary feeding guidelines were developed when the prevention of undernutrition was a primary concern (9, 10). In response to increasing rates of childhood overweight/obesity and the rising prevalence of NCDs (6), however, complementary feeding guidelines need to consider all forms of malnutrition including undernutrition, micronutrient deficiencies, and overweight or obesity (5, 11, 12).

A previous systematic review examined the impact of consuming unhealthy foods and beverages in the complementary feeding period (age 6–23 mo) in countries ranked high or very high on the Human Development Index (13). Limited evidence suggested that SSB consumption was associated with greater obesity risk in children aged <2 y, but not other growth or body composition indicators (13). A systematic review of 32 studies in high-income countries (HIC) concluded that SSB consumption promotes weight gain in children, adolescents, and adults (14). A systematic review of 100% fruit juice consumption in longitudinal studies reported non–clinically significant BMI *z*-score increases and a lack of evidence for children under 7 y (15). Positive associations were reported between ultraprocessed food (UPF) consumption and percentage body fat in children and adolescents in a systematic review including both cross-sectional and longitudinal study designs (16). Findings from cross-sectional studies provide weak evidence of associations because of the potential for reverse causality (17).

Existing studies and reviews highlight the paucity of evidence on effects of unhealthy food consumption in the complementary feeding period (1, 13). There has also been very little consideration of these effects in LMIC settings. A review of cross-sectional and longitudinal studies in LMIC found limited and inconclusive evidence on the relation between snack food, SSB consumption, and child growth and dietary adequacy in children aged 6–23 mo (7), meaning a review of all country settings is needed.

The WHO, as part of the process of updating the guiding principles for complementary feeding (9, 10), commissioned a series of systematic review reports, one of which examined the impact of unhealthy food and beverage consumption on prespecified critical (growth, overweight/obesity and body composition; diet-related NCD indicators (cardiometabolic disease risk outcomes); displacement of healthy foods or breastmilk intake; dietary quality and diversity) and important (food or taste preferences later in life; oral health/dental caries; micronutrient deficiencies; and child development) outcomes. There is limited evidence on the effects of unhealthy food and beverage consumption in infants and young children. Furthermore, the entire childhood period is a critical window for reducing malnutrition and obesity-related NCD risk in later life (18). Hence, the aim of the current systematic review was to examine, in children aged ≤10.9 y, the risks of greater consumption of unhealthy foods and beverages compared with no or low consumption on overweight and obesity.

## Methods

### Review typology

A systematic review and meta-analysis were chosen to systematically search for, appraise, and synthesize quantitative research evidence (19). We followed the Preferred Reporting Items for Systematic Reviews and Meta-Analyses 2020 reporting guidelines (20). The study protocol for the review was registered on the International Prospective Register of Systematic Reviews, PROSPERO (https://www.crd.york.ac.uk/prospero/; registration number: CRD42020218109).

### Eligibility criteria

Study eligibility and inclusion criteria are shown in **Table 1** for population/participants, interventions or exposures, comparators, and outcomes (PI/ECO), and study design. We included quantitative human studies of children where age at intervention or exposure was between birth and ≤10.9 y, published from January 1971 with no restriction on publication language. Non–English language records were screened by native speakers with subject-specific knowledge.

**TABLE 1:** Inclusion and exclusion criteria for the review of the effect of unhealthy food and beverage consumption in children aged ≤10.9 y and risk of overweight and obesity^1^

Parameter	Inclusion criteria	Exclusion criteria
Participants/population	Human studies including both boys and girls	Nonhuman studies
	Age at intervention or exposure: infants from birth to ≤10.9 y	Age at intervention or exposure >10.9 y
		Studies that exclusively enroll participants with a disease or with the health outcomes of interest (listed below)
		Studies using hospitalized patients; severely malnourished participants, or clinical populations
		Studies of exclusively preterm babies (<37 wk gestation) or exclusively babies that are low birth weight (<2500 g) or small-for-gestational age
Independent variable (intervention or exposure)	Studies reporting (greater) consumption of unhealthy foods and beverages compared with no or low consumption	Studies not reporting consumption of unhealthy foods and beverages as per the protocol definition of consumption
	Unhealthy foods defined using: *1*) nutrient-based approaches [foods high in added sugars, free sugars, artificial sweeteners, fats (e.g., saturated/*trans*), salt]; and food-based approaches including: *2*) ultraprocessed foods (based on NOVA classification, excluding formula and follow-on milks); *3*) unhealthy foods and beverages listed in the WHO/UNICEF infant and young child feeding guide (21); *4*) food items defined by authors using terms such as “fast-food,” “convenience foods,” “non-core foods”	Studies reporting only dietary patterns (i.e., data reduction techniques such as principal component analysis) or eating practices (e.g., meals per day; snacking patterns; meal times and duration of eating episodes)
	Consumption defined as: *1*) quantities consumed (grams per day, week, or month); *2*) portion sizes; *3*) frequency of consumption (per week, month, year), or consumed/not consumed	
Comparator	Consumption of less or no unhealthy foods and beverages: no or low added sugar, free sugars, artificial sweeteners; less fat (or less of certain types of fat), less consumption of foods high in salt or ultraprocessed/energy-dense, nutrient-poor foods	
Study design	Randomized controlled trials	Cross-sectional studies
	Nonrandomized controlled trials (including historically controlled studies)	Trials without a control group
	Prospective cohort studies (including interrupted time series analyses)	Narrative reviews, systematic reviews and meta-analyses
	Retrospective cohort studies	Case-control studies: i.e., cases with disease (e.g., diabetes) vs. controls without disease
	Pre/post studies with a control	Pre/post studies without a control
Dependent variable (outcome)	Growth and body composition: stunting; length-for-age or height-for-age; underweight or weight-for-age; wasting or weight-for-length/weight-for-height; BMI; BMI *z*-score; waist circumference; prevalence of overweight or obesity; percentage body fat	
Country	All contexts (high-, middle-, and low-income countries)	NA
Date range	Articles published from 1971 onwards	Articles published before 1971
Publication status	Reports published in peer-reviewed journals	Conference abstracts, conference proceedings, unpublished data, reports, letters, editorials
Language	All languages	NA

1 NA, not applicable.

Unhealthy foods and beverages were defined using both nutrient- and food-based approaches. Four main measures were used to classify foods and beverages as unhealthy, including: *1*) UPFs based on the NOVA classification (22); *2*) unhealthy foods and beverages according to WHO infant and young child feeding indicators (21); *3*) foods high in free sugars, artificial sweeteners, and salt; and *4*) foods high in saturated or *trans* fats. In addition, we included studies in which authors defined unhealthy foods using terminologies such as: “junk food,” “fast food,” “snack food,” “extra food,” “non-core food,” and “convenience food,” which also met inclusion criteria based on consumption of foods high in sugar, salt, fat, or UPFs. Studies reporting dietary patterns were not included because these only provide evidence on overall combinations of foods such as “unhealthy” diets compared with “healthier” diets. Eligible studies were subsequently grouped into exposures of unhealthy beverages [SSBs; artificially sweetened beverages (ASBs), and 100% fruit juice separately] and unhealthy foods. For detailed definitions of unhealthy foods and beverages see **Supplemental Method 1** and **Supplemental Table 1**.

### Search strategy

A literature search strategy was developed and checked by an academic librarian. Scoping searches were conducted to refine the search strategy ensuring that known relevant studies had been identified. Systematic searches were run in PubMed (MEDLINE), Cochrane CENTRAL, and Embase during December 17–23, 2020. Gray literature was not included in the systematic search due to time and budgetary constraints.

The search results were imported into Covidence software (Veritas Health Innovation), which was employed for screening titles, abstracts, and full texts. Citation alerts were set up in PubMed to flag new potentially relevant items. Additional supplementary searches included reference checking of included publications and relevant systematic reviews and consultation with subject experts for relevant published studies. Supplementary searches continued until April 30, 2021. The search strategies for each database are included in **Supplemental Table 2**.

### Study selection

Duplicate records were identified automatically by the review software prior to screening. Half of the duplicate records were checked and no incorrect duplicates were identified.

All reviewers underwent training by screening the same test sample of 25 records selected at random. The results of the test screening were combined and discussed across the review team; this informed further guidance on inclusion and exclusion criteria. We amended the protocol with a change in the age inclusion criteria from <10 y to ≤10.9 y to ensure consistency in screening and greater inclusion of evidence.

All records included at title/abstract and full-text stage were screened by 2 reviewers independently (OM, RP, SG, PG, EKR, Natalie Pearson, Kathrin Burdenski, or Megan Stanley). Conflicting votes were considered by a third reviewer (RP or NP) and a fourth reviewer in cases of uncertainty (EKR). Two reviewers each checked 2 distinct random 10% samples of excluded records at title/abstract and full-text stage (OM, RP, SG, or EKR). Reasons for exclusions at full-text screening were recorded. Studies that met all inclusion criteria but reported data for a wider age range (e.g., 8–13 y) were included. The review team e-mailed study investigators (with a follow-up e-mail to nonresponders) to request disaggregated data for participants aged ≤10.9 y. Of the 16 study authors contacted, 8 responded to say that the datafile was no longer available. One study author provided disaggregated data (23).

### Data collection process, data items, and effect measures

An Excel data extraction form was developed and piloted by all data extractors using a selection of 6 included articles covering different review outcomes. This resulted in modifications, and a second pilot data extraction was undertaken with all reviewers extracting data from a single article and comparing notes. Further modifications were made to finalize the data extraction form. One reviewer undertook data extraction independently (OM, RP, SG, BB, or EKR). Any data extraction queries were discussed among the team. A second reviewer (EKR) checked 50% of all records extracted for completeness and accuracy.

Full details of the information extracted from eligible studies are presented in **Supplemental Table 3**. Data extracted from studies included: *1*) general information [study ID, title, authors, start and end date, study location (country, urban compared with rural), study design, study aim, aim of intervention, study funding sources, conflicts of interest, ethical approval reported]; *2*) study eligibility (participant selection and randomization process), sample size, participant characteristics (age, number, and sex), duration of intervention, exposure measures (type of food consumption data, unit of measurement, and dietary assessment methods) and critical and important review outcomes, and the method of assessment of outcomes; and *3*) study findings.

We extracted data on all ages of follow-up to assess longer-term outcomes. We recorded the variables adjusted for in analyses, such as education, socioeconomic status, sex, maternal age, race and/or ethnicity, other feeding practices (breast milk, infant formula, or both), and birth weight/length. Study protocols and supplementary materials were searched for data extraction if the required data were not presented in the included articles. For studies not in English, data extraction and risk of bias (RoB) were conducted with 1 review team member working alongside a researcher proficient in the native language with relevant subject expertise.

We extracted the measures of intervention/exposure effect (mean differences, ORs, β coefficients, RRs with 95% CIs, and/or *P* value) for the outcome of interest from all studies. We extracted data from fully adjusted models where available. If unadjusted effect measures only were reported, these were extracted. Where multiple articles from the same study were included, we extracted data that were unique to each article. In some instances, this resulted in a greater number of articles than studies (i.e., 2 articles from the same study were included if different outcomes or exposures were reported). If the same data were reported in >1 included article, we extracted data from the article that most closely addressed the review question.

### RoB assessment

RoB was assessed by 2 reviewers (OM, RP, SG, BB, or EKR) independently using Covidence to ensure blinding. Reviewers noted justifications for any domains that were assessed as serious, critical, or no information. Information was checked from study protocols, clinical trial registers, and supplementary files if not presented in the included reports. Reviewers discussed discrepancies and reached consensus on each domain of the RoB tool. If agreement could not be reached, a third reviewer (RP or EKR) assessed RoB and a consensus was reached. RoB was conducted at the outcome level.

#### RoB for nonrandomized studies of the effects of interventions (prospective cohort studies)

The ROBINS-I tool (risk of bias in nonrandomized studies of interventions version I) was applied in accordance with Cochrane and Grading of Recommendations Assessment, Development, and Evaluation (GRADE) considerations of observational studies as nonrandomized studies of interventions (NRSIs) (24, 25). Each of the 7 domains of the ROBINS-I tool were rated as being at low, moderate, serious, or critical RoB, or no information (26). After completing consensus on the 7 domains, the overall RoB for each study was assessed using the criteria in **Supplemental Table 4**.

#### RoB for RCTs

Cochrane RoB (Version 2: RoB 2.0) was used for RCTs (25, 27). RoB 2.0 addresses 5 domains. Each domain was rated as low, some concerns, high RoB, or no information. Supporting information and justifications for judgments in each domain were recorded. After reaching consensus on the 5 domains, the overall RoB was assessed using Cochrane guidance, as presented in **Supplemental Table 5** (25).

Some studies undertook a secondary analysis of data from a previous RCT to address a research question unrelated to the original trial (28–33). The trials had either reported no significant effects of the intervention and therefore pooled the intervention and control group, or assessed the control group only. We assessed these studies as NRSIs and applied the ROBINS-I tool. Individual and summary RoB tables were produced using the “robvis” tool (34).

### Data synthesis

#### Initial data synthesis processes

We synthesized findings using the PI/ECO framework, first grouping studies by outcome and then by intervention/exposure. For synthesis relating to participant characteristics, we stratified by age (0 to <2 y; 2 to <5 y, and 5 to ≤10.9 y) where there were sufficient studies. For completeness, we included all estimates in summary tables of results, including studies with critical RoB. In narrative synthesis, meta-analysis, and when grading the evidence, however, we did not include results from studies assessed as critical RoB, in line with guidance (24, 35).

For the synthesis of growth, body composition, and overweight/obesity outcomes, we prioritized studies that reported BMI, BMI *z*-score, BMI change, BMI *z*-score change (or for children <2 y, weight-for-length), or prevalence of overweight/obesity because these are the most widely used indicators of growth and overweight or obesity. We then collated studies with effect estimates for percentage body fat because this was a relatively homogeneous outcome across studies. For completeness, data for other indicators such as waist circumference, central adiposity, waist-to-height ratio, and sums of skinfold thicknesses were extracted and included in summary tables, but not described in detail.

Exposures were synthesized using 2 overarching groups: *1*) unhealthy beverages—this was disaggregated into SSBs alone, ASBs only, and 100% fruit juice only, where studies specifically reported these items separately (any fruit drink that was not 100% fruit juice was included within the SSB category); and *2*) unhealthy foods.

#### Data synthesis methods

Heterogeneity across studies arose primarily from measurement of exposure (including the dietary assessment methods, recall period, definition of food items/food groups, or units of measurement). Data reporting varied from dichotomous, multiple categories or continuous measures of consumption. Where exposures could be harmonized for the same study outcomes, we included in meta-analyses. A priori, we set a minimum requirement of 2 studies reporting the same outcomes and the same study design to produce a forest plot.

For SSB and 100% juice consumption, there were studies that could be harmonized based on the reported quantities of consumption, or number of servings, in relation to the reported outcomes, hence these were pooled for meta-analyses and corresponding forest plots. Further information on the harmonization process can be found in **Supplemental Method 2**. *I*^2^ values were generated as indicators of heterogeneity, although these should be interpreted with caution when there are few studies in a meta-analysis. We adopted interpretative guidance for heterogeneity of 0% to 40% as not important; 30% to 60% moderate; 50% to 90% substantial, and 75% to 100% considerable heterogeneity (25). These ranges overlap because these are not absolute cut-offs. Reported β coefficients and SEs were either multiplied or divided to achieve the common serving size estimate. Random effects models were performed as recommended where heterogeneity is likely. Analyses were undertaken using the meta command in Stata SE 16 (StataCorp).

For unhealthy food consumption, we examined all studies to identify those that could be harmonized. Data on exposures and comparators for unhealthy food consumption could not be harmonized from ≥2 studies (see **Supplemental Method 3** for further details). We therefore performed narrative synthesis. We followed synthesis without meta-analysis guidelines for data synthesis without meta-analysis (35).

### Reporting bias assessment

Funnel plots were not undertaken to examine potential publication bias in the meta-analysis given we could not meet the recommended number of ≥10 studies included in meta-analyses (25). Bias due to missing participants was considered within the RoB assessment using ROBINS-I for NRSIs.

### Certainty of evidence

We used GRADE criteria to assess the certainty of evidence for the effect of exposures on the critical outcomes. Two reviewers (SG, EKR) independently graded the evidence as high, moderate, low, and very low, then agreed ratings through discussion and consensus. Statements defining the certainty for each grade are provided in **Supplemental Table 6**. Detailed information on the grading of the evidence can be found in **Supplemental Method 4**. Evidence profile tables were produced for each outcome using GRADEpro GDT software (GRADEpro Guideline Development Tool) following recommended guidance (36) and disaggregated by age (<2 y; 2 to <5 y; 5 to ≤10.9 y) where there were sufficient numbers of studies. Studies of critical RoB were excluded from GRADE evidence profiles in line with guidance (25). We used standard statements to report the results of the review interventions (exposures) based on guidelines (37).

## Results

### Study selection

**FIGURE 1:**
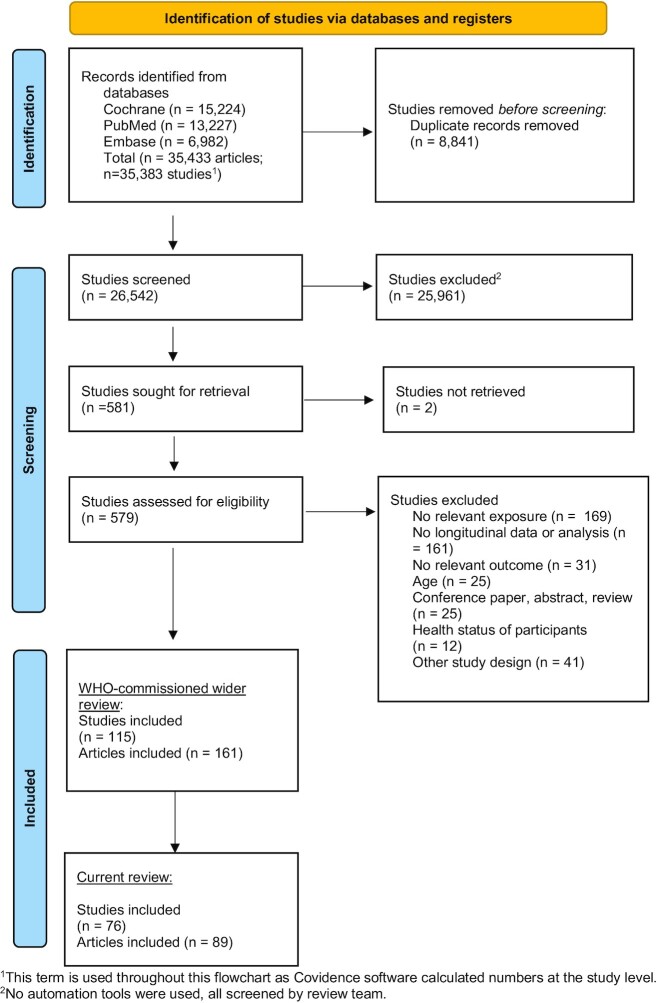
Flow chart of study search and selection for the review of effects of unhealthy food or beverage consumption in children aged <10.9 y on risk of overweight/obesity.

The wider search for the WHO review retrieved 35,433 studies with 8841 duplicate records detected. **Figure 1** presents the study search and selection process (20). We screened 26,542 studies of which 581 were eligible for full-text review. Of these, 579 studies were assessed for eligibility because 2 studies could not be retrieved (38, 39). After full-text screening, 161 articles from 115 studies were included. Of the included studies, 89 articles from 76 studies focused on growth and body composition outcomes. Data could not be extracted from 13 studies because they included participants younger and older than 10.9 y and results were not age-stratified (40–52). There were 3 further articles from 2 studies where data could not be extracted because of age range but data were extracted from other articles from the respective studies (53–55). Characteristics of the studies and articles where data could not be extracted are presented in **Supplemental Table 7**.

### Study and participant characteristics

We summarized characteristics of 71 articles from 60 included studies. Some studies had >1 included article because different outcomes or exposures were reported in each article. The extracted data are summarized in **Supplemental Table 8**, with the country, setting, study design, baseline age, exposure details, and outcomes assessed. Studies were published from 1993 to 2020. Around 88.5% (53/60) of studies were conducted in HIC and 11.5% (7/60) in middle-income countries (MIC); no studies were from low-income countries, based on the current gross national income per capita (56). Studies in MIC were conducted in Belarus, Brazil, China, Mexico, Peru, and South Africa. One study was a pre/post design with a control (57), 1 was a retrospective cohort design (58), and 1 was an RCT (59). The remaining studies (*n* = 57) used prospective cohort designs. About 43.5% of studies included participants from urban settings; 13.3% from both rural and urban areas; and only 8.3% from rural areas. Twenty-one studies (35.0%) did not report the residence/location of participants. Sample size at baseline ranged from 72 (60) to 16,058 (61). Baseline participant age ranged from 1 mo to 10.8 y. Two studies recruited only girls as participants (62, 63). The oldest ages at follow-up were 20–21 y (63) and 21 y (64).

### RoB assessment

**FIGURE 2:**
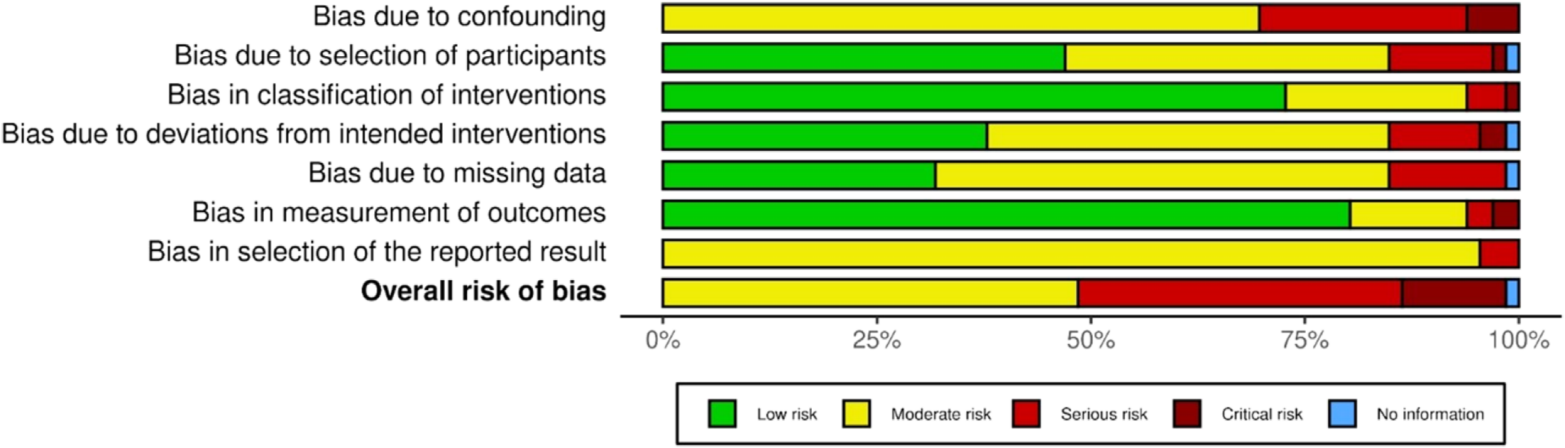
Summary risk of bias assessment of nonrandomized studies reporting unhealthy food or beverage consumption and growth, body composition, and overweight/obesity outcomes assessed using ROBINS-I (risk of bias in nonrandomized studies of interventions version I) tool.

Sixty-seven articles from 60 studies reported on growth and body composition. One study was an RCT (59), and 59 were observational studies (NRSIs). Sixty-six articles from 59 observational studies (NRSIs) were assessed for RoB. Four articles were not assessed for RoB because they did not provide any additional effect estimates to the selected article from the same study (65–68). No articles had low RoB; 32 articles (48.5%) had moderate RoB (29, 57, 58, 64, 69–96), 25 articles (37.9%) had serious RoB (60, 62, 97–119), and 8 articles (12.1%) had critical RoB (61, 120–126) (**Supplemental Figure 1**). One article (1.6%) (127) was classed as having “no information.” The ROBINS-I domains that most contributed to moderate, serious, or critical RoB were confounding bias (D1), participant selection bias (D2), bias due to intervention protocol deviations (D4), and missing data bias (D5) (**Figure 2**). The 1 included RCT study (59) was assessed as “some concerns” (**Supplemental Figure 2**).

### Synthesis

#### SSBs and growth, body composition, and overweight/obesity outcomes

Forty-five studies reported on SSB consumption and growth, body composition, and overweight/obesity outcomes. Some studies analyzed sodas, juice drinks, or other sweetened beverages separately (57, 63, 83, 122) whereas other studies examined multiple types of SSB as a single category. Studies were predominantly from HIC. Studies from MIC were conducted in China, Brazil, Peru, Mexico, and Belarus (70, 77, 91, 110, 115).

##### SSB consumption and BMI and overweight/obesity outcomes (narrative synthesis)

Thirty-five of the 45 studies reported BMI outcomes (raw values, percentiles or *z*-scores, or change in raw/*z*-score values) and/or overweight/obesity prevalence (**Supplemental Table 9**).

Children aged <2 y were examined in 10 studies, 2 of which had critical RoB and are not reported on further (61, 123). Of the remaining 8 studies, 3 reported a positive association. Consumption of SSBs in early life was associated with significantly higher odds of obesity at ages 8–14 y [adjusted odds ratio (aOR) = 2.99; 95% CI: 1.27, 7.00] (serious RoB) (115). Similarly, SSB consumption >1/wk compared with ≤1/wk in infancy was associated with greater odds of overweight and obesity at age 17 mo (aOR = 1.6; 95% CI: 1.04, 1.93; *P* < 0.01) (serious RoB) (110). Pan et al. (104) reported that SSB intake at 10–12 mo was associated with significantly greater odds of obesity in the highest intake group (≥3 times/wk) compared with no consumption, but not in the intermediate intake groups (<1 or 1 to <3 times/wk) compared with no consumption (serious RoB). The same study compared “any” with “no” consumption of SSB from 1 to 12 mo and observed a higher prevalence of obesity at 6 y in the group that consumed SSBs (aOR = 1.71; 95% CI: 1.09, 2.68) (104). Three studies reported different effects based on either the time point of assessment, differences between boys or girls, or the outcome assessed. Flores and Lin (97) reported no association between SSB consumption at age 2 y and severe obesity at 5 y; only SSB consumption at 5 y was associated with severe obesity at 5 y (aOR = 2.3; 95% CI: 1.4, 3.7) (serious RoB). Quah et al. (105) reported that SSB intake at 18 mo was not associated with BMI *z*-score or overweight/obesity at 6 y, but intake at 5 y was significantly associated with both outcomes (β = 0.34; 95% CI: 0.11, 0.58; *P* = 0.004; RR = 1.54; 95% CI: 1.03, 2.30; *P* = 0.033, respectively) (serious RoB). Leermakers et al. (128) found a significant association between SSB intake and BMI *z*-score in girls, but not in boys (girls: β = 0.11; 95% CI: 0.00, 0.23; *P* = 0.04; boys: β = 0.05; 95% CI: −0.08, 0.18; *P* = 0.42) at 6 y (moderate RoB). Two studies reported no association between consumption of SSBs and BMI or overweight outcomes (both serious RoB) (111, 112).

In children aged 2 to <5 y, 11 studies reported on SSB intake and BMI or overweight/obesity **(Supplemental Table 9**). In 1 study, results were not extractable (117). Four of the remaining 10 studies reported an association. In US children, SSB intakes in children aged 2–4.7 y at baseline and followed up at age 12.3–15 y were significantly positively associated with BMI *z*-score (β = 0.05; 95% CI: 0.022, 0.079; *P* = 0.001) (moderate RoB) (81). In Australian children, SSB intake per day was significantly positively associated with BMI *z*-score (β = 0.017; 95% CI: 0.007, 0.027; *P* < 0.01) (moderate RoB) (73). Consuming SSBs above compared with below the median intake (>65 mL/d compared with <65 mL/d) at 18 and 30 mo was associated with increased odds of overweight/obesity at 18-mo follow-up (aOR = 1.92; 95% CI: 1.19, 3.11; *P* ≤ 0.01) and at 30-mo follow-up (aOR = 1.82; 95% CI: 1.11, 3.00; *P* ≤ 0.05) (serious RoB) (118). In 1 study, total daily consumption of SSBs was not associated with obesity prevalence, but regular consumers of SSBs between meals compared with those who did not consume between meals at 2.5 y had greater odds of obesity at 4.5 y (aOR = 2.36; 95% CI: 1.03, 5.39; *P* ≤ 0.05) (moderate RoB) (96). Five studies (6 articles) reported no association (all moderate RoB) (58, 70, 72, 74, 83, 93). One study reported no association between SSB consumption and odds of overweight/obesity, but significantly greater odds for obesity alone (obese: OR = 1.65; 95% CI: 1.12, 2.44; *P* = 0.01) (moderate RoB) (80).

In children aged 5 to ≤10.9 y, 16 studies reported on SSB and BMI or overweight/obesity, 1 study did not report effect estimates (107), and 2 had critical RoB (120, 121) (**Supplemental Table 9**). Of the 13 studies with included effect estimates, all were observational designs except 1 RCT (59). In a cluster RCT in Germany, SSB intake in children was associated with significantly greater odds of obesity (aOR = 1.22; 95% CI: 1.04, 1.44; *P* = 0.014) but not overweight. There was also an association with SSB intake (per 200-mL glass/d) and BMI change (β = 0.02; 95% CI: 0.00, 0.03). In this RCT, SSB intake was a secondary outcome of the intervention (RoB: some concerns) (59). Among observational studies, 1 study reported that SSB intake per 100 g/d at age 8 y was significantly associated with BMI *z*-score change at 11.5 y (β = 0.10; SE = 0.03; *P* = 0.003) (serious RoB) (98). In Peru, daily compared with no intake of SSBs in the past 30 d was associated with greater BMI change (β = 0.74; 95% CI: 0.15, 1.33) and greater risk of overweight/obesity from age 8 y to 12 y [adjusted relative risk (aRR) = 2.12; 95% CI: 1.05, 4.28] (moderate RoB) (91). In US children, SSB intake at 3–5 y was associated with significantly greater odds of overweight/obesity at follow-up (aOR = 1.04; 95% CI: 1.01, 1.07; *P* < 0.05) (moderate RoB) (75). One study examined fruit drinks, non–100% fruit juice, and sodas, with only soda intake (grams per day) significantly associated with BMI (β = 0.011; SE = 0.005; *P* < 0.05) (serious RoB) (63). Eight studies reported no association between SSB intake and outcomes, 5 moderate RoB (57, 64, 76, 88, 129) and 3 serious RoB (62, 99, 116).

##### SSB consumption and BMI and overweight/obesity outcomes (meta-analysis)

**FIGURE 3:**
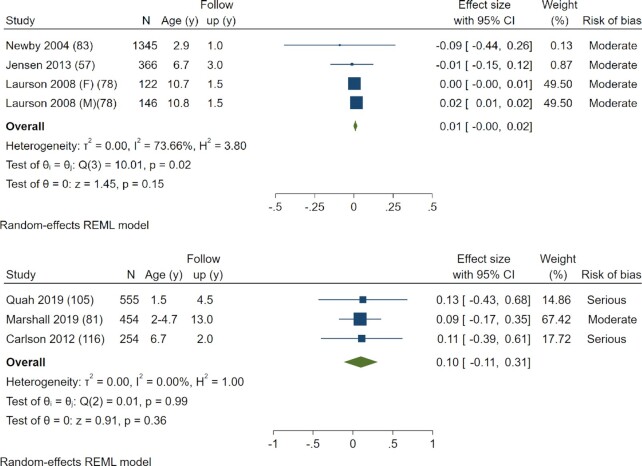
Forest plot of the effect of sugar-sweetened beverage consumption in children aged <10.9 y on BMI. (A) Effect of sugar-sweetened beverage consumption in children aged <10.9 y on BMI change (baseline to follow-up). (B) Effect of sugar-sweetened beverage consumption in children aged <10.9 y on BMI *z*-score values. REML, residual maximum likelihood.

A meta-analysis of 3 studies reporting effects of SSB consumption on change in BMI from baseline to follow-up showed no effect (pooled effect estimate: β = 0.01; 95% CI: −0.00, 0.02) (57, 78, 83 (**Figure 3**A). Heterogeneity across studies was high (*I*^2^ = 73.66%). A meta-analysis of SSB consumption and BMI *z*-score values showed no association (pooled effect estimate: β = 0.10; 95% CI: −0.11, 0.31) (3 included studies) (81, 105, 116) (Figure 3B). There was no heterogeneity across individual studies (*I*^2^ = 0.0%).

##### SSB consumption and percentage body fat outcomes (narrative synthesis)

Seven studies examined SSB consumption and percentage body fat across all age groups **(Supplemental Table 10**). Three of 7 studies reported a significant positive association. SSB intake ≥2 servings/d compared with <1 serving/d at age 5 y was positively associated with higher percentage body fat (ANOVA group: *P* < 0.01;  age: *P* < 0.01;  group × age interaction: *P* < 0.01;  no *F* statistic reported) (serious RoB) (62). High compared with low SSB intake at 6.7 y was associated with higher percentage body fat at 2-y follow-up (β = 1.40; 95% CI: 0.09, 2.72; *P* = 0.036) (serious RoB) (116). Zheng et al. (98) also reported a positive association between SSB intake (per 100 g/d) at 9 y and percentage body fat at 11.5 y (β = 1.04; SE = 0.32; *P* = 0.001) (serious RoB). Four studies reported no association between SSB consumption and percentage body fat, 3 with moderate RoB (72, 95, 128) and 1 with serious RoB (99).

##### SSB consumption and percentage body fat outcomes (meta-analysis)

**FIGURE 4:**
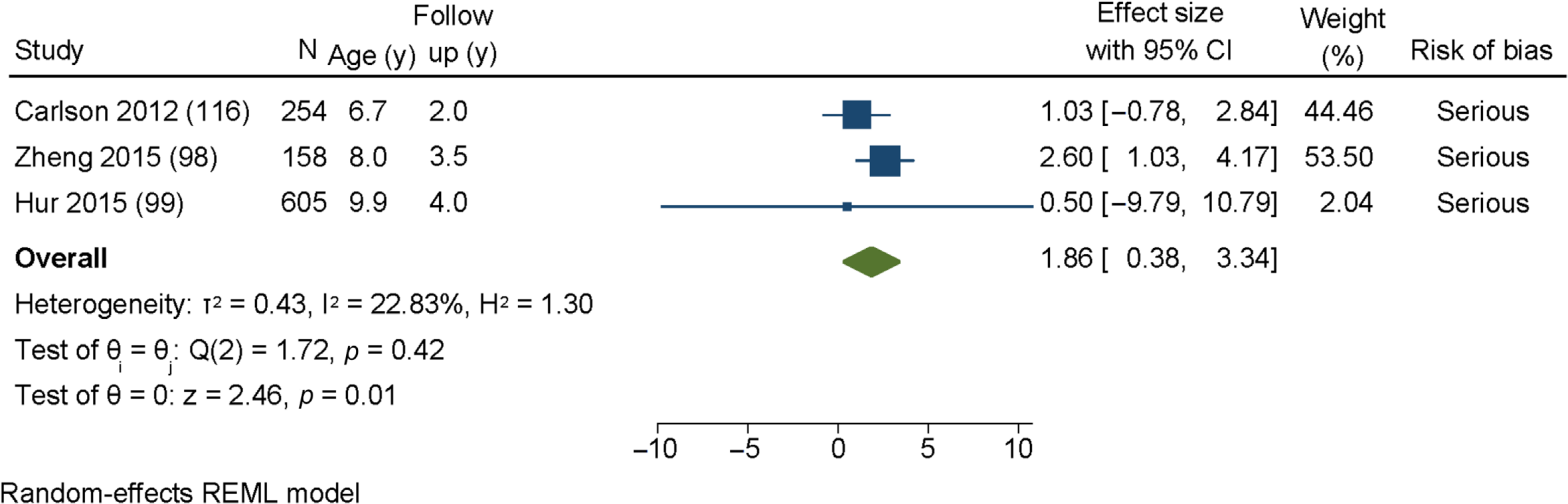
Forest plot of the effect of sugar-sweetened beverage consumption in children aged <10.9 y on percentage body fat. REML, residual maximum likelihood.

**Figure 4** shows the effect estimates for the consumption of SSBs (per 250-mL serving) on percentage body fat. There was a significant positive association between consumption of SSBs and percentage body fat at follow-up (pooled effect estimate: β = 1.86; 95% CI: 0.38, 3.34) in 3 studies (98, 99, 116). Heterogeneity was low (*I*^2^ = 22.8%).

##### Certainty of evidence: SSB consumption

GRADE evidence profiles for the effect of SSB consumption and BMI, overweight/obesity, and percentage body fat are presented in **Table 2**. The certainty of evidence was low for all outcomes except for children aged <2 y where the certainty was very low for overweight/obesity (Table 2). Among observational studies, RoB across studies was assessed as very serious for most age groups due to nonrandomization leading to a likelihood of confounding and selection bias. Inconsistency was judged as not serious, but it was noted that interventions and comparators were different across studies. Indirectness and imprecision were judged as not serious. Evidence was downrated by 1 further level for overweight/obesity in children <2 y because the included studies were all at serious RoB. For the single RCT, the certainty of evidence was also low. In sum, in children ≤10.9 y, the body of evidence indicates that SSB consumption may increase BMI, percentage body fat, or the risk of overweight/obesity (low certainty).

**TABLE 2:** GRADE evidence profile for the effects of sugar-sweetened beverage consumption in children aged ≤10.9 y and BMI, body composition, and overweight/obesity outcomes^1^**Question**: High consumption of SSBs compared with low or no consumption for increased risk of overweight/obesity in children aged ≤10.9 y. **Setting**: All countries, community settings.

Certainty assessment									
Total studies (references)	Study design	Risk of bias	Inconsistency	Indirectness	Imprecision	Other considerations	Impact	Certainty	Importance
Mean BMI/BMI *z*-score or change in BMI/BMI *z*-score in children <2 y at exposure									
3 (105, 112,128)	Observational studies	Very serious^2^	Not serious^3^	Not serious^4^	Not serious^5^	None	Increased BMI (0 studies)Different effects (2 studies, *n* = 3138); different effect in boys vs. girls (105); different effects by age of follow-up: from age 18 mo to 6 y, β: 0.06; 95% CI: −0.20, 0.31; *P =* 0.67; and from age 5 y to 6 y, β: 0.34; 95% CI: 0.11, 0.58; *P =* 0.004 (58)No association (1 study, *n* = 743): mean BMIz difference −0.10; 95% CI: −0·36, 0·16 (112)^6^, ^7^	⨁⨁◯◯ Low	Critical
Mean BMI/BMI *z*-score or change in BMI/BMI *z*-score in children 2 to <5 y at exposure									
6 (70, 72, 73, 81, 83, 93)	Observational studies	Very serious^8^	Not serious^3^	Not serious^4^	Not serious^5^	None	Increased BMI (2 studies, *n* = 4792): β: 0.05; 95% CI: 0.022, 0.079; *P =* 0.001 (81); β: 0.017 ; 95% CI: 0.007, 0.027; *P <* 0.01 (73); No association (4 studies, *n* = 2163): β: −0.01; 95% CI: −0.05, 0.04 ; *P =* 0.852) (70); ANCOVA *P =* 0.0626 (72); β: −0.01 ; SE: 0.02; *P =* 0.50 (83); *P* > 0.05 (93)^6^, ^7^	⨁⨁◯◯ Low	Critical
Mean BMI/BMI *z*-score or change in BMI/BMI *z*-score in children 5 to ≤10.9 y at exposure									
10 (57, 62–64, 76,91,98,99, 116, 129)	Observational studies	Very serious^9^	Not serious^3^	Not serious^4^	Not serious^5^	None	Increased BMI (2 studies, *n* = 158) β: 0·74; 95% CI: 0·15, 1.33 (91); β: 0.10; SE: 0.03; *P =* 0.003 (98); Different effects (1 study, *n* = 2371); positive association for sodas (β: 0.011; SE: 0.005; *P <* 0.05) but not other SSBs (β: 0.009; SE: 0.007; *P* > 0.05) (63)	⨁⨁◯◯ Low	Critical
							No association (7 studies, *n* = 6726); β: 0.11; 95% CI: −0.03, 0.25 (116); ANOVA *P* > 0.05 (62); β: −0.02; SE: 0.03; *P >* 0.05 (99); *P >* 0.05 (parameter estimate from a cross-lagged autoregressive model) (76); intake at 6 y and BMI change 6–9 y, β: −0.014; 95% CI: −0.063, 0.035; *P =* 0.55 (57); boys, β: −0.037; SE: 0.019; *P =* 0.707; girls, β: 0.086; SE: 0.027; *P =* 0.450 (78); at 9 y >1 serve, β: 1.42; SE: 0.68; *P =* 0.29 (64)^6^, ^7^		
Mean change in BMI/BMI *z*-score in children 5 to ≤10.9 y at exposure									
1 (59)	Randomized trial	Serious^10^	Not serious^11^	Serious^12^	Not serious^6^	None	1 study (*n* = 1987) BMI change: β: 0.02; 95% CI: 0.00, 0.03 with each 200-mL glass of sugar-containing beverage consumption/d (59)	⨁⨁◯◯ Low	Critical
Prevalence of overweight and obesity or prevalence of obesity only in children aged <2 y (assessed with: %)									
6 (97, 104,105, 110, 111, 115)	Observational studies	Extremely serious^13^	Not serious^3^	Not serious^4^	Not serious^5^	None	Increased risk (3 studies, *n* = 3372); aOR: 2.99; 95% CI: 1.27, 7.00 (115); ≥3 times/wk, aOR = 2.00; 95% CI: 1.02, 3.90 (104); aOR: 1.6; 95% CI: 1.04, 1.93; *P <* 0.01 (110); Different effects (2 studies, *n* = 7567); at 2 y no association, at 5 y aOR: 2.3; 95% CI: 1.4, 3.7 (97); at 18 m no association, at 5 y RR: 1.10; 95% CI: 0.67, 1.81; *P =* 0.204 (105); No association (1 study, *n* = 1871); aOR: 0.91; 95% CI: 0.44, 1.88 (111)	⨁◯◯◯ Very low	Critical
Prevalence of overweight and obesity or prevalence of obesity only in children aged 2 to <5 y (assessed with: %)									
5 (58, 74, 80, 96, 118)	Observational studies	Very serious^14^	Not serious^3^	Not serious^4^	Not serious^5^	None	Increased risk (1 study, *n* = 473); aOR: 1.92; 95% CI: 1.19, 3.11; *P* ≤ 0.01 (118); Different effects (1 study, *n* = 2986); overweight/obesity no association, obesity only aOR: 1.65; 95% CI: 1.12, 2.44; *P =* 0.01 (58); No association (3 studies, *n* = 17083); no association (no estimate) (96); aOR: 1.3; 95% CI: 0.8, 2.1 (58); boys aOR: 1.01; 95% CI: 0.8, 1.29; girls aOR: 1.08; 95% CI: 0.87, 1.35 (74)	⨁⨁◯◯ Low	Critical
Prevalence of overweight and obesity or prevalence of obesity only in children aged 5 to ≤10.9 y (assessed with: %)									
3 (75, 88, 91)	Observational studies	Very serious^8^	Not serious^3^	Not serious^4^	Not serious^5^	None	Increased risk (2 studies, *n* = 1668): aRR: 2.12; 95% CI: 1.05, 4.28 (91); aOR: 1.04; 95% CI: 1.01, 1.07; *P* < 0.05 (75); No association (1 study, *n* = 1250); overweight only, aOR: 1.29; 95% CI: 0.84, 1.96; *P =* 0.246; obese only, aOR: 1.57; 95% CI: 0.82, 3.03; *P =* 0.177 (88)	⨁⨁◯◯ Low	Critical
Prevalence of overweight and obesity or prevalence of obesity only in children aged 5 to ≤10.9 y (assessed with: %)									
1 (59)	Randomized trial	Serious^10^	Not serious^11^	Serious^12^	Not serious^5^	None	Increased risk (1 study, *n* = 1987); each 200-mL glass/d of sugar-sweetened beverage consumption increased the odds of obesity (aOR: 1.22; 95% CI: 1.04, 1.44; *P =* 0.014) but not overweight (*P =* 0.83)	⨁⨁◯◯ Low	Critical
Mean percentage body fat in children aged ≤10.9 y									
7 (62, 72, 95, 98, 99, 116, 128)	Observational studies	Very serious^15^	Not serious^3^	Not serious^4^	Not serious^5^	None	Increased percentage body fat (3 studies, *n* = 578); ANOVA *P* < 0.01 (62); β: 1.40; 95% CI: 0.09, 2.72; *P =* 0.036 (116); β: 1.04; SE: 0.32; *P =* 0.001 (98); No association (4 studies, *n* = 3436) ANCOVA *P =* 0.929 (72); β: 0.02; SE: 0.21; *P >* 0.05 (99); β: −0.15; 95% CI: −0.54, 0.24; *P =* 0.45 (95); boys, β: 0.05 ; 95% CI: −0.11, 0.20; *P =* 0.53; girls, β: 0.09 ; 95% CI: −0.06, 0.23; *P =* 0.25 (128)^16^	⨁⨁◯◯ Low	Critical

1 aOR, adjusted odds ratio; aRR, adjusted risk ratio; BMIz, body mass index *z*-score; GRADE, Grading of Recommendations Assessment, Development, and Evaluation; RCT, randomized controlled trial; SSB, sugar-sweetened beverage.

2 Risk of bias was moderate in 1 study (112) and serious in 2 studies (105, 128). Downgraded by 2 levels due to nonrandomization leading to confounding and selection bias.

3 Not downgraded for inconsistency but note that interventions and comparators were different across studies.

4 Not downgraded because study populations, exposures, and comparators were relevant to review question, although no studies were from low-income country populations.

5 Not downgraded because no evidence of imprecision (i.e., not wide CIs, small sample size, or low number of events).

6 Meta-analysis of 3 studies across different age groups: BMI change effect size 0.01–0.00, 0.02) (57, 78, 83).

7 Meta-analysis of 3 studies across different age groups: BMI *z*-score change effect size 0.10; 95% CI: −0.11, 0.31 (81, 105, 116).

8 Risk of bias was moderate for all studies. Downgraded by 2 levels due to nonrandomization in observational studies leading to confounding and selection bias.

9 Risk of bias was moderate in 5 studies (46, 76, 78, 91, 98) and serious in 5 studies (62, 63, 98, 99, 116). Downgraded by 2 levels due to nonrandomization in observational studies leading to confounding and selection bias.

10 Some concerns due to missing outcome data and bias in selection of reported result.

11 Not downgraded because only 1 study.

12 Downgraded by 1 level because SSB consumption was a secondary outcome of the RCT.

13 Risk of bias was serious for all 5 studies. Downgraded by 2 levels for inherent risk of bias due to nonrandomization and 1 further level due to serious risk of bias in all studies.

14 Risk of bias was moderate in 4 studies (58, 74, 96, ) and serious in 1 study (118).

15 Risk of bias was moderate in 3 studies (72, 95, 128), serious in 4 studies (62, 98, 99, 116). Downgraded by 2 levels for risk of bias due to nonrandomization in observational studies leading to confounding and selection bias.

16 Meta-analysis of 3 studies (98, 99, 116): pooled effect estimate β: 1.86; 95% CI: 0.38, 3.34.

**TABLE 3:** GRADE evidence profile for the effects of artificially sweetened beverage consumption in children aged ≤10.9 y and BMI, body composition, and overweight/obesity outcomes^1^**Question**: High consumption of artificially sweetened beverages compared with low or no consumption of artificially sweetened beverages for increased risk of overweight/obesity in children ≤10.9 y. **Setting**: All countries, community settings.

Certainty assessment									
Total studies (references)	Study design	Risk of bias	Inconsistency	Indirectness	Imprecision	Other considerations	Impact	Certainty	Importance
Mean BMI/BMI *z*-score or change in BMI/BMI *z*-score in children <2 y at exposure									
0							No included studies		
Mean BMI/BMI *z*-score or change in BMI/BMI *z*-score in children 2 to <5 y at exposure									
2 (72, 83)	Observational studies	Very serious^2^	Not serious^3^	Not serious^4^	Not serious^5^	None	Increased BMI (0 studies); No association (2 studies, *n* = 1443): ANCOVA *P =* 0.444 (72); β: 0.01; SE: 0.02; *P =* 0.83 (83)	⨁⨁◯◯ Low	Critical
Mean BMI/BMI *z*-score or change in BMI/BMI *z*-score in children 5 to ≤10.9 y at exposure									
2 (63, 98)	Observational studies	Extremely serious^6^	Not serious^3^	Not serious^4^	Not serious^5^	None	Increased BMI (0 studies); No association (1 study, *n* = 2371); β: 0.01; SE: 0.013; *P* > 0.05 (63); Decreased BMI (1 study, *n* = 158); β: −0.20; SE: 0.07; *P =* 0.01 (98)	⨁◯◯◯ Very low	Critical
Prevalence of overweight and obesity or prevalence of obesity only in children aged <2 y (assessed with: %)									
0							No included studies	—	
Prevalence of overweight and obesity or prevalence of obesity only in children aged 2 to <5 y (assessed with: %)									
1 (80)	Observational studies	Very serious^2^	Not serious^7^	Not serious^4^	Not serious^5^	None	Increased risk (0 studies); Different effects (1 study, *n* = 2986) no association with overweight/obesity (aOR: 0.85; 95% CI: 0.63, 1.15; *P =* 0.85) but increased risk of obesity (aOR: 1.57; 95% CI: 1.05, 2.36; *P =* 0.03) (58)	⨁⨁◯◯ Low	Critical
Prevalence of overweight and obesity or prevalence of obesity only in children aged 5 to ≤10.9 y (assessed with: %)									
0							No included studies	—	
Mean percentage body fat in children aged ≤10 y (assessed with: %)									
3 (72, 95, 98)	Observational studies	Very serious^8^	Not serious^3^	Not serious^4^	Not serious^5^	None	Increased percentage body fat (1 study, *n* = 362) β: 0.26; 95% CI: −0.004, 0.52; *P =* 0.05 (95); No association (1 study, *n* = 98): ANCOVA *P =* 0.584 (72)	⨁⨁◯◯ Low	Critical
							Negative association (1 study, *n* = 158) β: −1.41; SE: 0.70; *P =* 0.046 (98)		

1 aOR, adjusted odds ratio; GRADE, Grading of Recommendations Assessment, Development, and Evaluation.

2 Risk of bias was moderate for all studies. Downgraded by 2 levels for inherent bias due to nonrandomization in observational studies leading to confounding and selection bias.

3 Not downgraded for inconsistency but note that there were differences between interventions and comparators across studies.

4 Not downgraded because study populations, exposures, and comparators were relevant to review question, although no studies were from low-income country populations.

5 Not downgraded because no evidence of imprecision (i.e., not wide CIs, small sample size, or low number of events).

6 Risk of bias was serious for all studies. Downgraded by 2 levels for risk of bias due to nonrandomization (confounding and selection bias) and 1 further level for serious risk of bias across the body of evidence.

7 Not downgraded because only 1 study.

8 Risk of bias was moderate in 2 studies (72, 95) and serious in 1 study (98). Downgraded by 2 levels due to risk of bias due to nonrandomization in observational studies leading to confounding and selection bias.

#### ASB consumption and BMI, overweight/obesity, and body composition outcomes

Seven studies reported ASB consumption in relation to all child growth, body composition, and overweight/obesity outcomes. Four studies defined the exposure as diet sodas (63, 83, 98, 120), 2 used the term “ASB” (72, 80), and 1 referred to reduced sugar, or sugar-free fruit squashes, cordials, and diet sodas (95).

##### ASB consumption and BMI and overweight/obesity outcomes (narrative synthesis)

Six studies examined ASB consumption and BMI or overweight/obesity (Supplemental Table 9). One was assessed as critical RoB (120). No included studies examined ASB consumption in children aged <2 y. Of the 5 studies with included results, 1 observed an inverse association between ASB intake (grams per day) and BMI *z*-score change (β = −0.20; SE = 0.07; *P* = 0.01) (serious RoB) (98). Three of 5 studies reported no association between ASB intake and BMI, 2 with moderate RoB (72, 83), 1 with serious RoB (63). One study reported no difference in odds of overweight/obesity combined but greater odds of obesity with high ASB consumption (once per day) compared with low (less than once per week or never) (aOR = 1.57; 95% CI: 1.05, 2.36; *P* = 0.03) (moderate RoB) (80).

##### ASB consumption and percentage body fat outcomes (narrative synthesis)

Three studies examined ASB intake in relation to body fat (Supplemental Table 10). One reported a negative association (per 100 g/d) (serious RoB) (β = −1.41; SE = 0.70; *P* = 0.046) (98) and 2 reported no association, both moderate RoB (72, 95).

##### Certainty of evidence: ASB consumption

There was no evidence on the effects of ASB consumption on children <2 y. The certainty of evidence for effects of ASB consumption in children aged 2 to <5 y was low for BMI and overweight/obesity (**Table 3**). In children aged 5 to ≤10.9 y, the certainty of evidence was very low for BMI and there was no evidence for overweight/obesity. Certainty of evidence for ASB consumption and percentage body fat was low. Therefore, the body of evidence for all age groups ≤10.9 y indicates that ASB consumption makes little or no difference to increased BMI, percentage body fat, or the risk of overweight/obesity (low certainty).

#### One hundred percent fruit juice consumption and BMI, overweight/obesity, and body composition outcomes

Seventeen studies reported effects of fruit juice consumption. In 16 studies, the exposure was specified as 100% juice. One study examined unsweetened fruit juice and small intakes of sweetened fruit and vegetable juice (72). This study was placed with 100% fruit juice for the synthesis because this was the closest match. Two studies were judged as critical RoB (120, 124).

##### One hundred percent fruit juice consumption and BMI and overweight/obesity outcomes (narrative synthesis)

Ten studies assessed fruit juice consumption and BMI or overweight/obesity (Supplemental Table 9). Nine of the 10 studies reported no association (5 moderate, 4 serious RoB) (58, 72, 81, 83, 87, 98, 114, 116, 130). One study reported mixed results, with fruit juice intake from 2 to 4 y associated with greater BMI *z*-score change from baseline to 4 y [mean change = 0.282, SE = 0.028, compared with 0.030, SE = 0.037; *P* = 0.0003 for groups ≥1 serving (236.5 mL)/d compared with <1 serving/d], but not from 4 to 5 y (mean change = 0.034, SE = 0.031, compared with 0.020, SE= 0.021; *P* = 0.6778) (moderate RoB) (86). In the same study, odds of overweight were not associated with juice intake in normal weight or risk-of-overweight children at baseline (moderate RoB) (86).

##### One hundred percent fruit juice consumption and BMI and overweight/obesity outcomes (meta-analysis)

**FIGURE 5:**
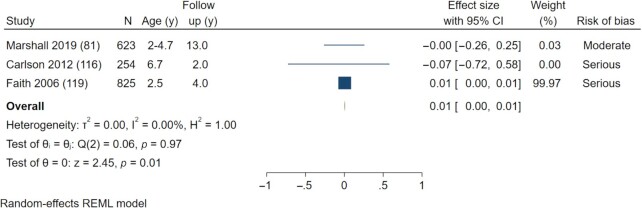
Forest plot of the effect of 100% juice consumption in children aged <10.9 y on BMI *z*-score values. REML, residual maximum likelihood.

**Figure 5** shows the effect estimates for the consumption of 100% fruit juice (per 250-mL serving) on BMI *z*-score for 3 studies (81, 116, 119). The pooled effect estimate was positive but small (β=0.01; 95% CI: 0.00, 0.01). There was no heterogeneity across individual studies (*I*^2^ = 0.0%).

##### One hundred percent juice consumption and percentage body fat outcomes

Four studies reported effects of 100% juice consumption on percentage body fat (Supplemental Table 10); all 4 studies reported no association (2 moderate, 2 serious RoB) (72, 95, 98, 116).

##### Certainty of evidence: 100% juice consumption

GRADE evidence profiles for effects of 100% fruit juice consumption are shown in **Table 4**. The certainty of evidence for BMI, overweight/obesity, and percentage body fat was low, with the exception of children aged 5 to ≤10 y, where the certainty was very low for BMI and there was no evidence for overweight/obesity (Table 4). The body of evidence for all age groups ≤10.9 y indicates that 100% fruit juice consumption makes little or no difference to increased BMI, percentage body fat, or the risk of overweight/obesity (low certainty).

**TABLE 4:** GRADE evidence profile for the effects of 100% fruit juice consumption in children aged ≤10.9 y and BMI, body composition, and overweight/obesity outcomes^1^**Question**: High consumption of 100% fruit juice compared with low or no consumption of 100% fruit juice for increased risk of overweight/obesity in children ≤10.9 y. **Setting**: All countries, community settings.

Certainty assessment									
Total studies (references)	Study design	Risk of bias	Inconsistency	Indirectness	Imprecision	Other considerations	Impact	Certainty	Importance
Mean BMI/BMI *z*-score or change in BMI/BMI *z*-score in children <2 y at exposure									
1 (87)	Observational study	Very serious^2^	Not serious^3^	Not serious^4^	Not serious^5^	None	Increased BMI (0 studies); No association (1 study, *n* = 1038) β: 0.30; 95% CI: −0.01, 0.61 at 2.1 y follow-up; β: 0.27; 95% CI: −0.05, 0.59 at 6.7 y follow-up (87)^6^	⨁⨁◯◯ Low	Critical
Mean BMI/BMI *z*-score or change in BMI/BMI *z*-score in children 2 to <5 y at exposure									
5 (67, 72, 81, 83, 86)	Observational studies	Very serious^7^	Not serious^8^	Not serious^4^	Not serious^5^	None	Increased BMI (0 studies); Different effects (1 study, *n* = 6250): mean BMI *z*-score change = 0.282 (SE: 0.028) vs. 0.030 (SE: 0.037), *P =* 0.0003 at 2–4 y, 0.034 (SE: 0.031) 0.020 (SE: 0.021) *P =* 0.6778 at 4–5 y (86); No association (4 studies, *n* = 2138): ANCOVA *P =* 0.062 (72); β: 0.01; SE: 0.00; *P =* 0.20 (83); β: −0.001; 95% CI: −0.059, 0.057; *P =* 0.97 (81); β: −0.057; *P =* 0.099 (SE not stated) (67)^6^	⨁⨁◯◯ Low	Critical
Mean BMI/BMI *z*-score or change in BMI/BMI *z*-score in children 5 to ≤10.9 y at exposure									
2 (98, 116)	Observational studies	Extremely serious^9^	Not serious^8^	Not serious^4^	Not serious^5^	None	Increased BMI (0 studies); No association (2 studies, *n* = 412): β: −0.04, 95% CI: −0.21, 0.13; *P =* 0.631 (116); β: 0.07; SE: 0.05; *P =* 0.12 (98)^6^	⨁◯◯◯ Very low	Critical
Prevalence of overweight and obesity or prevalence of obesity only in children aged <2 y (assessed with: %)									
1 (87)	Observational study	Extremely serious^9^	Not serious^3^	Not serious^4^	Not serious^5^	None	Increased risk (0 studies); No association (1 study, *n =* 1076); odds of overweight including obesity, aOR: 1.0; 95% CI: 0.5, 2.0; *P =* 0.916 (114)	⨁◯◯◯ Very low	Critical
Prevalence of overweight and obesity or prevalence of obesity only in children aged 2 to<5 y (assessed with: %)									
2 (58, 86)	Observational studies	Very serious^2^	Not serious^8^	Not serious^4^	Not serious^5^	None	Increased risk (0 studies); Different effects (1 study, *n* = 6250); overweight/obesity aOR: 1.30; 95% CI: 1.06, 1.59; *P =* 0.0129 at 2–4 y follow-up; aOR: 0.80; 95% CI: 0.43, 1.49; *P* = 0.473 at 4–5 y follow-up) (86); No association (1 study, *n* = 10,904): high vs. low intake in normal weight at baseline, aOR: 1.2; 95% CI: 0.8, 1.7); high vs. low intake in at risk of overweight at baseline, aOR: 0.8; 95% CI: 0.5, 1.1 (58)	⨁⨁◯◯ Low	Critical
Prevalence of overweight and obesity or prevalence of obesity only in children aged 5 to ≤10.9 y (assessed with: %)									
0							No included studies	—	
Mean percentage body fat in children aged ≤10.9 y (assessed with: %)									
4 (72, 95, 98, 116)	Observational studies	Very serious^10^	Not serious^8^	Not serious^4^	Not serious^5^	None	Increased percentage body fat (0 studies); No association (4 studies, *n* = 872); β: −1.06; 95% CI: −2.70, 0.57; *P =* 0.202 (116); ANCOVA *P =* 0.119 (72); β: −0.11; 95% CI:	⨁⨁◯◯ Low	
							−0.61, 0.38; *P =* 0.66 (95); β: −0.05; SE: 0.44; *P =* 0.91 (98)		

1 aOR, adjusted odds ratio; GRADE, Grading of Recommendations Assessment, Development, and Evaluation.

2 Risk of bias was moderate in all studies. Downgraded by 2 levels due to nonrandomization in observational studies leading to confounding and selection bias.

3 Not downgraded because only 1 study.

4 Not downgraded because study populations, exposures, and comparators were relevant to review question, although no studies were from low-income country populations.

5 Not downgraded because no evidence of imprecision (i.e., not wide CIs, small sample size, or low number of events).

6 Meta-analysis of 3 studies across age groups on BMI *z*-score effect size: 0.01; 95% CI: 0.00, 0.01.

7 Risk of bias was moderate in 4 studies (72, 81, 83, 86) and serious in 1 study (67). Downgraded by 2 levels due to nonrandomization in observational studies leading to confounding and selection bias.

8 Not downgraded for inconsistency but note that interventions and comparators were not the same across studies.

9 Risk of bias was serious in all studies. Downgraded by 2 levels for inherent risk of bias due to nonrandomization and 1 further level due to body of evidence based on studies at serious risk of bias.

10 Risk of bias was moderate in 2 studies (72, 95) and serious in 2 studies (98, 116). Downgraded by 2 levels due to nonrandomization leading to bias due to confounding and selection bias.

#### Unhealthy food item consumption and BMI, overweight/obesity, and body composition outcomes

Twenty-six studies reported effects of unhealthy food consumption on growth, body composition, or overweight/obesity outcomes with a range of exposures. Consumption of high-fat foods was assessed in 4 studies (5 articles) (73, 74, 103, 108, 116). Six studies (7 articles) examined the intake of free sugars or added sugar or sweetened foods (85, 99, 101, 102, 112, 121, 127). Fast food consumption was examined in 5 studies (69, 71, 76, 111, 113). Three studies reported on UPF consumption (70, 90, 131). Other exposures included salty snacks (91), sweets (125), or combinations of both (82, 118). Studies were predominantly conducted in HIC. Studies from MIC were conducted in Brazil and Peru (70, 89–91, 131). Four of the 26 studies were assessed as being at critical RoB and are not reported further (61, 121, 125, 126).

##### Unhealthy foods and BMI and overweight/obesity outcomes (narrative synthesis)

Of the 22 included studies examining unhealthy food consumption, 16 studies reported BMI outcomes or overweight and obesity prevalence (Supplemental Table 9).

In children aged <2 y, 4 studies examined unhealthy foods. Of these, 1 observed a positive association between sweet foods consumption from 3 to 12 mo and weight-for-length *z*-scores at 3 y (ANOVA, *F* = 3.23, *P* = 0.03), but no association with snack foods (moderate RoB) (82). The remaining 3 studies (1 at moderate and 2 at serious RoB) found no associations between unhealthy foods (“extra food,” fast food and snacks, sweetened first foods) and BMI or overweight/obesity (29, 111, 112). In children aged 2 to <5 y, there were 7 studies (10 articles). Two studies reported a positive association with unhealthy food consumption and outcomes. Consumption of added sugar to milk and fruits was associated with significantly higher BMI in boys and girls aged 2 to <6 y at baseline, but in older children (6 to <10 y) the association was only significant in boys (no effect estimate available) (moderate RoB) (85). Frequency of fast food intake (high or low) was associated with increased risk of change in BMI status (normal to overweight, or overweight to obese) in children aged 3–5 y followed up 1 y later (RR: 1.38; 95% CI: 1.13, 1.67; *P* < 0.01) (moderate RoB) (69).

In children aged 2 to <5 y, 3 of the 7 studies presented results that differed by quantity consumed, outcome, or time point. Consumption of high-fat food was associated with significantly higher BMI *z*-scores (73), but not with odds of overweight and obesity (moderate RoB) (74). In a study in Brazil, frequency of energy-dense food consumption was not associated with BMI *z*-scores (89), but the percentage energy intake from UPFs at age 4 y was significantly associated with BMI *z*-score at 7 y, whereas intake at 7 y was not (moderate RoB) (90). One study reported no effects of added sugar at age 2 y on change in BMI *z*-score at 5 and 6 y of age. A separate analysis from the same study found that consumption at age 1 y was not associated with change in BMI *z*-score at 7 y, but change in intake from 1 to 7 y was significantly associated with change in BMI *z*-scores (serious RoB) (101, 102). The remaining 2 of 7 studies reported no association between unhealthy food consumption and BMI or overweight and obesity (1 moderate, 1 serious RoB) (70, 118).

Five studies examined effects of unhealthy food consumption in children aged 5 to ≤10.9 y. One reported an association between salty, high-fat snack frequency with change in BMI from 8 y to 12 y (β = 0.71; 95% CI: 0.14, 1.28; *P* < 0.05) (moderate RoB) (91). Bel-Serrat et al. (113) found significantly lower odds of overweight/obesity with savory snack intake some days per week (aOR = 0.48; 95% CI: 0.23, 0.99; *P* < 0.05) or never (OR = 0.27; 95% CI: 0.10, 0.72; *P* < 0.01) compared with every day, but no association between fast food intake and overweight/obesity (serious RoB). Three of the 5 studies of ages 5 to ≤10.9 y reported no association between unhealthy food intake and BMI or overweight/obesity outcomes (1 moderate, 2 serious RoB) (76, 99, 116).

##### Unhealthy food consumption and percentage body fat outcomes

Four studies (5 articles) examined unhealthy food consumption in relation to body fat. Three studies measured percentage body fat and reported no association with unhealthy food consumption (all serious RoB) (99, 101, 102, 116) (Supplemental Table 10). One study examined fat mass index and reported an association between annual consumption of UPFs (in grams via 12-mo recall) in children aged 6 y at baseline and higher fat mass index at 5-y follow-up (β = 0.05; 95% CI: 0.04, 0.06; *P* < 0.001) (moderate RoB) (131).

##### Certainty of evidence: unhealthy foods

GRADE evidence profiles for the effects of consumption of unhealthy foods are presented in **Table 5**. The certainty of evidence for BMI/BMI *z*-score and overweight/obesity was low, with the exception of children aged <2 y, where the certainty was very low for overweight/obesity. The certainty of evidence for percentage body fat across all ages ≤10.9 y was very low (Table 5).

**TABLE 5:** GRADE evidence profile for the effects of consumption of unhealthy food items in children aged ≤10.9 y and growth, body composition, and overweight/obesity outcomes^1^**Question**: High consumption of unhealthy food items compared with low or no consumption of unhealthy food items for increased risk of overweight/obesity in children ≤10 y. **Setting**: All countries, community settings.

Certainty assessment									
Total studies (references)	Study design	Risk of bias	Inconsistency	Indirectness	Imprecision	Other considerations	Impact	Certainty	Importance
Mean BMI/BMI *z*-scores or change in BMI/BMI *z*-scores in children aged <2 y									
3 (29, 82,112)	Observational studies	Very serious^2^	Not serious^3^	Not serious^4^	Not serious^5^	None	Increased BMI (1 study, *n =* 666): candies, ANOVA *F* = 3.23, *P =* 0.03 (82); No association (2 studies, *n =* 1105); “extra foods” β: −0.10; 95% CI: −0.30, 0.11; *P =* 0.36 (29); sweetened foods BMIz mean difference = 0.03; 95% CI: −0.12, 0.19 (112)	⨁⨁◯◯ Low	Critical
Mean BMI/BMI *z*-scores or change in BMI/BMI *z*-scores in children aged 2 to <5 y at exposure									
6 (69, 70, 73,85, 90, 101)	Observational studies	Very serious^6^	Not serious^3^	Not serious^4^	Not serious^5^	None	Increased BMI (3 studies, *n =* 11639); fast foods, aRR: 1.38; 95% CI: 1.13, 1.67; *P* < 0.01 (69); high-fat foods, β: 0.021; 95% CI: 0.014, 0.029; *P* < 0.001 (73); sugar-added to foods 2 < 6 y: boys, *P =* 0.005; girls, *P =* 0.03; 6 < 10 y: boys, *P =* 0.001; girls, *P* > 0.05 (85); Different effects (1 study, *n =* 1175); ultraprocessed food intake at 4 y, β: 0·028; 95% CI: 0.006, 0.051; intake at 7 y β: 0·014; 95% CI: −0.007, 0.036 (90); No association (2 studies, *n =* 695); added sugar, β: −0.001; SE: 0.010; *P =* 0.9 (101); ultraprocessed foods, β: 0.05; 95% CI: −0.04, 0.15; *P =* 0.282 (70)	⨁⨁◯◯ Low	Critical
Mean BMI/BMI *z*-scores or change in BMI/BMI *z*-scores in children aged 5 to ≤10.9 y at exposure									
4 (76, 91, 99,116)	Observational studies	Very serious^7^	Not serious^3^	Not serious^4^	Not serious^5^	None	Increased BMI (1 study, *n* = 1414); snack foods β: 0.71; 95% CI: 0.14, 1.28 (91)No significant association (3 studies, *n =* 5797); high-fat foods, β: −0.02; 95% CI: −0.06, 0.03; *P =* 0.409 (116); other sugars, β: 0.16; SE: 0.10; *P >* 0.05 (99); fast foods, *P* > 0.05 (parameter estimate from a cross-lagged autoregressive model) (76)	⨁⨁◯◯ Low	Critical
Prevalence of overweight and obesity or prevalence of obesity only in children aged <2 y (assessed with: %)									
1 (82)	Observational studies	Extremely serious^8^	Not serious^3^	Not serious^4^	Not serious^5^	None	Increased risk (0 studies); No association (1 study, *n* = 1871) fast foods, aOR: 1.14; 95% CI: 0.77, 1.67; snack consumption, aOR: 0.71; 95% CI: 0.52, 0.98 (82)	⨁◯◯◯ Very low	Critical
Prevalence of overweight and obesity or prevalence of obesity only in children aged 2 to <5 y (assessed with: %)									
2 (74, 118)	Observational studies	Very serious^9^	Not serious^3^	Not serious^4^	Not serious^5^	None	Increased risk (0 studies); No association (2 studies, *n =* 4680); sweet and savory snacks, aOR: 0.76; 95% CI: 0.41, 1.40; *P >* 0.05 (118); high-fat foods boys, aOR: 0.85; 95% CI: 0.6, 1.19; girls: aOR: 0.97; 95% CI: 0.7, 1.35 (74)	⨁⨁◯◯ Low	Critical
Prevalence of overweight and obesity or prevalence of obesity only in children aged 5 to ≤10 y (assessed with: %)									
2 (91, 113)	Observational studies	Very serious^10^	Not serious^11^	Not serious^4^	Not serious^5^	None	Increased risk (0 studies); Different effects (1 study, *n* = 2755); savory snacks never vs.	⨁⨁◯◯ Low	Critical
							everyday, aOR: 0.27; 95% CI: 0.10, 0.72; *P* < 0.01; fast food never vs. everyday, aOR: 0.91; 95% CI: 0.19, 4.31; *P* > 0.05 (113); No association (1 study, *n =* 1414); savory snacks, aRR: 1.43; 95% CI: 0.78, 2.69 (91)		
Percentage body fat ≤10 y									
4 (99, 101,116, 131)	Observational studies	Extremely serious^12^	Not serious^3^	Not serious^4^	Not serious^5^	None	Increased percentage body fat (1 study, *n =* 3514); ultraprocessed foods, β: 0.05; 95% CI: 0.04, 0.06; *P* < 0.001 (NOTE: fat mass index, not % body fat) (131); No association (3 studies, *n =* 1239); added sugar, β: 0.048; SE: 0.046; *P =* 0.3 (101); high-fat foods, β: −0.38, 95% CI: −0.81, 0.05; *P =* 0.081 (116); other sugars, β: 0.83; SE: 0.72; *P* > 0.05 (99)	⨁◯◯◯ Very low	Critical

1 aOR, adjusted odds ratio; aRR, adjusted relative risk; BMIz, body mass index *z*-score; GRADE, Grading of Recommendations Assessment, Development, and Evaluation.

2 Risk of bias was moderate in 2 studies (29, 82), serious in 1 study (112). Downgraded by 2 levels for risk of bias due to nonrandomization in observational studies leading to confounding and selection bias.

3 Not downgraded for inconsistency but note that interventions and comparators were different across studies.

4 Not downgraded because study populations, exposures, and comparators were relevant to review question, although no studies were from low-income country populations.

5 Not downgraded because no evidence of imprecision (i.e., not wide CIs, small sample size, or low number of events).

6 Risk of bias was moderate in 5 studies (69, 70, 73, 85, 90) and serious in 1 study (101). Downgraded by 2 levels for risk of bias due to nonrandomization in observational studies leading to confounding and selection bias.

7 Risk of bias was moderate in 2 studies (76, 91) and serious in 2 studies (99, 116). Downgraded by 2 levels for risk of bias due to nonrandomization in observational studies leading to confounding and selection bias.

8 Risk of bias was serious in all studies (111). Downgraded by 2 levels for nonrandomization in observational studies leading to confounding and selection bias, and 1 level further due to body of evidence all from studies with serious risk of bias.

9 Risk of bias was moderate in 1 study and serious in 1 study. Downgraded by 2 levels for inherent risk of bias due to nonrandomization in observational studies.

10 Risk of bias was moderate in 1 study (91) and serious in 1 study (113). Downgraded by 2 levels for risk of bias due to nonrandomization leading to confounding and selection bias.

11 Not downgraded because only 1 study.

12 Risk of bias was moderate for 1 study (131) and serious for 3 studies (99, 101, 116). Downgraded by 2 levels for risk of bias due to nonrandomization leading to confounding and selection bias and 1 level further due to majority of the body of evidence had serious risk of bias.

Synthesis of results of unhealthy food and beverage consumption and other growth and body composition outcomes can be found in **Supplemental Table 11**.

## Discussion

### Summary of evidence

In this review of the effects of unhealthy food and beverage consumption on risk of overweight and obesity, we found no studies in low-income countries, and a paucity of evidence in children aged 0–2 y. Previous survey data from 18 countries indicate that consumption of SSBs and sugary snacks is high in LMIC, with ≤75% of children in Asia and 46% of children in Africa consuming such foods at ages 12–23 mo (1). Despite this, the effect of unhealthy food and beverage consumption in infancy and childhood remains poorly understood, particularly in LMIC settings where diets are rapidly changing, and where multiple forms of malnutrition coexist (5). Prospective studies are needed in LMIC on the amounts and types of foods consumed in relation to nutritional outcomes. This review also highlights a lack of robust studies purposefully designed to assess the effects of unhealthy food and beverage consumption in childhood on growth outcomes. High-quality and standardized data are needed in order to make nutritional recommendations to prevent all forms of malnutrition.

The largest body of evidence in this review was on the effects of SSB consumption. For children ≤10.9 y, the body of evidence indicates that SSB consumption may increase BMI, percentage body fat, and risk of overweight/obesity (low certainty). This accords with review findings for infants aged 6–23 mo (13). Meta-analyses in the present review indicated a positive association between SSB intake and percentage body fat, but no association with change in BMI and BMI *z*-score; however, the number of pooled studies was small. A previous systematic review reported that BMI increased by 0.07 (95% CI: 0.01, 0.12) for each additional daily serving (∼354 mL) of SSBs in children and adolescents, but heterogeneity was high (*I*^2^ = 91.6%; *P* < 0.001) (14). Some RCTs have examined the effects of SSB consumption by comparing with a group receiving ASBs (132–134). One 18-mo RCT reported lower BMI increases in children receiving ASBs compared with SSBs, but on an intention-to-treat basis there was no significant difference in BMI *z*-score increase between the 2 groups (132). Such studies did not meet eligibility criteria for this review because they compared 2 items on the review list of exposures (SSBs and ASBs) with no control group.

We found that consumption of ASBs and consumption of 100% fruit juice in children ≤10.9 y makes little or no difference to increased BMI, percentage body fat, or risk of overweight/obesity (low certainty). For ASB consumption, no evidence was available for children <2 y. For 100% juice consumption, the pooled estimate from meta-analysis in this review (β = 0.01; 95% CI: 0.00, 0.01) accords with a systematic review of longitudinal studies of 100% fruit juice consumption in children aged 1 to 6 y where a 1 serving increment was associated with a 0.087 (95% CI: 0.008, 0.167) unit increase in BMI *z*-score, considered not clinically significant (15). Importantly, our review ensured that all included evidence was for 100% juice consumption only, whereas some reviews have included evidence from juice drinks where the proportion of fruit juice varied or was unstated (13).

### Unhealthy food consumption

Studies reporting unhealthy food consumption assessed salty, high-fat food consumption (73, 74, 91, 113), UPFs (90, 131), fast food or “extra foods” (29, 69, 111), and added sugars or foods high in sugars (85, 101, 112). We found consumption of unhealthy foods in children ≤10.9 y may increase BMI, percentage body fat, or risk of overweight/obesity (low to very low certainty). However, no meta-analysis was possible due to the high heterogeneity of reporting exposures and comparators. A systematic review of complementary feeding (6–23 mo) found insufficient evidence of effects of consumption of unhealthy foods (13), concurring with the findings of the present study for all children ≤10.9 y. A previous systematic review of UPF consumption and body fat in children and adolescents included both longitudinal and cross-sectional study designs and therefore reverse causality was a possible underlying factor in observed associations (16).

### Limitations of the evidence

A major limitation of the evidence was that all included studies, except 1, were observational cohorts. Moreover, very few studies were designed purposively to examine the effect of unhealthy food and beverage consumption on indicators of overweight/obesity. Although RCTs could provide greater certainty of evidence, purposeful consumption of unhealthy foods and beverages is precluded for ethical reasons. The results from meta-analyses were limited by the small number of studies that could be harmonized. Further, the pooled studies included different baseline ages and varying duration of follow-up across studies, hence pooled estimates should be interpreted with caution. The high heterogeneity of reporting of dietary intakes [i.e., differences in dietary assessment methods, recall periods, units of measurements, and definition of the exposure (typology of food item/food group)] prevented further data harmonization and limited the extent of meta-analyses.

### Strengths and limitations of the review

Strengths of the review are the inclusion of studies dating from 1993, with no restrictions on language or country. Other systematic reviews of unhealthy food consumption in infants and young children have been confined to countries classified as high on the Human Development Index and English language only (13, 135). The inclusion of infants and young children in this review also added valuable insights because existing reviews have predominantly examined later childhood and adolescence (e.g., reference 14). We followed Cochrane recommended methods for RoB and grading of evidence (25). We used a comprehensive food-based and nutrient-based approach in addition to the NOVA classification, to consider all types of unhealthy foods and beverages. We searched 3 databases and did not search gray literature, which could be a potential limitation of the review.

### Recommendations for future research

Studying the effect of unhealthy food consumption on the risk of overweight/obesity is challenging due to the high heterogeneity in measuring and reporting dietary intakes. More robust nutritional epidemiological intervention or prospective studies are needed to enhance our understanding of the relation between unhealthy food and beverage consumption and overweight/obesity. Evidence could be strengthened by collecting the highest quality dietary data possible and by standardizing data collection and reporting measures of diet in studies investigating the relation between unhealthy food consumption and health. A clear definition and conceptualization of the dietary risk factors for overweight/obesity and nutrition-related NCDs is key to ensuring standardization and hence comparison of exposure measures across studies. Dietary assessment approaches are now recognizing the importance of capturing information on unhealthy food and beverage intake, as reflected by the updated infant and young child feeding indicators (58), which include sentinel unhealthy foods and SSBs. Furthermore, the recently published diet quality questionnaire (internationally standardized survey instrument) (136) provides a list of food groups to limit or avoid (i.e., baked sweets; other sweets; sodas, energy drinks, sports drinks; fruit juice and fruit-flavored drinks; sweet tea, coffee, cocoa; packaged ultraprocessed salty snacks; instant noodles; deep-fried foods; fast foods). These food group classifications could be applied in nutritional epidemiological studies. Diet quality questionnaires aligned with the WHO and UNICEF indicators for infants and young children are soon to be released. Wider adoption of the STROBE-nut reporting guidelines in future studies would help enhance evidence syntheses (137). In addition, future work should focus on children ≤2 y in LMIC settings where diets are rapidly changing, and multiple forms of malnutrition coexist.

## Conclusion

In children ≤10.9 y, consumption of SSBs and unhealthy foods may increase BMI, percentage body fat, or odds of overweight/obesity (low to very low certainty). Consumption of ASBs and 100% fruit juice makes little or no difference to BMI, percentage body fat, or overweight/obesity outcomes (low certainty). High-quality nutritional epidemiological studies that are designed to assess the effects of unhealthy food consumption during childhood on risk of overweight/obesity are needed to contribute to a more robust evidence base upon which to develop policy recommendations. This is key to address the growing burden of overweight and obesity that children are experiencing worldwide. Evidence from low-income countries is also needed.

## Supplementary Material

nmac032_Supplemental_File

## Notes

Funding support was received from the Food and Nutrition Action in Health Systems unit, Department of Nutrition and Food Safety, WHO (EKR, RP, PG). The funders had no role in the collection, analysis, interpretation of data, or writing of the report. ESP was supported by the NIHR Leicester Biomedical Research Centre.

Author disclosures: The authors report no conflicts of interest.

Supplemental Methods 1–4, Supplemental Tables 1–11, and Supplemental Figures 1 and 2 are available from the “Supplementary data” link in the online posting of the article and from the same link in the online table of contents at https://academic.oup.com/advances/.

Abbreviations used: aOR, adjusted odds ratio; aRR, adjusted relative risk; ASB, artificially sweetened beverage; GRADE, Grading of Recommendations Assessment, Development, and Evaluation; HIC, high-income countries; LMIC, low- and middle-income countries; MIC, middle-income countries; NCD, noncommunicable disease; NRSI, nonrandomized study of interventions; PI/ECO, population, intervention or exposure, comparator, outcome; RCT, randomized controlled trial; RoB, risk of bias; ROBINS, risk of bias in nonrandomized studies of interventions; SSB, sugar-sweetened beverage; UPF, ultraprocessed food.
