# Corrections

**DOI:** 10.1093/advances/nmac069

**Published:** 2022-10-02

Correction to: E K Rousham, S Goudet, O Markey, P Griffiths, B Boxer, C Carroll, E S Petherick, R Pradeilles, Unhealthy Food and Beverage Consumption in Children and Risk of Overweight and Obesity: A Systematic Review and Meta-Analysis,  Adv Nutr 2022;nmac032,  https://doi.org/10.1093/advances/nmac032

In the originally published version of this manuscript, Legends for Figures 3, 4 and 5 omitted the unit of consumption of beverages. For all plots, the summary point estimate represented a unit change of the outcome “per 250 mL serving” of the beverage.

This error has been corrected.

## Notes

Address correspondence to EKR (e-mail: e.k.rousham@lboro.ac.uk)

