# Supplementary material for: Unhealthy Food and Beverage Consumption in Children and Risk of Overweight and Obesity: A Systematic Review and Meta-Analysis
Source: Adv Nutr. 2022 Apr 1;13(5):1669–96. doi: 10.1093/advances/nmac032 (PMC9526862; doi:10.1093/advances/nmac032)
Supplement: nmac032_Supplemental_File [file nmac032_supplemental_file.docx]

**Unhealthy food and beverage consumption in children and risk of overweight and obesity: a systematic review and meta-analysis**

Rousham et al.

Online Supplementary Material

**Supplemental Method 1** Classification of foods and beverages as unhealthy for the review of effects of unhealthy food and beverage consumption among children ≤10.9 y on risk of overweight and obesity

The *first classification* used was the NOVA classification system (1) which categorizes foods and beverages based on the nature, extent and purpose of industrial processing (i.e. the physical, biological and chemical processes) that food items and beverages undergo. The NOVA classification includes four groups (2): i. unprocessed or minimally processed foods, ii. processed culinary ingredients, iii. processed foods (i.e., food manufactured with the addition of salt or sugar to unprocessed or minimally processed foods (canned foods or breads/cheeses) and iv. ultra-processed foods. Of interest to this review is the fourth category, ultra-processed foods which are listed in Supplemental Table 1, category A. These products are energy-dense and characterized by high content of free sugar, total/saturated/*trans* fats or sodium and low content of protein and fibers (3,4) and are known to be harmful to human health (5). Formula and follow-on milk were excluded from the NOVA classification list as the effects of type of milk feeding during the complementary feeding period was examined in a separately commissioned systematic review.

The *second classification* used were the unhealthy indicators from the WHO guide to assess infant and young child feeding practices (6) (see Supplemental Table 1, category B), namely: i. sweet beverages (i.e. commercially produced and packaged sweetened drinks, 100% fruit juice drinks and home-made drinks with sweeteners added) and ii. sentinel unhealthy foods (i.e. sweet foods and fried/salty foods).

The *third and fourth categories* were based on the nutrient content of foods and beverages (See Supplemental Table 1, categories C and D). The nutrients of interest were saturated and trans fats and free sugars due to their known association with diet-related NCDs. Free sugars included “all added sugars in any form; all sugars naturally present in fruit and vegetable juices, purées and pastes and similar products in which the structure has been broken down; all sugars in drinks (except for dairy-based drinks); and lactose and galactose added as ingredients (7). Sugars naturally present in “milk and dairy products, fresh and most types of processed fruit and vegetables and in cereal grains, nuts and seeds” (7) were not included in the definition of free sugars. The basis for including all the sugars in drinks in the definition is that drinks are consumed in larger quantities and have therefore the potential to provide high amounts of sugar. In addition, they are known to have a lower satiety effect in comparison to solid foods (7).

**Supplemental Method 2** Harmonization process for the meta-analysis on the effects of consumption of sugar-sweetened beverages among children ≤10.9 y on risk of overweight and obesity

To harmonize data, we made all serving sizes equivalent so that the summary point estimate represented a unit change in daily serving of SSB, defined as 250 mL for standardization. Serving sizes were reported in studies as 12 oz (approx. 354 mL) for sodas (8), 8 oz (236.5 mL) for juice (8,9); three-quarters of a cup (184 mL) for juice (10); per 200 mL glass (11); per 100 mL (12); or per oz/day (per 29.6 mL/day) (13). If the serving size was not reported, we imputed a value of 250 mL per serving. Studies were excluded from meta-analyses if the intervention was reported as frequency of consumption where no portion or serving size was indicated, or as categories of consumption as these were not necessarily linear (14,15). Given the variability across studies in measurement of dietary intakes in studies (different recall periods, self-report versus weighed intakes, single vs repeated assessments) and the range of participant ages included in studies we avoided making further assumptions around linearity. Meta-regression, which could potentially have been used to convert categories of consumption to linear portions, is not recommended for fewer than 10 studies (16).

**Supplemental Method 3** Harmonization process for the meta-analysis on the effects of consumption of unhealthy foods among children ≤10.9 y on risk of overweight and obesity

Three studies reported odds ratios of overweight/obesity with unhealthy food consumption: Zulfiqar et al., (17) reported a dichotomous frequency of consumption with no indication of portion size; Wijga et al., (18) reported frequency of intake (continuous) without portion size, and Bel-Serrat et al., (19) reported no portion size. Several other studies reported frequencies of intake without portions or servings indicated (8,20,21). Similarly, with continuous outcomes (BMI, BMI z-scores), there were no two studies reporting intake as either portions/servings or g/day. Three studies (four articles) reported intake of foods as a percentage of total energy intake for an individual (two studies assessed ultra-processed food intake, but with different anthropometric outcomes; one study assessed added sugar intake) (22–25). For these studies, percent energy intakes could not be converted to g/day because the denominator was the energy intake of the individual, not the mean energy intake of the sample (22–25).

**Supplemental Method 4** Grading of the evidence for the effects of unhealthy food and beverage consumption among children ≤10.9 y on risk of overweight and obesity

Within the grade profile for each outcome, we assessed the five domains namely: risk of bias; inconsistency; imprecision; indirectness and publication bias. Each domain was assessed as not serious, serious or very serious except risk of bias which had a fourth level of extremely serious when used in conjunction with ROBINS-I. As the risk of bias for all observational studies was assessed using ROBINS-I, studies were initially graded as high certainty of evidence in accordance with Cochrane guidance (16). The certainty of evidence was downrated by two levels if there was evidence of risk of bias due to non-randomization, namely due to the likelihood of confounding and selection bias. Specific guidance on the use of GRADE and ROBINS-I was followed for all evidence profiles (26). Directness was assessed based on whether studies addressed the review question in relation to similarity of populations, interventions (exposures) and comparators, using the PICO of this review. In general, this was not downrated because we assessed that studies had addressed the PICO of the review with the exception that no studies had been conducted in low income countries. For the assessment of inconsistency, we were not able to assess point estimates, overlap in confidence intervals or heterogeneity statistics, such as *I*^2^ (27) because the data from all studies could not be meta-analysed. Therefore, we did not downrate evidence for inconsistency, but noted that the interventions and comparators were different across studies. The rating of imprecision was assessed by considering the effect estimates and confidence intervals, or event rate for dichotomous outcomes or number of participants. We also referred to sample size calculations from included studies where available to support decisions where meta-analysis had not been conducted. Only two studies provided sample size estimates for critical outcomes. One study estimated a sample of n=42 required to assess the effect of consuming 10% of total energy from free sugars at 12 months on differences in weight status at 30 months (28). Another study estimated a sample of n=670 to detect a 5% different in rates of overweight/obesity by quartiles of exposure for fast food intake, with 90% power (29).

**Supplementary Tables**

**Supplemental Table 1** Classification used to define foods and beverages as unhealthy for the review of effects of unhealthy food and beverage consumption among children ≤10.9 y on risk of overweight and obesity

| **A. Unhealthy foods and beverages (ultra-processed foods) defined as per the NOVA classification system †** |
| --- |
| Sugar-sweetened beverages (sweetened fruit and vegetable juices, soft drinks, fruit and vegetable concentrates, fruit-flavored drinks, fruit and vegetable smoothies, nectars, chocolate/cocoa drinks, milk/yoghurt drinks, energy drinks, sweetened/flavored water). These refer to packaged/commercially produced drinks. |
| Diet or light soft drinks (with non-caloric or artificial sweeteners) |
| Fruit/flavored/sweetened yoghurts |
| Chocolate |
| Candies/sweets |
| Ice cream |
| Sweet packaged snacks (e.g., sweetened popcorn, caramelized nuts) |
| Savory packaged snacks (e.g., crisps, salted popcorn, cheese puffs) |
| Margarine and other spreads |
| Biscuits |
| Pastries (e.g. croissant, pain au chocolat, brioche, doughnuts) |
| Energy bars |
| Cakes |
| Sweetened breakfast cereals |
| Instant noodles |
| Pizza |
| Pies |
| Processed meat or reconstituted meat products (e.g. sausages, ham, hot dogs, fried/battered chicken, poultry nuggets) and fish nuggets/battered fish |
| **B. Unhealthy foods and beverages items defined in the WHO-UNICEF sentinel unhealthy food categorization*** (including only those items not already listed under A) |
| Fried potatoes/chips |
| 100% fruit juices (i.e. unsweetened) whether made at home, by informal food vendors or packaged in cans, bottles, boxes, sachets and other sweet beverages that are home-made and to which any kind of sweeteners (e.g. sugar, honey, syrup, flavored powders) have been added. |
| **C. Unhealthy items defined as high in saturated fat content** (including only those items not already listed under A or B) |
| Butter, lard, ghee |
| **D. Unhealthy items defined as high in free sugar content‡** (including only those items not already listed under A, B or C) |
| Table sugar |
| Jam, honey, syrups |
| Unsweetened, 100% fruit and vegetable juices, concentrates and smoothies |
| **E. Other included terminologies used by study authors to refer to unhealthy items** |
| Non-core food; extra food; convenience foods; junk food; fast food; snack foods |

† NOVA classification based on Monteiro et al. 2010 (1).

*based on WHO and UNICEF, 2021 (6).

‡ based on Swan et al. 2018 (7).

**Supplemental Table 2** Database searches for the effects of unhealthy food and beverage consumption among children ≤10.9 y on risk of overweight and obesity

**Search Name: WHO Cochrane searches**

Date Run: 17/12/2020 18:18:07

ID Search Hits

#1 (infant):ti,ab,kw 49857

#2 (infants):ti,ab,kw 32907

#3 (infancy):ti,ab,kw 3763

#4 MeSH descriptor: [Infant] this term only 21560

#5 (toddler*):ti,ab,kw 1748

#6 (baby):ti,ab,kw 5083

#7 (babies):ti,ab,kw 4875

#8 MeSH descriptor: [Child] this term only 48734

#9 ("Child"):ti,ab,kw 146976

#10 (school child*):ti,ab,kw 20996

#11 (boy):ti,ab,kw 711

#12 (boys):ti,ab,kw 6378

#13 (girl):ti,ab,kw 1470

#14 (girls):ti,ab,kw 7051

#15 ("pre-school*"):ti,ab,kw 614

#16 ("kindergar*"):ti,ab,kw 0

#17 ("elementary school"):ti,ab,kw 1021

#18 (primary school):ti,ab,kw 9832

#19 #1 OR # 2 OR #5 OR #6 OR #7 OR #8 OR #9 OR #10 OR #11 OR #13 OR #15 OR #16 OR #17 OR #18 1068829

#20 MeSH descriptor: [Snacks] explode all trees 282

#21 (snack):ti,ab,kw 1519

#22 #20 OR #21 1611

#23 MeSH descriptor: [Candy] explode all trees 792

#24 (candy):ti,ab,kw 369

#25 (candies):ti,ab,kw 64

#26 (sweets):ti,ab,kw 293

#27 (confection*):ti,ab,kw 103

#28 (sweet food):ti,ab,kw 525

#29 MeSH descriptor: [Chocolate] explode all trees 60

#30 (chocolat*):ti,ab,kw 1077

#31 (salt*):ti,ab,kw 7071

#32 (salty food):ti,ab,kw 125

#33 (savoury):ti,ab,kw 158

#34 #23 OR #24 OR #25 OR #26 OR #27 OR #28 OR #29 OR #30 OR #31 OR #32 OR #33 9795

#35 MeSH descriptor: [Fast Foods] explode all trees 105

#36 ("fast-food"):ti,ab,kw 601

#37 (street food):ti,ab,kw 29

#38 (junk food*):ti,ab,kw 77

#39 (convenience food*):ti,ab,kw 264

#40 (ready-prepared food*):ti,ab,kw 2

#41 (ready to eat meal*):ti,ab,kw 58

#42 (takeaway food*):ti,ab,kw 23

#43 (take-away food*):ti,ab,kw 17

#44 (takeout food*):ti,ab,kw 5

#45 (take-out food*):ti,ab,kw 8

#46 (fried food*):ti,ab,kw 158

#47 (ultra-processed food*):ti,ab,kw 32

#48 (ultraprocessed food*):ti,ab,kw 36

#49 (processed food*):ti,ab,kw 650

#50 (processed meat):ti,ab,kw 205

#51 (fatty food*):ti,ab,kw 3259

#52 #35 OR #36 OR #37 OR #38 OR #39 OR #40 OR #41 OR #42 OR #43 OR #44 OR #45 OR #46 OR #47 OR #48 OR #49 OR #50 OR #51 4984

#53 MeSH descriptor: [Dietary Sucrose] explode all trees 305

#54 (sugar):ti,ab,kw 8085

#55 (sugars):ti,ab,kw 961

#56 (sugary):ti,ab,kw 213

#57 MeSH descriptor: [Sweetening Agents] explode all trees 736

#58 (sweetener*):ti,ab,kw 550

#59 #53 OR #54 OR #55 OR #56 OR #57 OR #58 9425

#60 ("unhealthy"):ti,ab,kw 1513

#61 (inappropriate food*):ti,ab,kw 103

#62 (non-nutritive):ti,ab,kw 300

#63 (nonnutritive):ti,ab,kw 383

#64 (nutrient poor):ti,ab,kw 301

#65 (energy dense food*):ti,ab,kw 463

#66 (less healthy meal*):ti,ab,kw 941

#67 (less healthy food*):ti,ab,kw 1705

#68 (low nutrient):ti,ab,kw 1512

#69 (nutritive value):ti,ab,kw 496

#70 (energy-dense):ti,ab,kw 436

#71 #60 OR #61 OR #62 OR #63 OR #64 OR #65 OR #66 OR #67 OR #68 OR #69 OR #70 6356

#72 (complementary food*):ti,ab,kw 808

#73 (complementary diet):ti,ab,kw 644

#74 (complementary meal*):ti,ab,kw 191

#75 #72 OR #73 OR #74 1175

#76 MeSH descriptor: [Beverages] explode all trees 6029

#77 (beverage*):ti,ab,kw 6631

#78 (soda):ti,ab,kw 413

#79 (sodas):ti,ab,kw 47

#80 (carbonated drink):ti,ab,kw 147

#81 (sweet drink*):ti,ab,kw 172

#82 (sweetened drink*):ti,ab,kw 407

#83 (soft drink*):ti,ab,kw 559

#84 #76 OR #77 OR #78 OR #79 OR #80 OR #81 OR #82 OR #83 11157

#85 #22 OR #34 OR #52 OR #59 OR #71 OR #75 OR #84 37956

#86 ("intake"):ti,ab,kw 54242

#87 (intakes):ti,ab,kw 4651

#88 (consum*):ti,ab,kw 70753

#89 (feeding):ti,ab,kw 22133

#90 (eating):ti,ab,kw 14676

#91 (drinking):ti,ab,kw 11635

#92 (eat):ti,ab,kw 3672

#93 (drink):ti,ab,kw 6818

#94 #86 OR #87 OR #88 OR #89 OR #90 OR #91 OR #92 OR #93 139621

#95 MeSH descriptor: [Animals] this term only 9792

#96 MeSH descriptor: [Humans] this term only 591274

#97 (#19 AND #85 and #94) NOT (#95 NOT #96) 16629

#98 ("randomized-controlled trial"):pt 498138

#99 (controlled clinical trial):pt 323841

#100 (randomized):ti,ab,kw 901454

#101 (trial):ti,ab,kw 825752

#102 (groups):ti,ab,kw 475952

#103 (comparative study):pt 166014

#104 MeSH descriptor: [Control Groups] explode all trees 111

#105 MeSH descriptor: [Follow-Up Studies] explode all trees 59090

#106 (follow-up stud*):ti,ab,kw 219260

#107 (follow-up assessment):ti,ab,kw 58334

#108 MeSH descriptor: [Prospective Studies] explode all trees 91786

#109 (prospective stud*):ti,ab,kw 206021

#110 MeSH descriptor: [Evaluation Studies as Topic] explode all trees 50832

#111 (evaluat*):ti,ab,kw 492538

#112 (quasi experiment*):ti,ab,kw 5115

#113 (quasiexperiment*):ti,ab,kw 4452

#114 MeSH descriptor: [Interrupted Time Series Analysis] explode all trees 51

#115 (ITS stud*):ti,ab,kw 108192

#116 (time series):ti,ab,kw 7967

#117 (time point*):ti,ab,kw 69107

#118 MeSH descriptor: [Controlled Before-After Studies] explode all trees 71

#119 (controlled):ti,ab,kw 744775

#120 (CBA stud*):ti,ab,kw 267

#121 (pre test):ti,ab,kw 26376

#122 (pretest):ti,ab,kw 10268

#123 (post test):ti,ab,kw 41465

#124 (posttest):ti,ab,kw 11946

#125 (pre intervention):ti,ab,kw 36566

#126 (post intervention):ti,ab,kw 61277

#127 (before-after stud*):ti,ab,kw 1459

#128 ("before and after"):ti,ab,kw 59238

#129 (nonrandom):ti,ab,kw 283

#130 (non-random*):ti,ab,kw 5626

#131 MeSH descriptor: [Cohort Studies] explode all trees 148452

#132 (cohort stud*):ti,ab,kw 49549

#133 (longitudinal stud*):ti,ab,kw 18887

#134 #98 OR #99 OR #100 OR #101 OR #102 OR #103 OR #104 OR #105 OR #106 OR #107 OR #108 OR #109 OR #110 OR #111 OR #112 OR #113 OR #114 OR #115 OR #116 OR #117 OR #118 OR #119 OR #120 OR #121 OR #122 OR #123 OR #124 OR #125 OR #126 OR #127 OR #128 OR #129 OR #130 OR #131 OR #132 OR #133 1418750

#135 #97 AND #134 with Publication Year from 1971 to 2020, with Cochrane Library publication date Between Jan 1971 and Dec 2020, in Trials 15393

#136 ("editorial"):pt 2752

#137 (comment):pt 1882

#138 (news):pt 334

#139 ("Letter"):pt 12061

#140 (review):pt 17935

#141 ("systematic review"):pt 59

#142 ("meta-analysis"):pt 530

#143 ("meta-analysis"):ti,ab,kw 17978

#144 ("meta analyses"):ti,ab,kw 17978

#145 (retracted publication):pt 485

#146 (retraction of publication):pt 58

#147 (retraction of publication):ti,ab,kw 42

#148 (retraction of publication):pt 58

#149 #136 OR #137 OR #138 OR #139 OR #140 OR #141 OR #142 OR #143 OR #144 OR #145 OR #146 OR #147 OR #148 48859

#150 #135 NOT #149 with Cochrane Library publication date Between Jan 1971 and Dec 2020 15224

**Database: EMBASE**

**Date: 18 December 2020**

| Search | Query | Results |
| --- | --- | --- |
| #12 | #10 not #11 | 6982 |
| #11 | Limit #10 to conference abstracts | 2414 |
| #10 | #8 NOT #9 | 9396 |
| #9 | Search: editorial.pt OR comment.pt OR news.pt OR letter.pt OR review.pt OR "systematic review".pt OR "systematic review".tw OR "meta-analysis".pt OR "meta-analysis".tw OR "meta-analyses".tw OR "retracted publication".pt OR "retraction of publication".pt OR "retraction of publication".tw OR "retraction notice".tw | 4726267 |
| #8 | Search: #6 and #7 | 10108 |
| #7 | Search: randomized controlled trial.pt OR controlled clinical trial.pt OR randomized.tw OR trial.tw OR groups.tw OR comparative study.pt OR control groups.mp OR follow-up studies.mp OR follow-up stud*.tw OR follow-up assessment.tw OR prospective studies.mp OR prospective stud*.tw OR "evaluation studies as topic".mp OR evaluat*.tw OR quasi experiment*.tw OR quasiexperiment*.tw OR interrupted time series analysis.mp OR ITS stud*.tw OR time series.tw OR time point*.tw OR controlled before-after studies.mp OR controlled.tw OR CBA stud*.tw OR pre test.tw OR pretest.tw OR post test.tw OR posttest.tw OR pre intervention.tw OR post intervention.tw OR before-after stud*.tw OR "before and after".tw OR nonrandom*.tw OR non-random*.tw OR cohort studies.mp OR cohort stud*.tw OR longitudinal stud*.tw | 9040729 |
| #6 | Search: #4 NOT #5 | 21571 |
| #5 | Search: (animals.mp NOT humans.mp) | 767206 |
| #4 | 1 and 2 and 3 | 21724 |
| #3 | Search: intake.tw OR intakes.tw OR consum*.tw OR feeding.tw OR eating.tw OR drinking.tw OR eat.tw OR drink.tw | 1281552 |
| #2 | Search: beverages.mp OR beverage*.tw OR soda.tw OR sodas.tw OR carbonated drink*.tw OR sweet drink*.tw OR sweetened drink*.tw OR soft drink*.tw OR complementary food*.tw OR complementary diet.tw OR complementary meal*.tw OR unhealthy.tw OR inappropriate food.tw OR inappropriate foods.tw OR non-nutritive.tw OR nonnutritive.tw OR nutrient poor.tw OR less healthy meal*.tw OR less healthy food*.tw OR low nutrient.tw OR nutritive value.tw OR energy-dense.tw OR dietary sucrose.mp OR sugar.tw OR sugars.tw OR sugary.tw OR sweetening agents.mp OR sweetener.tw OR sweeteners.tw OR fast foods.mp OR fast food*.tw OR street food*.tw OR junk food*.tw OR convenience food*.tw OR ready-prepared food*.tw OR ready to eat meal*.tw OR takeaway food*.tw OR take-away food*.tw OR takeout food*.tw OR take-out food*.tw OR fried food*.tw OR ultra-processed food*.tw OR ultraprocessed food*.tw OR processed food*.tw OR processed meat*.tw OR fatty food*.tw OR candy.mp OR candy.tw OR candies.tw OR sweets.tw OR confection*.tw OR sweet food*.tw OR chocolate.mp OR chocolat*.tw OR salt*.tw OR savoury.tw OR Snacks.mp OR snack*.tw | 450238 |
| #1 | Search: infant.mp OR infant.tw OR infants.tw OR infancy.tw OR toddler*.tw OR baby.tw OR babies.tw OR child.mp OR child*.tw OR schoolchild*.tw OR boy.tw OR boys.tw OR girl.tw OR girls.tw OR pre school*.tw OR kindergar*.tw OR elementary school*.tw OR primary school*.tw | 3169372 |

**Database: PubMed**

**Date:16 December 2020**

| Search | Query | Results |
| --- | --- | --- |
| #16 | #14 NOT #15 | 13,227 |
| #15 | Search: editorial[Publication Type] OR comment[Publication Type] OR news[Publication Type] OR letter[Publication Type] OR review[Publication Type] OR "systematic review"[Publication Type] OR "systematic review"[tiab] OR "meta-analysis"[Publication Type] OR "meta-analysis"[tiab] OR "meta-analyses"[tiab] OR "retracted publication"[Publication Type] OR "retraction of publication"[Publication Type] OR "retraction of publication"[tiab] OR "retraction notice"[tiab] Filters: from 1971/1/1 - 2020/11/27 | 4,922,701 |
| #14 | Search: #13 AND #14 Filters: from 1971/1/1 - 2020/11/30 Sort by: Publication Date | 14,355 |
| #13 | Search: #11 AND #12 | 14,380 |
| #12 | Search: randomized controlled trial[pt] OR controlled clinical trial[pt] OR randomized[tiab] OR trial[tiab] OR groups[tiab] OR comparative study[pt] OR control groups[mh] OR follow-up studies[mh] OR follow-up stud*[tiab] OR follow-up assessment[tiab] OR prospective studies[mh] OR prospective stud*[tiab] OR "evaluation studies as topic"[mh] OR evaluat*[tiab] OR quasi experiment*[tiab] OR quasiexperiment*[tiab] OR interrupted time series analysis[mh] OR ITS stud*[tiab] OR time series[tiab] OR time point*[tiab] OR controlled before-after studies[mh] OR controlled[tiab] OR CBA stud*[tiab] OR pre test[tiab] OR pretest[tiab] OR post test[tiab] OR posttest[tiab] OR pre intervention[tiab] OR post intervention[tiab] OR before-after stud*[tiab] OR "before and after"[tiab] OR nonrandom*[tiab] OR non-random*[tiab] OR cohort studies[mh] OR cohort stud*[tiab] OR longitudinal stud*[tiab] | 9,144,658 |
| #11 | Search: (#1 AND #9 AND #10) NOT (animals[mh] NOT humans[mh]) | 26,811 |
| #10 | Search: intake[tiab] OR intakes[tiab] OR consum*[tiab] OR feeding[tiab] OR eating[tiab] OR drinking[tiab] OR eat[tiab] OR drink[tiab] | 1,008,423 |
| #9 | Search: #2 OR #3 OR #4 OR #5 OR #6 OR #7 OR #8 | 523,824 |
| #8 | Search: beverages[mh] OR beverage*[tiab] OR soda[tiab] OR sodas[tiab] OR carbonated drink*[tiab] OR sweet drink*[tiab] OR sweetened drink*[tiab] OR soft drink*[tiab] | 162,087 |
| #7 | Search: complementary food*[tiab] OR complementary diet[tiab] OR complementary meal*[tiab] | 1486 |
| #6 | Search: unhealthy[tiab] OR inappropriate food[tiab] OR inappropriate foods[tiab] OR non-nutritive[tiab] OR nonnutritive[tiab] OR nutrient poor[tiab] OR less healthy meal*[tiab] OR less healthy food*[tiab] OR low nutrient[tiab] OR nutritive value[tiab] OR energy-dense[tiab] | 23,532 |
| #5 | Search: dietary sucrose[mh] OR sugar[tiab] OR sugars[tiab] OR sugary[tiab] OR sweetening agents[mh] OR sweetener[tiab] OR sweeteners[tiab] | [133,115](https://pubmed.ncbi.nlm.nih.gov/?term=dietary+sucrose%5Bmh%5D+OR+sugar%5Btiab%5D+OR+sugars%5Btiab%5D+OR+sugary%5Btiab%5D+OR+sweetening+agents%5Bmh%5D+OR+sweetener%5Btiab%5D+OR+sweeteners%5Btiab%5D&sort=relevance&size=200&ac=no) |
| #4 | Search: fast foods[mh] OR fast food*[tiab] OR street food*[tiab] OR junk food*[tiab] OR convenience food*[tiab] OR ready-prepared food*[tiab] OR ready to eat meal*[tiab] OR takeaway food*[tiab] OR take-away food*[tiab] OR takeout food*[tiab] OR take-out food*[tiab] OR fried food*[tiab] OR ultra-processed food*[tiab] OR ultraprocessed food*[tiab] OR processed food*[tiab] OR processed meat*[tiab] OR fatty food*[tiab] | 12,955 |
| #3 | Search: candy[mh] OR candy[tiab] OR candies[tiab] OR sweets[tiab] OR confection*[tiab] OR sweet food*[tiab] OR chocolate[mh] OR chocolat*[tiab] OR salt*[tiab] OR savoury[tiab] | 215,735 |
| #2 | Search: Snacks[mh] OR snack*[tiab] | 8,453 |
| #1 | Search: infant[mh] OR infant[tiab] OR infants[tiab] OR infancy[tiab] OR toddler*[tiab] OR baby[tiab] OR babies[tiab] OR child[mh] OR child*[tiab] OR schoolchild*[tiab] OR boy[tiab] OR boys[tiab] OR girl[tiab] OR girls[tiab] OR pre school*[tiab] OR kindergar*[tiab] OR elementary school*[tiab] OR primary school*[tiab] | 3,071,122 |

**Supplemental Table 3** Data extraction form template for the effects of unhealthy food and beverage consumption among children ≤10.9 y on risk of overweight and obesity

| **Study details** | | | | | | | | | | | | | | |
| --- | --- | --- | --- | --- | --- | --- | --- | --- | --- | --- | --- | --- | --- | --- |
| Initials of extractor | | Article of study ID | | | | | Author, Year | | | | | | Reference | |
| Paper title | | | | | | | | | | | | | | |
| Study aim | | | | | | | | | | | | | | |
| **Study design details** | | | | | | | | | | | | | | |
| Study design | | | | | Methodology | | | | | | | Recruitment | | |
| Funding source | | | | | | | | Authors’ declaration of interests | | | | | | |
| **Setting** | | | | | | | | | | | | | | |
| Country | | | Setting (Rural/urban) | | | | | | Country income level (low, middle, high) | | | | | |
| Recruitment method | | | | | | Study start – end date | | | | | | | | |
| **Participant characteristics and sample size** | | | | | | | | | | | | | | |
| Age range of sample, baseline (add units mo/y) | Mean age at baseline exposure (add units mo/y) | | | | | SD/IQR/SE (specify) Baseline age | | | Mean age at follow-up exposure (include units mo/y) | | | | | SD/IQR/SE (specify) follow-up age |
| Length of follow-up (mo/y) (state whether this is mean, median or range of follow-up period) | Sample size at baseline. Specify total sample and (exposed n and non-exposed n) | | | | |  | | |  | | | | |  |
| **Exposure measures** | | | | | | | | | | | | | | |
| Exposure status | Exposure | | | | | Method of assessment | | | | | Quantity/frequency of food or beverage consumed (units) | | |  |
| **Outcome measures and results** | | | | | | | | | | | | | | |
| Outcome (units) | Method of assessment | | | | | Reference/standard/cut-offs used | | | | | Analytical sample (n) | | |  |
| Mean values at baseline (± SD); or mean % prevalence at baseline (95% CIs) | Mean values at endline/follow-up (± SD); or mean % prevalence at endline/follow-up (95% CIs) | | | | | Change in mean value baseline to follow-up/endline (± SE/SD) or change in % prevalence from baseline to follow-up (95% CI) | | | | | Estimates (state whether Beta coeff, OR, RR, other) | | | 95% CI for OR, RR; SE for Beta values or mean difference exposed v non-exposed |
| p-value | Adjusted values? (Y/N) | | | | | Confounders (list all) | | | | | Text summarizing main result for exposure and outcome | | |  |
| **Additional information** | | | | | | | | | | | | | | |
| Authors' conclusion | | | | Extractor study strengths and limitations | | | | | | Other notes (include relevant references) | | | | |

**Supplemental Table 4** Overall risk of bias criteria for non-randomized studies of interventions reporting the effects of unhealthy food and beverage consumption among children ≤10.9 y on risk of overweight and obesity

| **Overall risk of bias assessment for ROBINS-I*** | **Criteria** |
| --- | --- |
| Low | Study is judged to be at low risk of bias for all domains |
| Moderate | Study is judged to be a low or moderate risk of bias for all domains |
| Serious | Study is judged to be a serious risk of bias in at least one domain-but not at critical risk of bias in any domain |
| Critical | The study is judged to be at critical risk of bias in at least one domain |
| No information | No indication that the study is a serious or critical risk of bias *and* there is a lack of information in one or more key domains of bias |

* From Sterne et al 2016 (30).

**Supplemental Table 5** Overall risk of bias criteria for randomized controlled trials reporting the effects of unhealthy food and beverage consumption among children ≤10.9 y on risk of overweight and obesity

| **Overall risk-of-bias judgement*** | **Criteria** |
| --- | --- |
| Low risk of bias | The trial is judged to be at low risk of bias for all domains for this result. |
| Some concerns | The trial is judged to raise some concerns in at least one domain for this result, but not to be at high risk of bias for any domain. |
| High risk of bias | The trial is judged to be at high risk of bias in at least one domain for this result.  Or  The trial is judged to have some concerns for multiple domains in a way that substantially lowers confidence in the result. |
| No information | Lack of information in one or more domains or no clear evidence of serious/critical risk of bias. |

*From Higgins et al., 2019 (16).

**Supplemental Table 6** Quality of evidence GRADE definitions used to assess the certainty of evidence for the effects of unhealthy food and beverage consumption among children ≤10.9 y on risk of overweight and obesity

| GRADE | Definition |
| --- | --- |
| High | We are very confident that the true effect lies close to that of the estimate of the effect. |
| Moderate | We are moderately confident in the effect estimate: The true effect is likely to be close to the estimate of the effect, but there is a possibility that it is substantially different |
| Low | Our confidence in the effect estimate is limited: The true effect may be substantially different from the estimate of the effect. |
| Very Low | We have very little confidence in the effect estimate: The true effect is likely to be substantially different from the estimate of effect |

Source: Schünemann et al., 2013 (27).

**Supplemental Table 7** Characteristics of included studies and reports of studies of the effects of unhealthy food and beverage consumption among children ≤10.9 y and risk of overweight and obesity where data could not be extracted due to aggregate age range^1^

| **Study ID** | **Reference** | **Country** | **Setting (R/U)** | **Income level^2^** | **Recruitment method** |
| --- | --- | --- | --- | --- | --- |
| **Growth and body composition** | |  |  |  |  |
| *Studies not extracted* | | | | | |
| Berkey 2004 | Berkey 2004 (31) | USA | Both | HIC | Mail |
| Bisset 2007 | Bisset 2007 (32) | Canada | Both | HIC | School |
| Field 2004 | Field 2004 (33) | USA | NS | HIC | Mail |
| Jensen 2013 (2) | Jensen 2013b (34) | Australia | NS | HIC | School |
| Johnson 2012 | Johnson 2012 (35) | Australia | R | HIC | School |
| Lee 2018 | Lee 2018 (36) | South Korea | U | HIC | School |
| Mrdjenovic 2003 | Mrdjenovic 2003 (37) | USA | U | HIC | Summer day camp |
| Mundt 2006 | Mundt 2006 (38) | Canada | U | HIC | School |
| Nissinen 2009 | Nissinen 2009 (39) | Finland | Both | HIC | Clinic |
| Phillips 2004 | Phillips 2004 (40) | USA | U | HIC | Schools, summer camps, friends, and family |
| Seferidi 2018 | Seferidi 2018 (41) | UK | Both | HIC | NS |
| Shroff 2014 | Shroff 2014 (42) | Colombia | U | MIC | School |
| Xue 2016 | Xue 2016 (43) | China | NS | MIC | Household random cluster sampling |
| *Reports of studies not extracted* | | | | | |
| Cowin 2001 | Dong 2015 (44) | UK | NS | HIC | Clinic |
| Libuda 2008 | Alexy 2011 (45) | Germany | U | HIC | Contacts, maternity wards, and clinics |
|  | Libuda 2008 (46) | Germany | U | HIC |  |

^1^HIC, high-income country; MIC, middle-income country; NS, not stated; R, rural; RCT, randomized controlled trial; U, urban; y, year.

^2^Calculated using the World Bank Atlas method for the 2021 fiscal year (based on gross national income per capita in 2019) (47).

**Supplemental Table 8** Characteristics of included studies of the effect of unhealthy food and beverage consumption among children ≤10.9 y on growth and body composition outcomes **^1^**

| **Study ID** | **Reference** | **Country** | **Setting (R/U)** | **Income level^2^** | **Recruitment method** | **Exposure** | **Baseline age (mean or range)** | | | **Outcome assessed** |  |
| --- | --- | --- | --- | --- | --- | --- | --- | --- | --- | --- | --- |
| **Prospective cohort studies** | | | | | | | | | | | |
| Alviso-Orellana 2018 | Alviso-Orellana 2018 (48) | Peru | NS | MIC | Home | Snacks-salty and fatty foods; SSB | 8 y | | | BMI change; WC |  |
| Arcan 2013 | Arcan 2013 (49) | US | R | HIC | School | 100% fruit juice | 5.8 y | | | BMI |  |
| Bayer 2014 | Bayer 2014 (50) | Germany | Both | HIC | School | High-caloric drinks; Energy dense sweets | 6.0 y | | | BMI |  |
| Bel-Serrat 2019 | Bel-Serrat 2019 (19) | Republic of Ireland | Both | HIC | School | Fast food; Savory snacks | 7.9 y | | | % OW/OB; Mean change in BAZ |  |
| Blum 2005 | Blum 2005 (51) | US | R | HIC | School | 100% juice; Diet soda; SSB | 9.3 y | | | Normal weight/overweight assessed by BAZ |  |
| Budree 2017 | Budree 2017 (52) | South Africa | R | MIC | Clinic | Fruit juice | Birth | | | % OW/OB (BAZ >2) |  |
| Byrne 2018 | Byrne 2018 (53) | Australia | R | HIC | Clinic | Sweet beverages | 24.1 mo | | | BAZ |  |
| Cantoral 2016 | Cantoral 2016 (54) | Mexico | U | MIC | Clinic | SSB | 6 mo | | | % obese; % with abdominal obesity (WC >90th centile) |  |
| Carlson 2012 | Carlson 2012 (8) | US | U | HIC | Phone, flyers, presentations | SSB; 100% fruit or vegetable juice; High-fat foods | 6.7 y | | | BMI; %BF |  |
| Costa 2020 | Costa 2020 (55) | Brazil | U | MIC | Clinic | Ultra-processed foods | 6–11 y | | | Fat Mass Index |  |
| Cowin 2001 | Johnson 2007 (56) | UK | NS | HIC |  | SSB; 100% fruit juice; Low energy drinks | 5.2 y; 7.2 y | | | Change in fat mass (DXA)/serving |  |
| DeBoer 2013 | DeBoer 2013 (57) | USA | NS | HIC | "Complex sampling design" | SSB | 2 y | | | BMI at 4 y and 5 y |  |
| DeCoen 2014 | De Coen 2014 (58) | Belgium | NS | HIC | School | Soft drinks; Sweet and savory snacks | 4.95 y | | | BMI (overweight) |  |
| Dubois 2007 | Dubois 2007 (59) | Canada | U | HIC | Clinic | SSB between meals | 2.5 y | | | OB (BMI >95th Percentile) |  |
| Emond 2020 | Emond 2020 (29) | US | U | HIC | Clinic, childcare center, community and recreation events | Fast food | 3–5 y | | | Change in BMI status (normal to overweight or overweight to obese) |  |
| Faith 2006 | Faith 2006 (10) | US | U | HIC | Families participating in supplementary nutrition programme | Fruit juice | 30.2 mo | | | BMI trajectory |  |
| Feldens 2010 | Costa 2019 (22) | Brazil | U | MIC | Clinic | Biscuits; Breakfast cereal; Powdered chocolate; Processed meat; Savory; Soft drink; Sugary milk beverages; Sweets; Others; Total ultra-processed foods | 4 y | | | BMI; WC; WHtR; SSF |  |
| Fiorito 2009 | Fiorito 2009 (60) | US | NS | HIC | Flyers and newspaper advertisements | Sweetened beverage | 5 y | | | Body fat; BMI |  |
| Flores 2013 | Flores 2013 (61) | US | NS | HIC | NS | Sugary beverages | 9 mo | | | BMI ≥ 99th percentile (severe obesity) |  |
| Garden 2011 | Garden 2011 (62) | Australia | U | HIC | Clinic | Extra foods; Dairy products | 18 mo | | | BMI; WC; WHtR; SSF |  |
|  | Garden 2012 (63) | Australia | U | HIC |  | Extra foods; Dairy products | 18 mo | | | BMI trajectory |  |
|  | Zheng 2015 (64) | Australia | U | HIC |  | 100% fruit juices; Diet drinks; SSB | 8 y | | | BAZ change; %BF |  |
| Guerrero 2016 | Guerrero 2016 (65) | US | NS | HIC | NS | Soda; Juice; Fast food | 9 mo | | | BMI trajectory |  |
| Hasnain 2014 | Hasnain 2014 (66) | US | NS | HIC | NS | Fruit and vegetable juices; SSB; ASB | 3–5 y | | | BMI; %BF |  |
| Hooley 2012 | Millar 2014 (20) | Australia | Both | HIC | Approached at home | SSB; High-fat foods | 4.8 y | | | BAZ |  |
|  | Wheaton 2015 (67) | Australia | Both | HIC | Approached at home | SSB | 4–5 y | | | Stability of weight status |  |
|  | Zulfiqar 2019 (17) | Australia | Both | HIC |  | SSB; High-fat foods | 4.2 y | | | Boys: OW/OB v non-OW/OB; Girls: OW/OB vs non-OW/OB |  |
| Hur 2015 | Hur 2015 (68) | South Korea | U | HIC | School | Beverage sugar; Other sugar (total sugar minus that from fruit, milk, and beverages) | 9.9 y | | | BAZ, body fat |  |
| Huus 2009 | Huus 2009 (69) | Sweden | NS | HIC | NS | SSB; Fried potato/French fries; Sausage; Cream/creme fraiche; chips; Pastries; Chocolate; Candy; Lemonade; Ice-cream | Birth | | | BMI (OW/OB) at 5 y |  |
| Hwang 2020 | Hwang 2020 (70) | South Korea | NS | HIC | Clinic | SSB at 21 mo; SSB at 33 mo; SSB at 45 mo | 5 mo | | | Adiposity rebound |  |
| Ismail 2008 | Lim 2009 (13) | US | U | HIC | Home | All SSB | 6.7 y | | | BMI ≥ 85th percentile |  |
| Jackson 2017 | Jackson 2017 (71) | US | NS | HIC | School | SSB; Fast food | 5.6 y | | | BMI |  |
| Jardi 2019 | Jardi 2019 (28) | Spain | U | HIC | Clinic | Free sugars | 0 mo | | | Weight at 30 mo (excess or non-excess weight) |  |
| Kramer 2004 | Kramer 2004 (72) | Belarus | Both | MIC | Clinic | Juice or other liquids | 1 mo | | | Weight-for-age at 1–3 mo; 3–6 mo; 6–9 mo and 9–12 mo |  |
| Laurson 2008 | Laurson 2008 (73) | US | R | HIC | Community | SSB; Change in SSB consumption | Boys 10.8 y; Girls 10.7 y | | | BMI change |  |
| Leermakers 2015 | Leermakers 2015a (74) | Netherlands | U | HIC | Clinic | Sugar containing beverages | 12.9 mo | | | BMI at 2 y; 3 y; 4 y; 6 y; %BF; Android/gynoid fat ratio |  |
| Libuda 2008 | Alexy 1999 (75) | Germany | U | HIC | Contacts, maternity wards, and clinics | Fruit juice | Boys 3 y; Girls 3 y | | | BMI |  |
|  | Buyken 2008 (23) | Germany | U | HIC |  | Added sugar | 2 y | | | Change in body fat (SSF); Change in BMI |  |
|  | Herbst 2011 (24) | Germany | U | HIC |  | Total added sugars; Added sugar from beverages and sweets; Added sugar from other sources | 1 y | | | Change in body fat (SSF); Change in BMI |  |
| Lissau 1993 | Lissau 1993 (76) | Denmark | U | HIC | School | Sweets/candies | 9–10 y | | | BMI >90th percentile (overweight) |  |
| Macintyre 2018 | Macintyre 2018 (77) | UK | Both | HIC | NS | SSB; ASB | 4–5 y | | | BMI (normal weight vs OW/OB); BMI (non obese vs obese) |  |
| Marshall 2003 | Marshall 2018 (78) | US | NS | HIC | Clinic | Beverages | 2–4.7 y | | | Height |  |
|  | Marshall 2019 (9) | US | NS | HIC |  | 100% juice; SSB | 2–4.7 y | | | BMI |  |
| Moore 2019 | Moore 2019 (79) | US | U | HIC | Clinic | Snack food; Sweets | 3–12 mo | | | Weight-for-length |  |
| Newby 2004 | Newby 2003 (80) | US | Both | HIC | Clinic | 'Fat foods' | Boys 2.9 y; Girls 2.9 y | | | Weight change/y |  |
|  | Newby 2004 (81) | US | Both | HIC |  | Fruit juice only; Juice drinks; Soda; Diet soda | 2.9 y | | | Weight change; BMI change |  |
| Olafsdottir 2014 | Olafsdottir 2014 (82) | Belgium, Cyprus, Estonia, Germany, Hungary, Italy, Spain, and Sweden | Both | HIC | Schools and kindergartens | SSB | 2–<6 y; 6–<10 y | | | % increase in BMI; % increase in WHtR |  |
|  | Russo 2018 (21) | Belgium, Cyprus, Estonia, Germany, Hungary, Italy, Spain, and Sweden | Both | HIC |  | Added sugars to milk and fruit | Boys 4.2 y; Girls 4.2 y; Boys 7.4 y; Girls 7.4 y | | | BMI; WC; SSF; %BF |  |
| Olsen 2012 | Olsen 2012 (83) | Denmark | U | HIC | School | Added sugar; Liquid sucrose; Solid sucrose | Boys 9.7 y; Girls 9.4 y (combined in analysis) | | | BMI; WC |  |
| Pan 2014 | Pan 2014 (14) | US | NS | HIC | Mail | SSB, any from 1-12 mo; SSB mean weekly 10-12 mo | ∼1 mo | | | OB (BMI-for-age ≥ 95th percentile) |  |
| Quah 2019 | Quah 2019 (12) | Singapore | U | HIC | Clinic | SSB | 18 mo | | | BMI; SSF; OW/OB |  |
| Santorelli 2014 | Santorelli 2014 (84) | UK | U | HIC | Clinic | SSB; sweetened first foods | 6 mo | | | BMI-for-age z-score |  |
| Shefferly 2016 | Shefferly 2016 (85) | US | NS | HIC | School | Fruit juice at 2-4 y; Fruit juice at 4-5 y | 2 y | | | Height; Weight; BMI; Overweight BMI; Obese BMI |  |
| Skinner 1999 | Skinner 1999 (86) | US | NS | HIC | Posters, referrals, and birth announcements | 100% fruit juice | 2–2.7 y | | | BMI and ponderal index |  |
|  | Skinner 2001 (87) | US | NS | HIC |  | 100% fruit juice | 27 mo | | | BMI |  |
| Sonneville 2015 | Sonneville 2015 (15) | US | U | HIC | Clinic | 100% fruit juice | 1 y | | | BAZ |  |
| Striegel-Moore 2006 | Striegel-Moore 2006 (88) | US | NS | HIC | School | Diet soda; Regular soda; Fruit juice; Fruit drinks | 9–10 y | | | BMI |  |
| Sugimori 2004 | Sugimori 2004 (89) | Japan | NS | HIC | NS | Juice; Noodles | 3 y | | | BMI status |  |
| Tam 2006 | Tam 2006 (90) | Australia | U | HIC | Clinic | Soft drinks/cordial; Fruit juice/fruit drinks | 7.7 y | | | BMI gains/losses |  |
| Thurber 2017 | Thurber 2017 (91) | Australia | Both | HIC | Directly approached families and snowball | SSB; High-fat foods | 0.5–2 y and 3–5 y; 0.5–2 y; 3–5 y | | | BMI |  |
| Traub 2018 | Traub 2018 (92) | Germany | NS | HIC | School | Soft drinks | 7.08 y | | | BMI >90th age and gender specific percentile; BMI >97th age and gender specific percentile; WHtR |  |
| Vilela 2014 | Durao 2015 (93) | Portugal | U | HIC | Clinic | Energy-dense foods | 2 y | | | BAZ |  |
|  | Vedovato 2020 (25) | Portugal | U | HIC |  | Ultra-processed foods | 4 y | | | BMI at 4 y; BMI at 7 y |  |
| Wan 2020 | Wan 2020 (94) | US | U | HIC | Original cohort members’ descendants | 100% fruit juice | 3–6 y | | | BMI |  |
| Wang 2013 | Wang 2013 (95) | China | U | MIC | Clinic | Sweet drinks | 1 mo | | | % OW/OB |  |
| Weijs 2011 | Weijs 2011 (96) | Netherlands | NS | HIC | Magazine publisher notice | Beverage sugar | 8.7 mo | | | % overweight; BMI SD score |  |
| Wijga 2010 | Wijga 2010 (18) | Netherlands | NS | HIC | Clinic patients | Fast food; Snack; Soft drink | | 3–12 mo | % OW/OB | | |
| Zheng 2014 | Zheng 2014 (97) | Denmark | U | HIC | School | SSB | | 9.6 y | BMI; WC; SSF (4 sites) | | |
| **Pre/post study with a control** | | | | | | | | | | | |
| Jensen 2013 (1) | Jensen 2013a (98) | Denmark | U | HIC | School | Sweet drinks; SSB; Soft drinks only | | 6.7 y | BMI change | | |
| **Retrospective cohort study** | | | | | | | | | | | |
| Welsh 2005 | Welsh 2005 (99) | US | NS | HIC | Clinic | Sweet drinks; Fruit juices only | | 33.8 mo | BMI ≥ 95th percentile in those with BMI <85th percentile at baseline; BMI ≥ 95th percentile in those with BMI 85th-<95th percentile at baseline; BMI ≥ 95th percentile in those with ≥95th percentile at baseline | | |
| **Randomized controlled trial** | | | | | | | | | | | |
| Muckelbauer 2016 | Muckelbauer 2016 (11) | Germany | U | HIC | School | Sugar containing beverages; Soft drinks; Juice | | 8.3 y | Mean change in BMI; % overweight and obesity | | |

^1^ASB, artificially-sweetened beverages; BAZ, BMI-for-age z score; DXA, dual-energy X-ray absorptiometry; HIC, high-income country; MIC, middle-income country; NS, not stated; OW/OB, overweight including obesity; OB, obesity only; %BF, percentage body fat; R, rural; SSB, sugar-sweetened beverages; SSF, sum of skinfolds; U, urban; WC, waist circumference; WHtR, waist-to-height ratio.

^2^Calculated using the World Bank Atlas method for the 2021 fiscal year (based on gross national income per capita in 2019) (47).

**Supplemental Table 9** Synthesis of results from included studies of the effects of unhealthy foods and beverage consumption among children ≤10.9 y on body mass index and overweight and obesity outcomes^1^

| **Reference** | **Baseline age (mean or range)** | **Follow-up duration** | **n^2^** | **DAT** | **Exposure** | **Intake unit** | **Comparator** | **Outcomes** | **Estimate^3^** | **Overall RoB** |
| --- | --- | --- | --- | --- | --- | --- | --- | --- | --- | --- |
| **SSB** | | | | | | | | | | |
| 0–<2 y | | | | | | | | | | |
| Cantoral 2016 (54) | 12 mo | 8–14 y | 227 | 3-mo FFQ | SSB | Cumulative consumption in pre-school y | Third vs. first tertile SSB cumulative consumption | Obesity (%) (>2SD BMI z score, WHO 2006) (100) | OR = 2.99, 95% CI: 1.27, 7.00. | Serious |
| Flores 2013 (61) | 9 mo | ~59 mo | 6800 | Caregiver questionnaire | Sugary beverages | Frequency in last wk | Usually consumed at age 2 y; consumed at age 5.7 y at least once/wk vs. none | Severe obesity (%) (BAZ >99th percentile, CDC 2000) (101) | At 2 y *P =* NS; At 5 y OR = 2.3, 95% CI 1.4, 3.7. | Serious |
| Huus 2009 (69) | 2.5 y | 5 y | 7356 | 7-d FFQ | SSB | Frequency/wk | Daily vs. <1 time/wk | OW/OB (%) at 5 y (Cole 2000) (102) | OR = 1.14, 95% CI 0.90, 1.45, *P =* 0.270. | Critical |
| Leermakers 2015 (74) | 12.9 mo | 59 mo | 1183 boys; 1188 girls | 1-mo FFQ | Sugar containing beverages | Servings (150mL)/wk | High (15 servings/wk) vs. low (3 servings/wk) | BAZ change (IOTF references) (103) from 2-6 y | Boys: β = 0.05, 95% CI = −0.08, 0.18, *P* = 0.42; girls β = 0.11, 95% CI = 0.00, 0.23, *P* = 0.04 at 6 y. | Moderate |
| Santorelli 2014 (84) | 6 mo | 2.5 y | 743 | Caregiver questionnaire | SSB | Consumed/not consumed <17 wk | Consumed vs. not consumed <17 wk | BMI-for-age z-score (WHO 2006) (100) | Mean diff = -0.10, 95% CI = -0·36, 0·16. | Serious |
| Pan 2014 (14) | ∼1 mo | 6 y | 1189 | 7-d recall via postal questionnaire each mo | SSB | Consumed/not consumed from 1-12 mo; Mean weekly consumption | Any: <1 time/wk; 1 to <3 times/wk; ≥ 3 times wk vs. none | OB (%) (BAZ ≥ 95^th^ percentile, CDC 2000) (101) | ≥3 times/wk (OR = 2.00, 95% CI = 1.02, 3.90); 1-<3 times/wk (OR = 1.64, 95% CI = 0.65, 3.48); <1 times/wk (OR = 1.51, 95% CI = 0.65, 3.48) vs. none. | Serious |
| Quah 2019 (12) | 18 mo | 42 mo | 767 | Self administered FFQ | SSB | mL | High vs. low intake | BAZ; OW/OB (%) (WHO 2006) (100) | High vs. low intake at 18 m and BMI z score at 6 y (β = 0.06, 95% CI = -0.20, 0.31, *P* = 0.676); % overweight/obesity (RR = 1.10, 95% CI = 0.67, 1.81, *P* = 0.204): High vs. low intake at 5 y and BMI z score at 6 y (β = 0.34, 95% CI = 0.11, 0.58, *P* = 0.004), % OW/OB (RR = 1.54, 95% CI = 1.03, 2.30, *P* = 0.033). | Serious |
| Wang 2013 (104) | 1 mo | 17 mo | 1956 | Questionnaire administered parental report on monthly intake | Sweet drinks | Frequency/wk | > 1 x wk vs. ≤ 1 x wk | OW/OB (%) (BAZ ≥ 85th percentile, WHO 2006) (100) | Unadjusted OR = 1.6, CI = 1.04, 1.93, *P* < 0.01. | Serious |
| Weijs 2011 (96) | 8.7 mo | 8 y | 120 | 2-d food record parental recorded | Beverage sugar | g/d | Continuous | BAZ; OW/OB (%) (WHO BMI z score >+1) at 8 y | BMI: β= 0.044, 95% CI 0.008, 0.080, *P* = 0.016; OW/OB: OR = 1.13, 95% CI = 1.03, 1.24, *P* = 0.009 | Critical |
| Wijga 2010 (18) | 3–12 mo | 7 y 8 mo | 1871 | 3-d weighed diet record completed by parents | Soft drink | kJ/wk | Frequency/mo (continuous) | OW/OB (%) (Cole 2000) (101) | OR = 0.91, 95% CI = 0.44, 1.88 | Serious |
|  |  |  |  |  |  |  |  |  |  |  |
| 2–<5 y | | | | | | | | | | |
| Byrne 2018 (53) | 24.1 mo | 3 y; 3.7 y; 5 y | 515 at 2 y; 405 at 5 y | Multiple pass 24-h dietary recall | Sweet beverages | Median (IQR) intake (g) of each beverage group and % of energy intake | Continuous | BAZ (WHO 2006) (100) | *P* > 0.05 | Moderate |
| De Boer 2013 (57) | 2 y | 3 y | 9600 | Parent interviewed by trained assessors at 2, 4 and 5 y | SSB | Frequency/d | ≥ 1 serving/d at 2 y vs. < 1 serving/d at 2 y | BAZ (CDC 2000) (101) at 4 y and 5 y | No estimates reported for longitudinal analysis | Serious |
| De Coen 2014 (58) | 4.95 y | 30 mo | 568 at 18 mo; 473 at 30 mo | Validated semi-quantitative 1-m FFQ | Soft drinks | mL/d | >65 mL/d vs. <65 mL/d (median intake) | OW/OB (%) (Flemish references) | OR = 1.92, 95% CI = 1.19, 3.11, P ≤ 0.01 at 18 mo; OR = 1.82, 95% CI = 1.11, 3.00, P ≤ 0.05 at 30 mo | Serious |
| Dubois 2007 (59) | 2.5 y | 2.5 y | 1499 | Self-administered FFQ at 2.5, 3.5, and 4.5 y & 24-hour recall at 4.5 y | SSB between meals | Frequency/wk | Regular consumers at age 2.5, 3.5, and 4.5 y vs. Non-consumers; Between meal consumers vs. non-between meal consumers | OB (%) (> 95th percentile, CDC 2000) (101) | Total daily consumption not significant; between meal consumption OR =2.356, 95% CI = 1.030, 5.390, P ≤ 0.05 | Moderate |
| Costa 2019 (22) | 4 y | 4 y | 315 | Two 24-h dietary recalls | Soft drinks | %EI | Continuous | BAZ change (WHO 2006) (100) | β -0.01, 95% CI -0.05 to 0.04, *P =* 0.852 | Moderate |
| Hasnain 2014 (66) | 3–5 y | 12 y | 98 | 3-d diet records | SSB | oz/d | Tertile 1 vs. 2; Tertile 1 vs. 3; Tertile 2 vs. 3 | BMI (kg/m^2^) | ANCOVA *P =* 0.0626 | Moderate |
| Millar 2014 (20) | 4.8 y | 6.1 y | 4169 | Parental reported 24-h recall, face to face interviews | SSB | Frequency/d | not at all; once/d; > once/d | BAZ (WHO 2006) (100) | β = 0.017, 95% CI = 0.007, 0.027, *P* < 0.01 | Moderate |
| Zulfiqar 2019 (17) | 4.2 y | 6 y | 2163 boys; 2044 girls | Parental report up to 8–9 y, then children self-report (computer-based) | SSB | Frequency/d | ≥ 1 vs. 0 | OW/OB (%) (IOTF references) (103): boys; girls | Boys: OR = 1.01, 95% CI = 0.8, 1.29; Girls: OR = 1.08, 95% CI = 0.87, 1.35 | Moderate |
| Macintyre 2018 (105) | 4–5 y | 3 y | 2986 | Parent interview | SSB | Frequency/wk | 1-6 times/wk; At least once/d; <once/wk | OW/OB (%) (85th and 95th percentile); Obesity (%) (UK references, Cole 1990) (106) | Overweight/obesity: OR = 1.18, 95% CI 0.63, 1.15, *P =* 0.19; Obese: OR= 1.65, 95% CI = 1.12, 2.44, *P =* 0.01 | Moderate |
| Marshall 2019 (9) | 2–4.7 y | 12.3–15 y | 454 | 7-d beverage frequency questionnaire | Beverages | Servings (236.5mL)/d | Continuous | BAZ (CDC 2000) (101) | β = 0.05, 95% CI = 0.022, 0.079, *P =* 0.001 | Moderate |
| Newby 2004 (81) | 2.9 y | 8.4 mo | 1345 | 1-mo FFQ | Soda | oz/d | Continuous | BMI/y | Fruit drinks: β = -0.01, SE = 0.00, *P =* 0.20; Soda: β = -0.01, SE = 0.02, *P =* 0.50 | Moderate |
| Welsh 2005 (99) | 33.8 mo | ~1 y | 10904 | FFQ | Sweet drinks | Drinks/d | 1≤2; 2≤3; ≥ 3 vs. 0≤1 times/d | Odds of OW/OB based on baseline BMI category status (CDC, 2000) (101) ≥ 95th percentile in those with BMI <85th percentile at baseline; BMI ≥ 95th percentile in those with BMI 85th-<95th percentile at baseline; BMI ≥ 95th percentile in those with ≥95th percentile at baseline | OR = 1.3, 95% CI = 0.8, 2.1, ≥ 3/d vs. 0-<1/d among those normal weight at baseline | Moderate |
|  |  |  |  |  |  |  |  |  |  |  |
| 5– 10 y | | | | | | | | | | |
| Alviso-Orellana 2018 (48) | 8 y | 4 y | 1414 | 30-d recall | SSB | Frequency per 2 wk/wk/d | up to every 2 wk; 2-6 times/wk; daily or never | BMI change (kg/m^2^); OW/OB (%) (107) | β = 0.74, 95% CI = 0.15, 1.33, daily vs. no intake; OW/OB aRR = 2·12, 95% CI 1·05, 4·28 | Moderate |
| Bayer 2014 (50) | 6.0 y | 4 y | 1252 | Self administered parental questionnaire | High-caloric drinks | Servings (200mL)/d | Continuous | Normal weight/overweight assessed by BMI z score | No estimates reported | Serious |
| Blum 2005 (51) | 9.3 y | 2 y | 164 | 24-h dietary recall | SSB | oz/d | Continuous | Amount of SSB consumed according to BMI category (using CDC 2000) (101) | ANOVA *P >* 0.05, unadjusted | Critical |
| Carlson 2012 (8) | 6.7 y | 24 mo | 254 | Parent survey average consumption/d | SSB | Servings (354mL)/d | Continuous | BAZ (CDC 2000) (101) | Unstandardized β = 0.11, CI = -0.03, 0.25, *P =* 0.124 | Serious |
| Fiorito 2009 (60) | 5 y | 10 mo | 166 | Three 24-h recalls | Sweetened beverage | Servings (236.5mL)/d | ≥1 and <2 servings/d vs. <1 servings/d; ≥2 servings/d vs. <1 servings/d | BMI (kg/m^2^) | ANOVA exposure group: *P =* NS; age: NS; group* age: NS) | Serious |
| Zheng 2015 (64) | 8 y | 3.5 y | 158 | Three 24-h recalls using multiple pass approach at age 9 y by telephone | SSB | g/d | per 100g/d | BAZ change (CDC 2000) (101) | β = 0.10, SE = 0.03, *P =* 0.003 | Serious |
| Hur 2015 (68) | 9.9 y | 4 y | 605 | 3-d food record | Beverage sugar | g/d | Continuous | BAZ (Korean growth standards) | β = -0.02, SE = 0.03, *P >* 0.05 | Serious |
| Lim 2009 (13) | 6.7 y | 8.7 y | 254 | Quantitative FFQ | All SSB | oz/d | Continuous | Overweight/obesity (%) (BMI ≥ 85th percentile (CDC 2000) (101) | OR = 1.04, 95%CI = 1.01,1.07, *P* < 0.05 | Moderate |
| Jackson 2017 (71) | 5.6 y | 9 y | 4938 | 1-wk FFQ | SSB | Servings (no estimated volume)/d | Times /d or times/wk (7 categories) | BAZ (CDC 2000) (101) | *P >* 0.05 (parameter estimate from a cross-lagged autoregressive model) | Moderate |
| Jensen 2013a (34) | 6.7 y | 13.3 y | 324 | FFQ average of 5 and 7 y | SSB | kJ/d | Continuous | BMI change (kg/m^2^) | Sweet drinks Intake at 6 y and BMI change 6–9 y (β = -0.014, 95% CI = -0.063, 0.035, *P =* 0.55), 6–13 y (β = -0.049, 95% CI = -0.1299, 0.024, *P =* 0.18) or 9–13 y (β = -0.036, 95% CI = -0.017, 0.088, *P =* 0.17). SSB (soft drinks and squash only) intake at 6 y and BMI change 6–9 y (β = -0.005, 95% CI = -0.059, 0.049, *P =* 0.84); 6–13 y (β = -0.059, 95% CI = -0.145, 0.027, *P =* 0.17) or 9–13 y (β = 0.008, 95% CI = -0.098, 0.113, *P =* 0.88) | Moderate |
| Laurson 2008 (73) | Boys 10.8 y; Girls 10.7 y | 18 mo | 146 boys; 122 girls | Questionnaire | SSB; Change in SSB consumption | Servings (no estimated volume)/wk | Continuous | BMI change (kg/m^2^) | Baseline intake: boys β = 0.114, SE = 0.021, *P =* 0.184, girls β = 0.022, SE = 0.021, *P =* 0.821; Change in intake baseline to follow up: boys β = -0.037, SE = 0.019, *P =* 0.707, girls β = 0.086, SE = 0.027, *P =* 0.450 | Moderate |
| Muckelbauer 2016 (11) | 8.3 y | ~10 mo | 1987 | Semi-quantitative 24-h recall | Sugar-containing beverages | Glasses (200mL)/d | Continuous | BMI change (kg/m^2^); OW/OB (%)(Cole 2000) (102) | % OB: OR 1·22; 95% CI 1·04, 1·44, *P =* 0.014; OW/OB *P =* 0.83; BMI change: β = 0.02, 95% CI 0.00, 0.03 | Some concerns |
| Olsen 2012 (83) | Boys 9.7 y; Girls 9.4 y | 6 y | 359 | 24-h recall interview, FFQ and a qualitative food record | Liquid sucrose | per 10g intake | Continuous | Change in BAZ (Cole & Green 1992) (108) | β = 0.024, SE = 0.017, *P =* 0.17 | Critical |
| Striegel-Moore 2006 (88) | 9-10 y | 10 y | 2371 | 3-d food record | Regular soda | g/d | Continuous | BMI (kg/m^2^) | SSB: β = 0.011, SE = 0.005*, P* < 0.05; Fruit juice (not 100%): β = 0.005, SE = 0.007, *P* > 0.05; Fruit drinks (β = 0.009, SE = 0.007, *P* > 0.05 | Serious |
| Traub 2018 (92) | 7.08 y | 1 y | 1250 | Questionnaire completed by parents | Soft drinks | Frequency/d; Frequency/wk | > 1 time/wk vs. <1 time/wk | Overweight only (%) BMI >90th percentile; obesity (%) BMI >97th percentile (German references) | Overweight only OR = 1.29, 95% CI = 0.84, 1.96, *P =* 0.246; obese only OR = 1.57, 95% CI = 0.82, 3.03, *P =* 0.177 | Moderate |
| Zheng 2014 (97) | 9.6 y | 12 y | 171 | 24-h recall; face-to-face interview and qualitative food record | SSB | Servings (354mL)/d | > 1 serving/d vs. ≤ 1 serving/d | BMI change (kg/m^2^) | at 9 y > 1 serve β= 1.42, SE 0.68, *P =* 0.29; <= 1 serve β = 0.53 SE 0.55, *P =* 0.34; at 15 y > 1 serve β = 0.85, SE 0.54 *P =* 0.12, <= 1 serve: β = 0.58, SE 0.56 *P =* 0.30 | Moderate |
|  |  |  |  |  |  |  |  |  |  |  |
| **ASB** | | | | | | | | | | |
| <10 y | | | | | | | | | | |
| Blum 2005 (51) | 9.3 y | 2 y | 164 | 24-h dietary recall | Diet soda | oz/d | Continuous | BAZ (CDC 2000) (101) | ANOVA *P <* 0.05 | Critical |
| Zheng 2015 (64) | 8 y | 3.5 y | 158 | Three 24-h recalls using multiple pass approach at age 9 y by telephone | Diet drinks | g/d | Intake per 100 g/d | BAZ change (CDC 2000) (101) | β = -0.20, SE = 0.07, *P =* 0.01 | Serious |
| Hasnain 2014 (66) | 3–5 y | 12 y | 98 | 3-d diet records | ASB | oz/d | Tertile 1 vs. 2; Tertile 1 vs. 3; Tertile 2 vs. 3 | BMI (kg/m^2^) | ANCOVA *P =* 0.444‡ | Moderate |
| Macintyre 2018 (105) | 4–5 y | 3 y | 2986 | Parent interview | ASB | Frequency/wk | At least once/d; 1 - 6 time/wk; <once/wk | OW/OB (%) (85th and 95th percentile); Obese (%) (Cole 1990) (109) | OW/OB: OR = 0.85, 95% CI 0.63, 1.15 *P =* 0.85; obesity: OR = 1.57, 95% CI = 1.05, 2.36, *P =* 0.03 | Moderate |
| Newby 2004 (81) | 2.9 y | 8.4 mo | 1345 | 1-mo FFQ | Diet soda | oz/d | Continuous | BMI/y | β = 0.01, SE = 0.02, *P =* 0.83 | Moderate |
| Striegel-Moore 2006 (88) | 9–10 y | 10 y | 2371 | 3-d food record | Diet soda | g/d | Continuous | BMI (kg/m^2^) | β = 0.01, SE = 0.013, *P >* 0.05 | Serious |
|  |  |  |  |  |  |  |  |  |  |  |
| **100% fruit juice** | | | | | | | | | | |
| 0–<2 y | | | | | | | | | | |
| Budree 2017 (52) | Birth | 12 mo | 1076 | FFQ items consumed on a daily, weekly, and monthly basis | Fruit juice | Daily consumption | Daily vs. less than daily | OW/OB (%) (WHO 2006) (100) | Unadjusted OR = 1.0, 95% CI = 0.5, 2.0, *P =* 0.916 | Serious |
| Guerrero 2016 (65) | 9 mo | 63 mo | 15418 | Parent interview at 48, 60, and 72-mo | Juice | Intake vs. no intake in last 7 d | Any vs. none |  |  | Moderate |
| Sonneville 2015 (15) | 1 y | median of 2.1 y and 6.7 y | 1038 | FFQ | 100% fruit juice | oz/d | Large (≥16 oz/d) vs. none; Medium (8-15 oz/d) vs. none; Small (1-7oz/d) vs. none | BMI z score (US growth reference) | β=0.30, 95% CI = −0.01, 0.61 at 2.1 y; β=0.27, 95% CI = −0.05, 0.59 at 6.7 y | Moderate |
| 2–<5 y | | | | | | | | | | |
| Hasnain 2014 (66) | 3–5 y | 12 y | 98 | 3-day diet records | Fruit and vegetable juices | oz/d | Tertile 1 vs. 2; Tertile 1 vs. 3; Tertile 2 vs. 3 | BMI (kg/m^2^) | ^3^ANCOVA *P =* 0.062 | Moderate |
| Alexy 1999 (75) | Boys 3 y; Girls 3 y | Boys 5 y; Girls 5.1 y | 205 | 3-d weighed diet record by parents | Fruit juice | g/d | Continuous | BMI (kg/m^2^) | No estimates reported | Critical |
| Marshall 2019 (9) | 2–4.7 y | 12.3-15 y | 454 | 7-d beverage frequency questionnaire | 100% juice | Servings (236.5mL)/d | Continuous | BAZ (CDC 2000) (101) | β = -0.001, 95% CI = -0.059, 0.057, *P =* 0.97 | Moderate |
| Newby 2004 (81) | 2.9 y | 8.4 mo | 1345 | 1-mo FFQ | Fruit juice only | oz/d | Continuous | BMI/y | β = 0.01 SE = 0.00, *P =* 0.20 | Moderate |
| Shefferly 2016 (85) | 2 y | 2–3 y | 6250 | 7 d recall frequency | Fruit juice at 2-4 y; Fruit juice at 4-5 y | Servings (236.5mL)/d | ≥ 1 serving/d vs. <1 serving/d | Change in BAZ; overweight (%); obese (%) (CDC 2000) (101) | Mean BMI z score change 0.282 (SE 0.028) vs. 0.030 (SE 0.037), *P =* 0.0003 at 2–4 y, 0.034 (SE 0.031) 0.020 (SE 0.021) *P =* 0.6778 at 4–5 y; % OW/OB OR = 1.30, 95% CI = 1.06-1.59, *P =* 0.0129 at 2–4 y; OR = 0.80, 95% CI = 0.43-1.49, P= 0.473 at 4–5 y | Moderate |
| Skinner 2001 (110) | 27 mo | 4 y | 72 | 24-h recall and 2-d weighed food records | 100% fruit juice | oz/d | Continuous | BMI (kg/m^2^) | β = -0.057, *P =* 0.099 (SE not stated) | Serious |
| Wan 2020 (94) | 3–6 y | 10 y | 100 | Multiple sets of 3-day diet records | 100% fruit juice | Cup equivalent/d | <0.5 cups; 0.5 ≤1.0 cups; ≥1.0 cups | BMI (kg/m^2^) | No estimates reported | Serious |
| Welsh 2005 (99) | 33.8 mo | ~1 y | 10904 | FFQ | Fruit juices only | Drinks/d | 1-<2/d vs. 0-<1/d; 2-<3/d vs. 0-<1/d; ≥ 3/d vs. 0-<1/d | Odds of OW/OB based on baseline BMI category status (CDC, 2000) (101) ≥ 95th percentile in those with BMI <85th percentile at baseline; BMI ≥ 95th percentile in those with BMI 85th-<95th percentile at baseline; BMI ≥ 95th percentile in those with ≥ 95th percentile at baseline | Odds of overweight among those normal weight at baseline for intake 1-<2 /d (OR = 1.1, 95% CI = 0.8, 1.5); 2-<3/d (OR = 1.0, 95% CI = 0.7, 1.4) or ≥ 3/d (OR = 1.2, 95% CI = 0.8, 1.7) compared to 0-<1/d. Odds of overweight for at risk for overweight at baseline 1-<2/d (OR = 1.1, 95% CI = 0.8, 1.6), 2-<3/d (OR = 1.0, 95% CI = 0.7, 1.4) or ≥ 3/d (OR = 0.8, 95% CI = 0.5, 1.1) | Moderate |
| 5– 10 y | | | | | | | | | | |
| Blum 2005 (51) | 9.3 y | 2 y | 164 | 24-h dietary recall | 100% juice | oz/d | Continuous | BMI category based on BAZ (CDC 2000) (101) | ANOVA *P >* 0.05 | Critical |
| Carlson 2012 (8) | 6.7 y | 24 mo | 254 | Parent survey average consumption/day | 100% fruit or vegetable juice | Servings (236.5mL)/d | Continuous | BAZ (CDC 2000) (101) | Unstandardized β = -0.04, CI = -0.21, 0.13, *P =* 0.631 | Serious |
| Zheng 2015 (64) | 8 y | 3.5 y | 158 | Three 24-h recalls using multiple pass approach at age 9 y | 100% fruit juices | g/d | Intake per 100g/d | BAZ change (CDC 2000) (101) | β = 0.07, SE = 0.05, *P =* 0.12 | Serious |
| **Unhealthy foods^4^** | | | | | | | | | | |
| 0–<2 y | | | | | | | | | | |
| Garden 2011 (62) | 18 mo | 6.5 y | 362 | 3-d weighed food record | Extra foods (cookies, crackers, juice, cordial, fruit drinks and soft drinks, fats and oils, snack foods, sugar, confectionary, savory sauces, condiments, fried potatoes, ice-cream, and some miscellaneous foods); Dairy products (milk and milk products, including yoghurt, cheese, ice cream and custard) | g/d | Quintiles of intake as g/d | BMI (kg/m^2^) | Extra foods' β = -0.10, 95% CI = -0.30, 0.11, *P =* 0.36 for trend; dairy products β = -0.21, 95% CI = -0.41, 0.01, *P =* 0.04 for trend | Moderate |
| Huus 2009 (69) | 2.5 y | 5 y | 7356 | 7-d FFQ | Fried potato/French fries; Sausage; Cream/creme fraiche; chips; Cheese; Pastries; Chocolate; Candy (non-chocolate); Ice-cream | Frequency/wk | Daily vs. <1 time/wk (1-2 times/wk vs. <1 time/wk for fried potato) | OW/OB (%) at 5 y (Cole 2000) (102) | Fried potato/French fries consumption at 2.5 y OR = 0.75, 95% CI = 0.62, 0.92, *P =* 0.006) daily consumption at 5 y *P >* 0.05. Daily consumption of sausage, cream/crème fraiche, chips, cheese, chocolate and ice-cream consumption at either 2.5 or 5 y had no significant effect on the risk of OW/OB at 5 y (*P >* 0.05). Daily consumption of pastries and consumption of candy at 2.5 y (*P >* 0.05); 5 y (pastries OR = 0.46, 95% CI = 0.23, 0.90, *P =* 0.023; candy OR = 1.6, 95% CI = 1.22, 2.12, *P =* 0.001 | Critical |
| Santorelli 2014 (84) | 6 mo | 2.5 y | 743 | Caregiver questionnaire | Sweetened first foods | Consumed/not consumed | Consumed vs. not consumed | BMI-for-age z-score (WHO 2006) (100) | Mean difference = 0.03 95% CI = -0.12, 0.19 | Serious |
| Thurber 2017 (91) | 0.5–2 y and 3–5 y; 0.5–2 y; 3–5 y |  | 907 | Caregiver report | High fat foods | Frequency/d | ≥ 2 times/d in last 24 h vs. <2 times/d in last 24-h | BMI (kg/m^2^) |  | Serious |
| Wijga 2010 (18) | 3–12 mo | 7 y 8 mo | 1871 | 3-d weighed diet record completed by parents | Fast food; Sweet and savory snacks | kJ/wk | Times/mo (continuous) | OW/OB (%) (Cole 2000) (102) | Fast foods: OR = 1.14, 95% CI = 0.77, 1.67; Snack consumption OR = 0.71, 95% CI = 0.52, 0.98 | Serious |
|  |  |  |  |  |  |  |  |  |  |  |
| 2–<5 y | | | | | | | | | | |
| De Coen 2014 (58) | 4.95 y | 30 mo | 568 at 18 mo; 473 at 30 mo |  | Sweet and savory snack consumption | g/d | >54 g/d vs. <54 g/d (median intake) | OW/OB (%) (Flemish references) | OR = 0.76, 95% CI = 0.41, 1.40, *P >* 0.05 | Serious |
| Emond 2020 (29) | 3–5 y | 1 y | 541 | Parent reported usual frequency | Fast food | Frequency of consumption/wk | ≥3.1 vs. >1.1 to 2.0 times/wk | Change in BMI status (normal to overweight or overweight to obese) | RR: 1.38, 95% CI 1.13, 1.67, *P <* 0.01 | Moderate |
| Costa 2019 (22) | 4 y | 4 y | 315 | 12-mo FFQ | Ultra-processed foods | %EI | Continuous | BMI change (kg/m^2^) | β = 0.05, 95%CI = -0.04, 0.15, *P =* 0.282 | Moderate |
| Millar 2014 (20) | 4.8 y | 6.1 y | 4169 | Parental reported 24-h recall, face to face interviews | High fat foods (1) meat pie, hamburger, hotdog, sausage or sausage roll; (2) hot chips or French fries; (3) potato chips or savory snacks; 4) biscuits, doughnuts, cake, pie or chocolate) | Frequency/d | 0 “not at all”; 1 “once/d”; 2 “more than once"/d | BMI z score | β = 0.021, 95% CI 0.014, 0.029 *P <* 0.001 | Moderate |
| Zulfiqar 2019 (17) | 4.2 y | 6 y | 2163 boys; 2044 girls | Parental report up to 8–9 y, then children self-reported (computer-based) | High fat foods | Frequency/d | >=1 vs. 0 | OW/OB v non-OW/OB in boys and girls | Boys: OR = 0.85, 95% CI = 0.6, 1.19; Girls: OR = 0.97, 95% CI = 0.7, 1.35 | Moderate |
| Buyken 2008 (23) | 2 y | 5 y; 6 y | 380 | 3-d weighed dietary record | Added sugar (white sugar, brown sugar, raw sugar, corn syrup, corn-syrup solids, high-fructose corn syrup, malt syrup, maple syrup, fruit syrup, pancake syrup, fructose sweetener, liquid fructose, honey, molasses, anhydrous dextrose, and crystal dextrose) | %EI | Continuous | Change in BAZ (German references) | β = -0.001, SE = 0.010, *P =* 0.9 | Serious |
| Herbst 2011 (24) | 1 y | 6 y | 216 | 3-d weighed dietary record | Total added sugars (added sugar from beverages (regular and diet soft drinks, fruit juices) + sweets (candy, chocolate jam and ice cream) + other sources (BF cereals, pastries, milk and milk products)) : Added sugar from beverages and sweets : Added sugar from other sources (BF cereals, pastries, milk and milk products) | %EI | Continuous | Change in BAZ (German references) | Intake at 1 y: β = -0.116, SE = 0.057, *P =* 0.04 at 7 y. Change in intake 1-2 y: β = 0.074, SE = 0.043, *P =* 0.09) at 7 y | Serious |
| Russo 2018 (21) | Boys 4.2 and 7.4 y; Girls 4.2 and 7.4 y | 2 y | 6929 | FFQ via Children’s Eating Habits Questionnaire | Added sugars to milk and fruit | Daily/weekly/rarely | Daily (once or more times/d) vs. Rarely (never/less than once a wk); Weekly (<1/d) vs. Rarely (never/less than once a wk) | BMI (kg/m^2^) | 2 < 6 y: boys *P =* 0.005, girls *P =* 0.03; 6 < 10 y: boys *P =* 0.001, Girls *P >* 0.05 | Moderate |
| Durao 2015 (93) | 2 y | 2 y | 589 | FFQ and 3-d food diaries | Energy-dense foods (salty snacks, soft drinks, cakes, and sweets) | Frequency/d | Continuous | BMI z score (Cole 2000) (102) | β = -0.051, 95% CI = -0.135, 0.034 | Moderate |
| Vedovato 2020 (25) | 4 y | 6 y | 1175 | 2-d or 3-d food diaries including quantities | Ultra-processed foods | %EI | Continuous per 100 kcal intake | BMI z score (WHO 2006) (100) | β = 0·028; 95% CI = 0.006, 0.051 intake at 4 y; β=0·014; 95%CI = -0.007, 0.036 intake at 7 y | Moderate |
| 5– 10 y | | | | | | | | | | |
| Alviso-Orellana 2018 (48) | 8 y | 4 y | 1414 | 30-d recall | Snacks-salty and fatty foods (crisps, fried snacks) | Frequency/2 wk/wk/d | Up to every 2 wk; 2-6 times/wk; daily or never | BMI change (kg/m^2^), OW/OB (%) (107) | Everyday vs never β = 0.71, 95% CI = 0.14, 1.28; relative risk=1.43 0.78, 2.69 | Moderate |
| Bayer 2014 (50) | 6.0 y | 4 y | 1252 | Self administered parental questionnaire | Energy dense sweets | Consumed/not-consumed | Consumed vs. not-consumed |  | No estimates reported | Serious |
| Bel-Serrat 2019 (19) | 7.9 y | 4 y | 2755 | 7-d recall | Fast food (Pizza, fries, hamburger etc.); savory snacks (crisps, popcorn, peanuts etc.) | ‘never/< once a wk’, ‘some days (1–3 d)’, ‘most days (4–6 d)’, ‘every day’. | Everyday vs. never | OW/OB (%)(Cole 2000) (102) | Savory snack intake some days/wk (OR = 0.48, 95% CI = 0.23, 0.99, *P <* 0.05), never (OR = 0.27, 95% CI = 0.10, 0.72, *P <* 0.01) vs. everyday. Fast food intake some days/wk (OR = 0.88, 95% CI = 0.27, 2.83, *P >* 0.05), never (OR = 0.91, 95% CI = 0.19, 4.31, *P >* 0.05) vs. everyday | Serious |
| Carlson 2012 (8) | 6.7 y | 24 mo | 254 | Parent survey average consumption/d | High fat foods (fried chicken, pizza, whole or 2% milk, French fries, tater tots, onion rings) | Servings/d | Continuous | BAZ (CDC 2000) (101) | Unstandardized β = -0.02, CI = -0.06, 0.03, *P =* 0.409 | Serious |
| Hur 2015 (68) | 9.9 y | 4 y | 605 | 3-d food record | Other sugar (sweets, sweetened grains, sweetened dairy products, sugars, syrup and natural sugar from vegetables and grains). | g/d | Continuous | BAZ (Korean growth standards) | β = 0.16, SE = 0.10, *P >* 0.05 | Serious |
| Jackson 2017 (71) | 5.6 y | 9 y | 4938 | 1-wk FFQ | Fast food | Servings/d | Frequency/d or /wk (7 categories) | BAZ (CDC 2000) (101) | *P >* 0.05 (parameter estimate from a cross-lagged autoregressive model) | Moderate |
| Lissau 1993 (76) | 9–10 y | 11 y | 512 | Postal survey to parents | Candy | Frequency/d | Frequent vs. infrequent consumption | Overweight (%) (BMI >90th percentile, internal z scores) | OR = 0.5, CI = 0.1, 1.4, *P >* 0.05 | Critical |
| Olsen 2012 (83) | Boys 9.7 y; Girls 9.4 y (combined in analysis) | 6 y | 359 | 24-h recall interview, FFQ and a qualitative food record | Added sugar; Solid sucrose (added sugar/sucrose + liquid sucrose) | per 10g intake | Continuous | Change in BAZ (Cole & Green 1992) (108) | Added sugar intake: β = 0.012, SE = 0.011, *P =* 0.26; Solid sugar intake: β = 0.000, SE = 0.0016, *P =* 0.99 | Critical |

^1^ASB, artificially-sweetened beverages; BAZ, BMI-for-age z score; DAT, dietary assessment tool; FFQ, food-frequency questionnaire; NS, not stated; OW/OB, overweight including obesity; OB, obesity only; %EI, percentage of energy intake; RoB, risk of bias; SSB, sugar-sweetened beverages.

^2^Minimum analytical sample size.

^3^Adjusted, unless otherwise stated.

^4^Includes energy-dense, nutrient poor and ultra-processed foods.

^5^1 fluid oz (US) = 29.6 mL

^6^1 kJ = ~0.24 kcal

^7^1 cup equivalent = ~250 mL

**Supplemental Table 10** Synthesis of results from included studies of the effects of unhealthy food and beverage consumption among children ≤10.9 y on body fat outcomes^1^

| **Reference** | **Baseline age (mean or range)** | **Follow-up duration** | **N^2^** | **DAT** | **Exposure** | **Intake unit** | **Comparator** | **Outcomes** | **Estimate** | **Overall RoB** |
| --- | --- | --- | --- | --- | --- | --- | --- | --- | --- | --- |
| **SSB** | | | | | | | | | | |
| 0–<2 y | | | | | | | | | | |
| Leermakers 2015 (74) | 12.9 mo | 59 mo | 1183 boys; 1188 girls | 1-m FFQ | Sugar containing beverages | Servings (150mL)/wk | High (15 servings/wk) vs. low (3 servings/wk) | %BF (DXA) | Boys β= 0.05, 95% CI = -0.11, 0.20, *P =* 0.53; girls β = 0.09, 95% CI = -0.06, 0.23, *P =* 0.25 high vs. low intake | Moderate |
| 2–<5 y | | | | | | | | | | |
| Hasnain 2014 (66) | 3–5 y | 12 y | 98 | 3-d diet records | SSB | oz/d | Tertile 1 vs. 2; Tertile 1 vs. 3; Tertile 2 vs. 3 | %BF (DXA) | ANCOVA *P =* 0.929 | Moderate |
| 5– 10 y | | | | | | | | | | |
| Carlson 2012 (8) | 6.7 y | 24 mo | 254 | Parent survey average consumption/d | SSB | Servings (354 mL)/d | Continuous | %BF (BIA) | Unstandardized β = 1.40, CI = 0.09, 2.72, *P =* 0.036 | Serious |
| Johnson 2007 (56) | 5.2 y; 7.2 y | ~4 y | 362 | 3-d unweighed diet diaries | SSB | g/d; serving/d (1 serving = 180g) | Continuous | Change in fat mass (kg) (DXA) per serving | β = -0.15, 95% CI = -0.54, 0.24, *P =* 0.45 | Moderate |
| Fiorito 2009 (60) | 5 y | 10 mo | 166 | Three 24-h recalls | Sweetened beverage | Servings (236.5 mL)/d | ≥1 and <2 servings/d vs. <1 servings/d; ≥2 servings/d vs. <1 servings/d | %BF (SSF) | ANOVA group *P <* 0.01, age *P <* 0.01, group x age *P <* 0.01 | Serious |
| Zheng 2015 (64) | 8 y | 3.5 y | 158 | Three 24-h recalls using multiple pass approach at age 9 y by telephone | SSB | g/d | per 100g/d | %BF (BIA) | β = 1.04, SE = 0.32, *P =* 0.001 | Serious |
| Hur 2015 (68) | 9.9 y | 4 y | 605 | 3-d food record | Beverage sugar | g/d | Continuous | %BF (BIA) | β = 0.02, SE = 0.21, *P >* 0.05 | Serious |
|  |  |  |  |  |  |  |  |  |  |  |
| **ASB** | | | | | | | | | | |
| 2–<5 y | | | | | | | | | | |
| Johnson 2007 (56) | 5.2 y; 7.2 y | ~4 y | 362 | 3-d unweighed diet diaries | Low energy drinks | g/d; serving/d (1 serving = 180g) | Continuous | Change in fat mass (kg) (DXA) per serving | β = 0.26, 95%CI = -0.004, 0.52, *P =* 0.05 | Moderate |
| Zheng 2015 (64) | 8 y | 3.5 y | 158 | Three 24-h recalls using multiple pass approach at age 9 y by telephone | Diet drinks | g/d | Intake per 100 g/d | %BF (BIA) | β = -1.41, SE = 0.70, *P =* 0.046 | Serious |
| Hasnain 2014 (66) | 3–5 y | 12 y | 98 | 3-d diet records | ASB | oz/d | Tertile 1 vs. 2; Tertile 1 vs. 3; Tertile 2 vs. 3 | %BF (DXA) | ANCOVA *P =* 0.584 | Moderate |
|  |  |  |  |  |  |  |  |  |  |  |
| **100% fruit juice** | | | | | | | | | | |
| 2–<5 y | | | | | | | | | | |
| Hasnain 2014 (66) | 3–5 y | 12 y | 98 | 3-day diet records | Fruit and vegetable juices | oz/d | Tertile 1 vs. 2; Tertile 1 vs. 3; Tertile 2 vs. 3 | %BF (DXA) | ANCOVA *P =* 0.119 | Moderate |
| 5–<10 y | | | | | | | | | | |
| Carlson 2012 (8) | 6.7 y | 24 mo | 254 | Parent survey average consumption/day | 100% fruit or vegetable juices | Servings (236.5 mL)/d | Continuous | %BF (BIA) | Unstandardized β= -1.06, CI = -2.70, 0.57, *P =* 0.202 | Serious |
| Johnson 2007 (56) | 5.2 y; 7.2 y | ~4 y | 362 | 3-d unweighed diet diaries | 100% fruit juices | g/d + serving/d (1 serving = 180g) | Continuous | Change in fat mass (kg) (DXA)/serving | β = -0.11, 95% CI = -0.61, -0.38, *P =* 0.66 | Moderate |
| Zheng 2015 (64) | 8 y | 3.5 y | 158 | Three 24-h recalls using multiple pass approach at age 9 y | 100% fruit juices | g/d | Intake per 100g/d | %BF (BIA) | β = -0.05, SE = 0.44, *P =* 0.91 | Serious |
|  |  |  |  |  |  |  |  |  |  |  |
| **Unhealthy foods^3^** | | | | | | | | | | |
| 2–<5 y | | | | | | | | | | |
| Buyken 2008 (23) | 2 y | 5 y; 6 y | 380 | 3-d weighed dietary record | Added sugar (white sugar, brown sugar, raw sugar, corn syrup, corn-syrup solids, high-fructose corn syrup, malt syrup, maple syrup, fruit syrup, pancake syrup, fructose sweetener, liquid fructose, honey, molasses, anhydrous dextrose, and crystal dextrose.) | %EI | Continuous | Change in %BF (SSF) | β = 0.048, SE = 0.046, *P =* 0.3 | Serious |
| Herbst 2011 (24) | 1 y | 6 y | 216 | 3-d weighed dietary record | Total added sugars (added sugar from beverages (regular and diet soft drinks, fruit juices) + sweets (candy, chocolate jam and ice cream) + other sources (BF cereals, pastries, milk, and milk products)): Added sugar from beverages and sweets: Added sugar from other sources (BF cereals, pastries, milk and milk products) | %EI | Continuous | Change in %BF (SSF) | Intake at 1 y: β = -0.014, SE = 0.015, *P =* 0.4 at 7 y; Change in intake 1-2 y: β = 0.002, SE = 0.012, *P =* 0.8 | Serious |
| Russo 2018 (21) | Boys 4.2 and 7.4 y; Girls 4.2 and 7.4 y | 2 y | 6929 | FFQ via Children’s Eating Habits Questionnaire | Added sugars to milk and fruit | Daily/weekly/rarely | Daily (once or more times/d) vs. Rarely (never/less than once a wk); Weekly (<1/d) vs. Rarely (never/less than once a wk) | %BF (SSF) | 2< 6 y boys *P =* 0.009; girls *P >* 0.05: 6 < 10 y, boys *P =* 0.001, girls *P >* 0.05 | Moderate |
| 5– 10 y | | | | | | | | | | |
| Carlson 2012 (8) | 6.7 y | 24 mo | 254 | Parent survey average consumption/d | High fat foods (fried chicken, pizza, whole or 2% milk, French fries, tater tots, onion rings) | Servings/d | Continuous | %BF (BIA) | unstandardized β = -0.38, CI = -0.81, 0.05, *P =* 0.081 | Serious |
| Costa 2020 (55) | 6 y | 5 y | 3514 | 12-mo retrospective FFQ | Ultra-processed foods | Annual consumption in g at 6 y and 11 y | Continuous | FMI^4,5^ | β = 0.05, 95% CI = 0.04, 0.06, *P <* 0.001 | Moderate |
| Hur 2015 (68) | 9.9 y | 4 y | 605 | 3-d food record | Other sugar, sweetened grains, sweetened dairy products, sugars, syrup and natural sugar from vegetables and grains. | g/d | Continuous | %BF (BIA) | β = 0.83, SE = 0.72, *P >* 0.05 | Serious |

^1^ASB, artificially-sweetened beverages; BIA, bioelectrical impedance; DAT, dietary assessment tool; DXA, dual x-ray absorptiometry; FMI, fat mass index; FFQ, food-frequency questionnaire; NS, not stated; OW/OB, overweight including obesity; OB, obesity only; %BF, percentage body fat; %EI, percentage of energy intake; RoB, risk of bias; SSB, sugar-sweetened beverages; SSF, sum of skinfolds.

^2^Minimum analytical sample size.

^3^Includes energy-dense, nutrient poor and ultra-processed foods.

^4^Fat mass index was calculated by dividing fat mass (kg) by height (m^2^).

^5^Body fat was assessed via air displacement plethysmography

^6^1 fluid oz =(US) = 29.6 mL

**Supplemental Table 11** Synthesis of results from included studies of the effects of unhealthy food and beverage consumption among children ≤10.9 y on other growth and body composition outcomes^1^

| **Reference** | **Baseline age (mean or range)** | **Follow-up duration** | **N^2^** | **DAT** | **Exposure** | **Intake unit** | **Comparator** | **Outcomes** | **Estimate** | **Overall RoB** |
| --- | --- | --- | --- | --- | --- | --- | --- | --- | --- | --- |
| **SSB** | | | | | | | | | | |
| 0–<2 y | | | | | | | | | | |
| Cantoral 2016 (54) | Birth | 8–14 y | 227 | 3-m FFQ | SSB | Cumulative consumption in pre-school years | Third vs. first tertile SSB cumulative consumption | Abdominal obesity (%) (WC >90th centile) | High vs. low: OR = 2.70, 95% CI: 1.03, 7.03, mid vs. low = P > 0.05 | Serious |
| Guerrero 2016 (65) | 9 mo | 63 mo | 15418 | Parent interview at 48, 60, and 72-mo | Soda | Intake vs. no intake in last 7 d | Any vs. none in last 7 d | BMI trajectory | β = 0.138, SE = 0.037, P < 0.01 | Moderate |
| Hwang 2020 (70) | 5 mo | 69 mo | 12777 | Diet questionnaire | SSB at 21 mo; SSB at 33 mo; SSB at 45 mo | *cc*/d | ≥ 200 cc/d vs. <200 cc/d | Adiposity rebound | Difference at 21 mo P = 0.02; at 33 m P = 0.71; at 45 mo P = 0.71 | Serious |
| Kramer 2004 (72) | 1 mo | 11 mo | 16491 | NS | Other liquids | Yes/No | Consumed vs. not-consumed | Weight-for-age | 9-12 mo (Point estimate = 0.026, 95% CI = -0.016, 0.069, P > 0.05) | Moderate |
| Leermakers 2015 (74) | 12.9 mo | 59 mo | 1183 boys; 1188 girls | 1-m FFQ | Sugar containing beverages | Servings (150 mL)/wk | High (15 servings/wk) vs. low (3 servings/wk) | Android/gynoid fat ratio | boys, β = 0.02, 95% CI = -0.14, 0.18, P = 0.77; girls, β= 0.14, 95% CI = -0.02, 0.29, P = 0.09 | Moderate |
| Quah 2019 (12) | 18 mo | 42 mo | 767 | Self administered FFQ | SSB | mL | High vs. low intake | SSF (mm) | Intake at 18 mo and outcome at 5 y OR = -0.46, 95% CI = -3.27, 2.34, P = 0.850; at 6 y OR = 2.2, 95% CI = -0.38, 4.78, P = 0.0096 | Serious |
| Thurber 2017 (91) | 0.5–2 y and 3–5 y; 0.5–2 y; 3–5 y |  | 907 | Caregiver report | SSB | Frequency/day | ≥ 2 times/d vs. <2 times/d in last 24 h | BMI trajectory in those normal weight at baseline | Difference in intercept from reference -20.20; 95% CI: 20.39 to 20.01 | Serious |
|  |  |  |  |  |  |  |  |  |  |  |
| 2–<5 y | | | | | | | | | | |
| Costa 2019 (22) | 4 y | 4 y | 315 | Two 24-h dietary recalls | Soft drink | %EI | Continuous | WC (cm); WHtR (cm); SSF (mm) | Change in WC: β = 0.01, 95% CI -0.22 to 0.19 P= 0.892; change in WHtR β =0.00, 95% CI -0.00 to 0.00; Change in SSF β =- 0.04, 95% CI 0.34 to 0.27 | Moderate |
| Newby 2004 (81) | 2.9 y | 8.4 mo | 1345 | 1-mo FFQ | Soda | oz/d | Continuous | Weight change (lb)/y | Fruit drinks: β = -0.03, SE = 0.02, P = 0.42; Soda β = -0.01, SE = 0.04, P = 0.81 | Moderate |
| Olafsdottir 2014 (82) | 2–<6 y; 6–<10 y | 2 y | 11038 | 28-d recall FFQ | SSB | Frequency/wk | Continuous | % increase in BMI; % increase in WHtR | 2-<6 y: % increase in BMI (OR = 1.01, 95%CI = 0.99, 1.03, P > 0.05) or waist to hip ratio (OR = 1.00, 95%CI = 0.98, 1.03, P >0.05); 6-<10 y: % increase in BMI (OR = 1.00, 95% CI = 0.99, 1.02, P > 0.05) or waist to hip ratio (OR = 1.01, 95% CI = 0.999, 1.03, P > 0.05) | Moderate |
| Sugimori 2004 (89) | 3 y | 3 y | 4176 boys; 3994 girls | Questionnaire | Juice | Not stated | Continuous | Proportion consuming juice drink by weight status change from 3 y to 6 y normal weight vs. obese status (normal/normal, normal to obese, obese to normal, obese/obese) | No significant differences in proportion consuming juice drinks, unadjusted analysis | Critical |
|  |  |  |  |  |  |  |  |  |  |  |
| 5– 10 y | | | | | | | | | | |
| Alviso-Orellana 2018 (48) | 8 y | 4 y | 1414 | 30-d recall | SSB | Frequency per 2 wk/wk/d | up to every 2 wk; 2-6 times/wk; daily or never | WC (cm); WHtR (cm); SSF (mm) | β = 1.43, 95% CI= -0.41, 3.27, P > 0.05 | Moderate |
| Olsen 2012 (83) | Boys 9.7 y; Girls 9.4 y | 6 y | 359 | 24-h recall interview, FFQ and a qualitative food record | Liquid sucrose | per 10g intake | Continuous | Change in WC (cm) | β = 0.220, SE = 0.138, P = 0.11 | Critical |
| Tam 2006 (90) | 7.7 y | 5.4 y | 281 | 3-d food record at baseline | Soft drinks/cordial | g/d | Continuous | BMI gains/losses | Comparison of acceptable BMI (Mean = 20, SD = 0–71 g/d), BMI gainers (Mean = 29, SD = 0-91 g/d), BMI losers (Mean = 6.5, SD = 0-170 g/d) from 7–13 y had significantly lower intakes of soft drink/cordial consumption vs. overweight/obese (Mean = 30, SD = 0-108 g/d) at both time points (Kruskall Wallis tests, P < 0.005) : Comparison of acceptable BMI (Mean = 14, SD = 0–48 g/d), BMI gainers (Mean = 8.6, SD = 0–59 g/d), BMI losers (Mean = 13, SD = 0-41.4 g/d) from 7–13 y had no significantly differences in juice/juice drink consumption (continuous variable) compared to those who were OW/OB at both time points (Mean = 14, SD = 0–44 g/day) (ANOVA, P > 0.05) | Critical |
| Traub 2018 (92) | 7.08 y | 1 y | 1250 | Questionnaire completed by parents | Soft drinks | Frequency/d; Frequency/wk | > 1 time/wk vs. <1 time/wk | Abdominal obesity (%) WHtR ≥ 0.5 | No significant difference in the odds of abdominal obesity by intake | Moderate |
| Zheng 2014 (97) | 9.6 y | 12 y | 171 | 24-h recall; face-to-face interview and qualitative food record | SSB | Servings (354 mL)/d | > 1 serving/d vs. ≤ 1 serving/d | Change in WC (cm); Change in SSF (mm) | Intake at 9 y and WC at 21 y P > 0.05. Change in intake from 9–15 y and change in WC from 15–21 y P > 0.05. No change vs. increased SSB intake 9–15 y WC (b = 2.72, P = 0.04), but SSF P > 0.05 | Moderate |
|  |  |  |  |  |  |  |  |  |  |  |
| **ASB** | | | | | | | | | | |
| 2–<5 y | | | | | | | | | | |
| Newby 2004 (81) | 2.9 y | 8.4 mo | 1345 | 1-mo FFQ | Diet soda | oz/d | Continuous | Weight change (lb)/y | β = 0.01, SE = 0.02, P = 0.92 | Moderate |
|  |  |  |  |  |  |  |  |  |  |  |
| **100% fruit juice** | | | | | | | | | | |
| 0–<2 y | | | | | | | | | | |
| Guerrero 2016 (65) | 9 mo | 63 mo | 15418 | Parent interview at 48, 60, and 72-mo | Juice | Intake vs. no intake in last 7 d | Any vs. none | BMI trajectory | Est = -0.101, SE = 0.053, P > 0.05 | Moderate |
|  |  |  |  |  |  |  |  |  |  |  |
| 2–<5 y | | | | | | | | | | |
| Faith 2006 (10) | 30.2 mo | 48 mo | 971 | Self administered questionnaire |  | Servings (184 mL)/d | Continuous | BAZ trajectory (slope) | β = 0.05, SE = 0.002, P < 0.01 | Serious |
| Newby 2004 (81) | 2.9 y | 8.4 mo | 1345 | 1-mo FFQ | Fruit juice only | oz/d | Continuous | Weight change (lb)/y | β = 0.01 SE = 0.01, P = 0.23 | Moderate |
| Shefferly 2016 (85) | 2 y | 2–3 y | 6250 | 7 d recall frequency | Fruit juice at 2-4 y; Fruit juice at 4-5 y | Servings (236.5 mL)/d | ≥ 1 serving/d vs. <1 serving/d | Weight-for-age | <1/ wk vs. ≥ 1 at 2–4 y (0.371 (0.032) vs. 0.432 (0.024), P = 0.055) or 4–5 y (0.042 (0.016) vs. 0.029 (0.012), P = 0.4553) | Moderate |
| Wan 2020 (94) | 3–6 y | 10 y | 100 | Multiple sets of 3-day diet records | 100% fruit juice | Cup equivalent/d | <0.5 cups; 0.5 ≤1.0 cups; ≥1.0 cups | Adiposity rebound | (No estimates) Longitudinal juice intake was not significantly associated with child height, weight, or BMI. | Serious |
| 5– 10 y | | | | | | | | | | |
| Arcan 2013 (111) | 5.8 y | 15 mo | 424 | Food consumption questionnaire | 100% fruit juice | Frequency/d | Continuous | Normal weight, overweight or obese (based on BMI) at follow up grouped by baseline weight status category | No significant association with 100% juice consumption. | Moderate |
|  |  |  |  |  |  |  |  |  |  |  |
| **Unhealthy foods^3^** | | | | | | | | | | |
| 0–<2 y | | | | | | | | | | |
| Garden 2011 (62) | 18 mo | 6.5 y | 362 | 3-d weighed food record | Extra foods (cookies, crackers, juice, cordial, fruit drinks and soft drinks, fats and oils, snack foods, sugar, confectionary, savory sauces, condiments, fried potatoes, ice-cream, and some miscellaneous foods); Dairy products (milk and milk products, including yoghurt, cheese, ice cream and custard) | g/d | Quintiles of intake as g/d | WC (cm) | Extra foods β = -0.31, 95%CI = -0.85, 0.23, P = 0.26 for trend; dairy products β = -0.45, 95%CI = -0.99, 0.08, P = 0.10 for trend | Moderate |
| Guerrero 2016 (65) | 9 mo | 63 mo | 15418 | Parent interview at 48, 60, and 72-mo | Fast food | Intake vs. no intake in last 7 d | Any vs. none | BMI trajectory in those normal weight at baseline | β = 0.103, SE = 0.035, P < 0.05 | Moderate |
| Jardi 2019 (28) | 0 mo | 30 mo | 81 | 24-h dietary recall at 12 mo | Free sugars (sweetened dairy desserts, sugary drinks, sweetened cereals, chocolate, sugar, and honey | %EI | Continuous | Excess vs. non-excess weight (present/absent) | OR: 1.130, 95% CI = 1.032, 1.238, P = 0.008 | No information |
| Moore 2019 (79) | 3–12 mo | 3 y | 666 | 3-mo FFQ | Snack food (biscuits, puffs, melts); Sweets (cookies, cakes, or candy) | Frequency/d | Consumed often vs. never | Weight-for-length z score | ANOVA sweets (F = 3.23, P = 0.03); sweets * time interaction (F = 2.44, P = 0.04) | Moderate |
| Thurber 2017 (91) | 0.5–2 y and 3–5 y; 0.5–2 y; 3–5 y |  | 907 | Caregiver report | High fat foods | Frequency/d | ≥ 2 times/d in last 24 h vs. <2 times/d in last 24-h | BMI trajectory in those normal weight at baseline | Difference in intercept from reference -0.15; 95% CI: -0.34, 0.04 | Serious |
|  |  |  |  |  |  |  |  |  |  |  |
| 2–<5 y | | | | | | | | | | |
| Costa 2019 (22) | 4 y | 4 y | 315 | 12-mo FFQ | Ultra-processed foods | %EI | Continuous | WC (cm); WHtR (cm); SSF (mm) | 6<10 y WC z score: boys (P = 0.05), girls (P = 0.05), SSF: boys (P = 0.05), girls (P = 0.02) | Moderate |
| Newby 2003 (80) | Boys 2.9 y; Girls 2.9 y | 6–12 mo | 1379 | Semi-quantitative FFQ | 'Fat foods' (includes ice-cream, mayonnaise, potato chips, cookies, cakes, pie, chocolate, hot dogs, bologna, butter, margarine, fried chicken, fried fish, sausage, bacon, donuts, sweet rolls, and French fries) | Frequency/d | Continuous | Weight change (kg)/y | β = 0.05, SE = 0.02, P = 0.03 | Serious |
| Russo 2018 (21) | Boys 4.2 and 7.4 y; Girls 4.2 and 7.4 y | 2 y | 6929 | FFQ via Children’s Eating Habits Questionnaire | Added sugars to milk and fruit | Daily/weekly/rarely | Daily (once or more times/d) vs. Rarely (never/less than once a wk); Weekly (<1/d) vs. Rarely (never/less than once a wk) | WC z score; SSF (mm) | 2<6 y: WC z score boys (P = 0.001), girls (P = 0.01); SSF boys (P = 0.05), girls (P = 0.02) | Moderate |
| Sugimori 2004 (89) | 3 y | 3 y | 3994 girls | Questionnaire | Instant noodles | Frequency/wk | Continuous | Normal weight at 3y and 6 y, obese/normal at 3/6 y; normal/obese at 3/6 y or obese/obese at 3/6 y | P > 0.05 for all comparisons (unadjusted) | Critical |
|  |  |  |  |  |  |  |  |  |  |  |
| 5– 10 y | | | | | | | | | | |
| Alviso-Orellana 2018 (48) | 8 y | 4 y | 1414 | 30-d recall | Snacks-salty and fatty foods (crisps, fried snacks) | Frequency/2 wk/wk/d | Up to every 2 wk; 2-6 times/wk; daily or never | WC (cm) | β = 0.85, 95% CI= -0.89, 2.6, P > 0.05 | Moderate |
| Olsen 2012 (83) | Boys 9.7 y; Girls 9.4 y (combined in analysis) | 6 y | 359 | 24-h recall interview, FFQ and a qualitative food record | Added sugar; Solid sucrose (added sugar/sucrose + liquid sucrose) | per 10g intake | Continuous | Change in WC (cm) | Added sugar: β = 0.148, SE = 0.083, P = 0.08; Solid sugar: β = 0.063, SE = 0.122, P = 0.6 | Critical |

^1^ASB, artificially-sweetened beverages; BAZ, BMI-for-age z score; DAT, dietary assessment tool; DXA, dual x-ray absorptiometry; FMI, fat mass index; FFQ, food-frequency questionnaire; NS, not stated; OW/OB, overweight including obesity; OB, obesity only; %EI, percentage of energy intake; RoB, risk of bias; SSB, sugar-sweetened beverages; SSF, sum of skinfolds; WC, waist circumference; WHtR, waist-to-height ratio.

^2^Minimum analytical sample size.

^3^Includes energy-dense, nutrient poor and ultra-processed foods

^5^1 fluid oz (US) = 29.6 mL

^6^1 cc = 1 ml

^7^1 cup equivalent = ~250 mL

**Supplemental Figure 1** Risk of bias assessment for non-randomized studies reporting the effect of unhealthy food and beverage consumption among children ≤10.9 y on growth and body composition outcomes using ROBINS-I


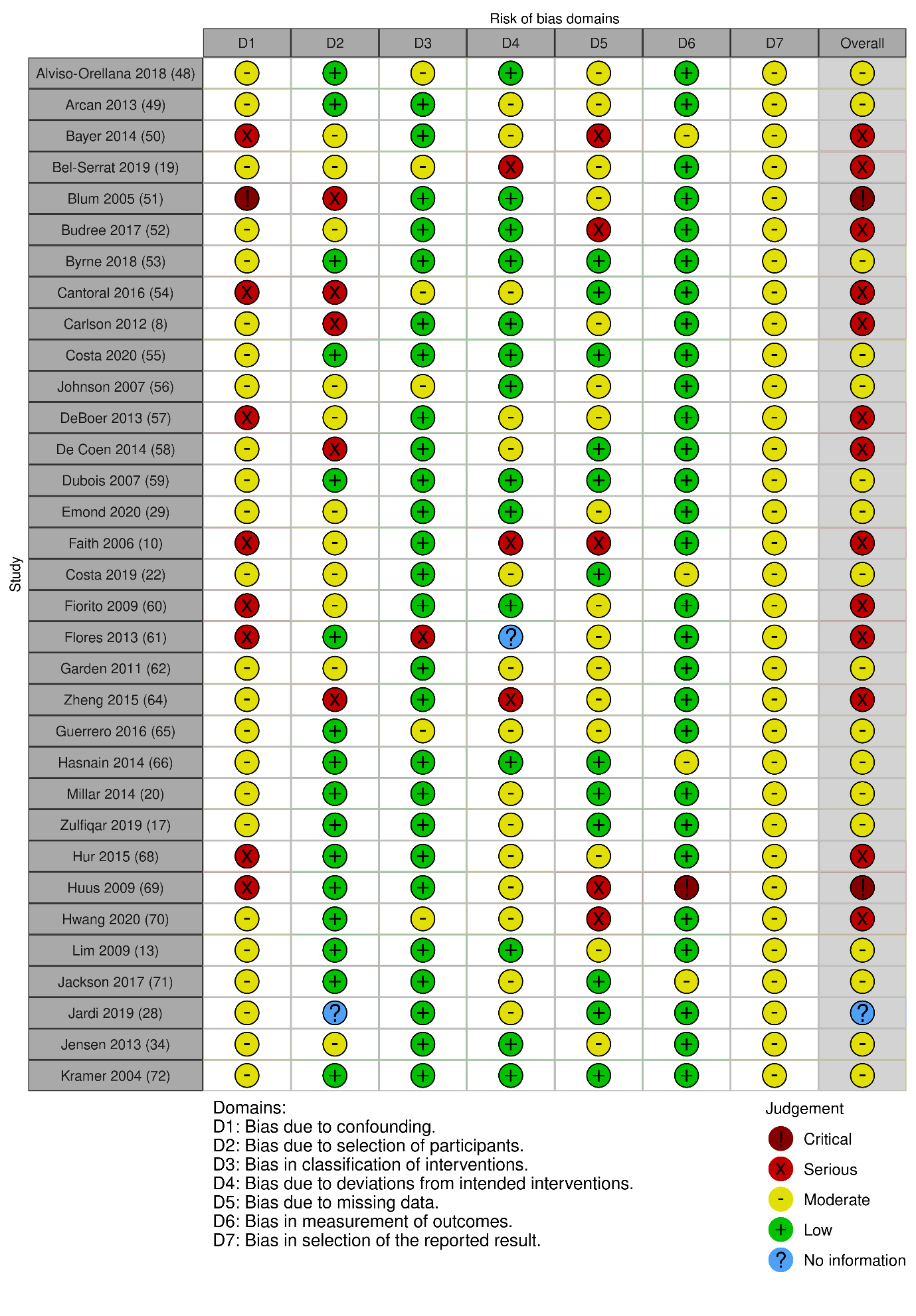


**Supplemental Figure 1** (continued) Risk of bias assessment for non-randomized studies reporting the effect of unhealthy food and beverage consumption among children ≤10.9 y on growth and body composition outcomes using ROBINS-I


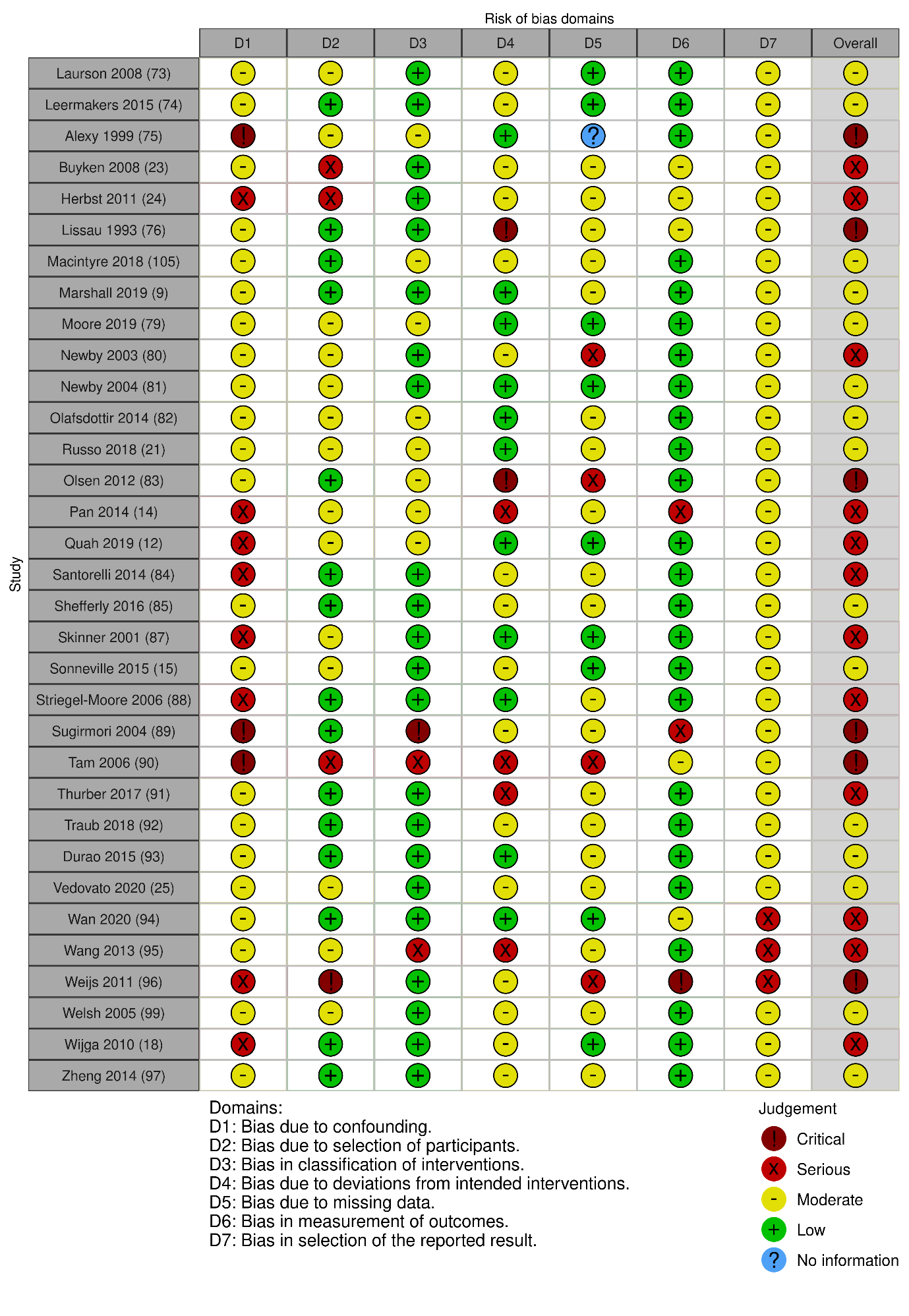


**Supplemental Figure 2** Risk of bias assessment for randomized controlled trials reporting the effect of unhealthy food and beverage consumption among children ≤10.9 y on growth, body composition and overweight/obesity outcomes using RoB V2.0


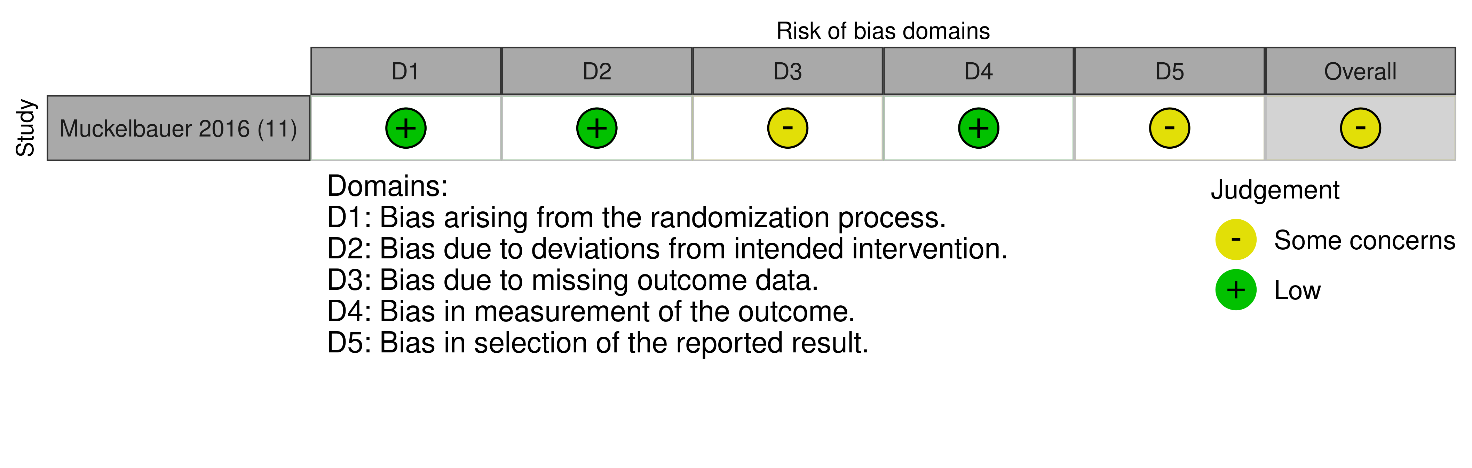


**Supplementary References**

1. Monteiro CA, Levy RB, Claro RM, de Castro IRR, Cannon G. A new classification of foods based on the extent and purpose of their processing. Cad Saude Publica. 2010 Nov;26(11):2039–49.

2. Monteiro CA, Cannon G, Moubarac JC, Levy RB, Louzada MLC, Jaime PC. The un Decade of Nutrition, the NOVA food classification and the trouble with ultra-processing. Public Health Nutr. 2018 Jan 21;21(1):5–17.

3. Monteiro CA, Levy RB, Claro RM, De Castro IRR, Cannon G. Increasing consumption of ultra-processed foods and likely impact on human health: Evidence from Brazil. Public Health Nutr. 2011 Dec 20;14(1):5–13.

4. Moubarac JC, Martins APB, Claro RM, Levy RB, Cannon G, Monteiro CA. Consumption of ultra-processed foods and likely impact on human health. Evidence from Canada. Public Health Nutr. 2013 Dec 21;16(12):2240–8.

5. Lawrence MA, Baker PI. Ultra-processed food and adverse health outcomes. BMJ. 2019 May 29;365:l2289.

6. World Health Organisation and the United Nations Children Fund. Indicators for assessing infant and young child feeding practices: definitions and measurement methods. Geneva: World Health Organization; 2021.

7. Swan GE, Powell NA, Knowles BL, Bush MT, Levy LB. A definition of free sugars for the UK. Public Health Nutr. 2018 Jun 28;21(9):1636–8.

8. Carlson JA, Crespo NC, Sallis JF, Patterson RE, Elder JP. Dietary-related and physical activity-related predictors of obesity in children: A 2-year prospective study. Child Obes. 2012;8(2):110–5.

9. Marshall TA, Curtis AM, Cavanaugh JE, Warren JJ, Levy SM. Sweetened beverage intakes are longitudinally associated with higher body mass index z scores in a birth cohort followed 17 years. J Acad Nutr Diet. 2019;119(3):425–34.

10. Faith MS, Dennison BA, Edmunds LS, Stratton HH. Fruit juice intake predicts increased adiposity gain in children from low-income families: weight status-by-environment interaction. Pediatrics. 2006 Nov 1;118(5):2066–75.

11. Muckelbauer R, Gortmaker SL, Libuda L, Kersting M, Clausen K, Adelberger B, et al. Changes in water and sugar-containing beverage consumption and body weight outcomes in children. Br J Nutr. 2016;115(11):2057–66.

12. Quah PL, Kleijweg J, Chang YY, Toh JY, Lim HX, Sugianto R, et al. Association of sugar-sweetened beverage intake at 18 months and 5 years of age with adiposity outcomes at 6 years of age: the Singapore GUSTO mother–offspring cohort. Br J Nutr. 2019 Dec 14;122(11):1303–12.

13. Lim S, Zoellner JM, Lee JM, Burt BA, Sandretto AM, Sohn W, et al. Obesity and sugar-sweetened beverages in African-American preschool children: A longitudinal study. Obesity. 2009;17(6):1262–8.

14. Pan L, Li R, Park S, Galuska DA, Sherry B, Freedman DS. A longitudinal analysis of sugar-sweetened beverage intake in infancy and obesity at 6 years. Pediatrics. 2014;134(Suppl 1 PG-S29-35):S29–35.

15. Sonneville KR, Long MW, Rifas-Shiman SL, Kleinman K, Gillman MW, Taveras EM. Juice and water intake in infancy and later bevereage intake and adiposity: Could juice be a gateway drink? Obes (Silver Spring). 2015;23(1):170–6.

16. Higgins JPT, Thomas J, Chandler; J, Cumpston; M, Li; T, Page; MJ, et al. Cochrane handbook for systematic reviews of interventions. Second. Hoboken, NJ: Wiley-Blackwell; 2019.

17. Zulfiqar T, Strazdins L, Dinh H, Banwell C, D’Este C, D’Este C, et al. Drivers of Overweight/Obesity in 4–11 Year Old Children of Australians and Immigrants; Evidence from Growing Up in Australia. J Immigr Minor Heal. 2019;21(4):737–50.

18. Wijga AH, Scholtens S, Bemelmans WJEE, Kerkhof M, Koppelman GH, Brunekreef B, et al. Diet, screen time, physical activity, and childhood overweight in the general population and in high risk subgroups: Prospective analyses in the PIAMA birth cohort. J Obes. 2010;2010(PG-).

19. Bel-Serrat S, Heinen MM, Mehegan J, O’Brien S, Eldin N, Murrin CM, et al. Predictors of weight status in school-aged children: a prospective cohort study. Eur J Clin Nutr. 2019;73(9):1299–306.

20. Millar L, Rowland B, Nichols M, Swinburn B, Bennett C, Skouteris H, et al. Relationship between raised BMI and sugar sweetened beverage and high fat food consumption among children. Obesity. 2014;22(5):96–103.

21. Russo M Dello, Ahrens W, De Henauw S, Eiben G, Hebestreit A, Kourides Y, et al. The impact of adding sugars to milk and fruit on adiposity and diet quality in children: A cross-sectional and longitudinal analysis of the identification and prevention of dietary-and lifestyle-induced health effects in children and infants (IDEFICS) stu. Nutrients. 2018;10(10).

22. Costa CS, Rauber F, Leffa PS, Sangalli CN, Campagnolo PDBB, Vitolo MR. Ultra-processed food consumption and its effects on anthropometric and glucose profile: A longitudinal study during childhood. Nutr Metab Cardiovasc Dis. 2019;29(2):177–84.

23. Buyken AE, Cheng G, Günther ALBB, Liese AD, Remer T, Karaolis-Danckert N. Relation of dietary glycemic index, glycemic load, added sugar intake, or fiber intake to the development of body composition between ages 2 and 7 y. Am J Clin Nutr. 2008;88(3):755–62.

24. Herbst A, Diethelm K, Cheng G, Icks UA, Buyken AE. Direction of associations between added sugar intake in early childhood and body mass index at age 7 years may depend on intake levels. J Nutr. 2011;141(7):1348–54.

25. Vedovato GM, Vilela S, Severo M, Rodrigues S, Lopes C, Oliveira A. Ultra-processed food consumption, appetitive traits and BMI in children: A prospective study. Br J Nutr. 2020;(7).

26. Schünemann HJ, Cuello C, Akl EA, Mustafa RA, Meerpohl JJ, Thayer K, et al. GRADE guidelines: 18. How ROBINS-I and other tools to assess risk of bias in nonrandomized studies should be used to rate the certainty of a body of evidence. J Clin Epidemiol. 2019 Jul;111:105–14.

27. Schünemann H, Brozek J, Guyatt G, Oxman A. Handbook for grading the quality of evidence and the strength of recommendations using the GRADE approach. Updated October 2013 [Internet]. 2013 [cited 2021 Jul 20]. Available from: https://gdt.gradepro.org/app/handbook/handbook.html

28. Jardí C, Aranda N, Bedmar C, Ribot B, Elias I, Aparicio E, et al. Consumption of free sugars and excess weight in infants. A longitudinal study. An Pediatría (English Ed. 2019;90(3):165–72.

29. Emond JA, Longacre MR, Titus LJ, Hendricks K, Drake KM, Carroll JE, et al. Fast food intake and excess weight gain over a 1-year period among preschool-age children. Pediatr Obes. 2020;15(4):1–9.

30. Sterne J, Hernán M, Reeves B, Savović J, Berkman N, Viswanathan M, et al. Risk Of Bias In Non-randomized Studies of Interventions (ROBINS-I): detailed guidance [Internet]. Vol. 355, Bmj. 2016 [cited 2021 May 29]. p. 1–53. Available from: http://www.riskofbias.info

31. Berkey CS, Rockett HRH, Field AE, Gillman MW, Colditz GA. Sugar-added beverages and adolescent weight change. Obes Res. 2004;12(5):778–88.

32. Bisset S, Gauvin L, Potvin L, Paradis G. Association of body mass index and dietary restraint with changes in eating behaviour throughout late childhood and early adolescence: a 5-year study. Public Heal Nutr. 2007;10(8 PG-780–9):780–9.

33. Field AE, Austin SB, Gillman MW, Rosner B, Rockett HR, Colditz GA. Snack food intake does not predict weight change among children and adolescents. Int J Obes. 2004;28(10):1210–6.

34. Jensen BW, Nichols M, Allender S, De Silva-Sanigorski A, Millar L, Kremer P, et al. Inconsistent associations between sweet drink intake and 2-year change in BMI among Victorian children and adolescents. Pediatr Obes. 2013;8(4):271–83.

35. Johnson BA, Kremer PJ, Swinburn BA, De Silva-Sanigorski AM. Multilevel analysis of the Be Active Eat Well intervention: Environmental and behavioural influences on reductions in child obesity risk. Int J Obes. 2012;36(7):901–7.

36. Lee EY, Kang B, Yang Y, Yang HK, Kim HS, Lim SY, et al. Study time after school and habitual eating are associated with risk for obesity among overweight Korean children: A prospective study. Obes Facts. 2018;11(1):46–55.

37. Mrdjenovic G, Levitsky DA. Nutritional and energetic consequences of sweetened drink consumption in 6- to 13-year-old children. J Pediatr. 2003;142(6):604–10.

38. Mundt CA, Baxter-Jones ADGG, Whiting SJ, Bailey DA, Faulkner RA, Mirwald RL. Relationships of activity and sugar drink intake on fat mass development in youths. Med Sci Sports Exerc. 2006;38(7):1245–54.

39. Nissinen K, Mikkilä V, Männistö S, Lahti-Koski M, Räsänen L, Viikari J, et al. Sweets and sugar-sweetened soft drink intake in childhood in relation to adult BMI and overweight. The Cardiovascular Risk in Young Finns Study. Public Health Nutr. 2009;12(11):2018–26.

40. Phillips SM, Bandini LG, Naumova EN, Cyr H, Colclough S, Dietz WH, et al. Energy-dense snack food intake in adolescence: Longitudinal relationship to weight and fatness. Obes Res. 2004;12(3):461–72.

41. Seferidi P, Millett C, Laverty AA. Sweetened beverage intake in association to energy and sugar consumption and cardiometabolic markers in children. Pediatr Obes. 2018;13(4):195–203.

42. Shroff MR, Perng W, Baylin A, Mora-Plazas M, Marin C, Villamor E. Adherence to a snacking dietary pattern and soda intake are related to the development of adiposity: A prospective study in school-age children. Public Health Nutr. 2014;17(7):1507–13.

43. Xue H, Wu Y, Wang X, Wang Y. Time trends in fast food consumption and its association with obesity among children in China. PLoS One. 2016;11(3):1–14.

44. Dong D, Bilger M, van Dam RM, Finkelstein EA. Consumption of specific foods and beverages and excess weight gain among children and adolescents. Health Aff. 2015;34(11):1940–8.

45. Alexy U, Libuda L, Mersmann S, Kersting M. Convenience foods in children’s diet and association with dietary quality and body weight status. Eur J Clin Nutr. 2011;65(2 PG-160–6):160–6.

46. Libuda L, Alexy U, Sichert-Hellert W, Stehle P, Karaolis-Danckert N, Buyken AE, et al. Pattern of beverage consumption and long-term association with body-weight status in German adolescents - Results from the DONALD study. Br J Nutr. 2008;99(6):1370–9.

47. World Bank. World Bank Country and Lending Groups [Internet]. 2021 [cited 2021 May 29]. Available from: https://datahelpdesk.worldbank.org/knowledgebase/articles/906519-world-bank-country-and-lending-groups

48. Alviso-Orellana C, Estrada-Tejada D, Carrillo-Larco RM, Bernabé-Ortiz A. Sweetened beverages, snacks and overweight: Findings from the Young Lives cohort study in Peru. Public Health Nutr. 2018;21(9):1627–33.

49. Arcan C, Hannan PJ, Fulkerson JA, Himes JH, Rock BH, Smyth M, et al. Associations of home food availability, dietary intake, screen time and physical activity with BMI in young American-Indian children. Public Health Nutr. 2013 Jan 29;16(1):146–55.

50. Bayer O, Nehring I, Bolte G, Von Kries R. Fruit and vegetable consumption and BMI change in primary school-age children: A cohort study. Eur J Clin Nutr. 2014;68(2):265–70.

51. Blum JW, Jacobsen DJ, Donnelly JE. Beverage Consumption Patterns in Elementary School Aged Children across a Two-Year Period. J Am Coll Nutr. 2005;24(2):93–8.

52. Budree S, Goddard E, Brittain K, Cader S, Myer L, Zar HJ. Infant feeding practices in a South African birth cohort—A longitudinal study. Matern Child Nutr. 2017;13(3):1–9.

53. Byrne R, Zhou Y, Perry R, Mauch C, Magarey A. Beverage intake of Australian children and relationship with intake of fruit, vegetables, milk and body weight at 2, 3.7 and 5 years of age. Nutr Diet. 2018;75(2):159–66.

54. Cantoral A, Téllez-Rojo MM, Ettinger AS, Hu H, Hernández-Ávila M, Peterson K. Early introduction and cumulative consumption of sugar-sweetened beverages during the pre-school period and risk of obesity at 8-14 years of age. Pediatr Obes. 2016;11(1):68–74.

55. Costa C dos S, Assunção MCF, Loret de Mola C, Cardoso J de S, Matijasevich A, Barros AJD, et al. Role of ultra-processed food in fat mass index between 6 and 11 years of age: a cohort study. Int J Epidemiol. 2020;1–10.

56. Johnson L, Mander AP, Jones LR, Emmett PM, Jebb SA. Is sugar-sweetened beverage consumption associated with increased fatness in children? Nutrition. 2007;23(7–8):557–63.

57. De Boer MD, Scharf RJ, Demmer RT. Sugar-sweetened beverages and weight gain in 2-to 5-year-old children. Pediatrics. 2013;132(3):413–20.

58. De Coen V, De Bourdeaudhuij I, Verbestel V, Maes L, Vereecken C. Risk factors for childhood overweight: A 30-month longitudinal study of 3- to 6-year-old children. Public Health Nutr. 2014;17(9):1993–2000.

59. Dubois L, Farmer A, Girard M, Peterson K. Regular sugar-sweetened beverage consumption between meals increases risk of overweight among preschool-aged children. J Am Diet Assoc. 2007;107(6):924–34.

60. Fiorito LM, Marini M, Francis LA, Smiciklas-Wright H, Birch LL. Beverage intake of girls at age 5 y predicts adiposity and weight status in childhood and adolescence. Am J Clin Nutr. 2009;90(4):935–42.

61. Flores G, Lin H. Factors predicting severe childhood obesity in kindergarteners. Int J Obes. 2013;37(1):31–9.

62. Garden FL, Marks GB, Almqvist C, Simpson JM, Webb KL. Infant and early childhood dietary predictors of overweight at age 8 years in the CAPS population. Eur J Clin Nutr. 2011;65(4):454–62.

63. Garden FL, Marks GB, Simpson JM, Webb KL. Body mass index (BMI) trajectories from birth to 11.5 years: relation to early life food intake. Nutrients. 2012;4(10 PG-1382–1398):1382–98.

64. Zheng M, Allman-Farinelli M, Heitmann BL, Toelle B, Marks G, Cowell C, et al. Liquid versus solid energy intake in relation to body composition among Australian children. J Hum Nutr Diet. 2015;28(s2):70–9.

65. Guerrero AD, Mao C, Fuller B, Bridges M, Franke T, Kuo AA. Racial and ethnic disparities in early childhood obesity: growth trajectories in body mass index. J Racial Ethn Heal Disparities. 2016 Mar 15;3(1):129–37.

66. Hasnain SR, Singer MR, Bradlee ML, Moore LL. Beverage intake in early childhood and change in body fat from preschool to adolescence. Child Obes. 2014 Feb;10(1):42–9.

67. Wheaton N, Millar L, Allender S, Nichols M. The stability of weight status through the early to middle childhood years in Australia: a longitudinal study. BMJ Open. 2015;5(4):e006963.

68. Hur YI, Park H, Kang JH, Lee HJHA, Song HJ, Lee HJHA, et al. Associations between sugar intake from different food sources and adiposity or cardio-metabolic risk in childhood and adolescence: The Korean child-adolescent cohort study. Nutrients. 2015;8(1).

69. Huus K, Brekke HK, Ludvigsson JJF, Ludvigsson JJF. Relationship of food frequencies as reported by parents to overweight and obesity at 5 years. Acta Paediatr Int J Paediatr. 2009;98(1):139–43.

70. Hwang IT, Ju Y-S, Lee HJ, Shim YS, Jeong HR, Kang MJ. Body mass index trajectories and adiposity rebound during the first 6 years in Korean children: Based on the National Health Information Database, 2008–2015. Chaput J-P, editor. PLoS One. 2020 Oct 30;15(10):e0232810.

71. Jackson SL, Cunningham SA. The stability of children’s weight status over time, and the role of television, physical activity, and diet. Prev Med (Baltim). 2017;100:229–34.

72. Kramer MS, Guo T, Platt RW, Vanilovich I, Sevkovskaya Z, Dzikovich I, et al. Feeding effects on growth during infancy. J Pediatr. 2004;145(5):600–5.

73. Laurson K, Eisenmann JC, Moore S. Lack of association between television viewing, soft drinks, physical activity and body mass index in children. Acta Paediatr Int J Paediatr. 2008;97(6):795–800.

74. Leermakers ETM, Felix JF, Erler NS, Ćerimagić A, Wijtzes AI, Hofman A, et al. Sugar-containing beverage intake in toddlers and body composition up to age 6 years: The Generation R Study. Eur J Clin Nutr. 2015 Mar 4;69(3):314–21.

75. Alexy U, Sichert-Hellert W, Kersting M, Manz F, Schöch G. Fruit Juice Consumption and the Prevalence of Obesity and Short Stature in German Preschool Children: Results of the DONALD Study. J Pediatr Gastroenterol Nutr. 1999;29(3):1999.

76. Lissau I, Breum L, Sorensen TIA. Maternal attitude to sweet eating habits and risk of overweight in offspring: A ten-year prospective population study. Int J Obes. 1993;17(3):125–9.

77. Macintyre AK, Marryat L, Chambers S. Exposure to liquid sweetness in early childhood: artificially-sweetened and sugar-sweetened beverage consumption at 4–5 years and risk of overweight and obesity at 7–8 years. Pediatr Obes. 2018;13(12):755–65.

78. Marshall TA, Curtis AM, Cavanaugh JE, Warren JJ, Levy SM. Higher longitudinal milk intakes are associated with increased height in a birth cohort followed for 17 years. J Nutr. 2018;148(7):1144–9.

79. Moore AM, Vadiveloo M, Tovar A, McCurdy K, Østbye T, Benjamin-Neelon SE. Associations of less healthy snack food consumption with infantweight-for-length Z-score trajectories: Findings from the nurture cohort study. Nutrients. 2019;11(11).

80. Newby PK, Peterson KE, Berkey CS, Leppert J, Willett WC, Colditz GA. Dietary composition and weight change among low-income preschool children. Arch Pediatr Adolesc Med. 2003 Aug 1;157(8):759–64.

81. Newby PK, Peterson KE, Berkey CS, Leppert J, Willett WC, Colditz GA. Beverage consumption is not associated with changes in weight and body mass index among low-income preschool children in North Dakota. J Am Diet Assoc. 2004;104(7):1086–94.

82. Olafsdottir S, Berg C, Eiben G, Lanfer A, Reisch L, Ahrens W, et al. Young children’s screen activities, sweet drink consumption and anthropometry: Results from a prospective European study. Eur J Clin Nutr. 2014;68(2):223–8.

83. Olsen NJ, Andersen LB, Wedderkopp N, Kristensen PL, Heitmann BL. Intake of liquid and solid sucrose in relation to changes in body fatness over 6 years among 8-to 10-year-old children: The European youth heart study. Obes Facts. 2012;5(4):506–12.

84. Santorelli G, Fairley L, Petherick ES, Cabieses B, Sahota P. Ethnic differences in infant feeding practices and their relationship with BMI at 3 years of age-results from the Born in Bradford birth cohort study. Br J Nutr. 2014 May 28;111(10):1891–7.

85. Shefferly A, Scharf RJ, Deboer MD. Longitudinal evaluation of 100% fruit juice consumption on BMI status in 2-5-year-old children. Pediatr Obes. 2016;11(3):221–7.

86. Skinner JD, Carruth BR, Moran J, Houck K, Coletta F. Fruit juice intake is not related to children’s growth. Pediatrics. 1999;103(1):58–64.

87. Skinner JD, Carruth BR. A longitudinal study of children’s juice intake and growth: the juice controversy revisited. J Am Die. 2001;101:432–7.

88. Striegel-Moore RH, Thompson D, Affenito SG, Franko DL, Obarzanek E, Barton BA, et al. Correlates of beverage intake in adolescent girls: The National Heart, Lung, and Blood Institute Growth and Health Study. J Pediatr. 2006 Feb;148(2):183–7.

89. Sugimori H, Yoshida K, Izuno T, Miyakawa M, Suka M, Sekine M, et al. Analysis of factors that influence body mass index from ages 3 to 6 years: A study based on the Toyama cohort study. Pediatr Int. 2004;46(3):302–10.

90. Tam CS, Garnett SP, Cowell CT, Campbell K, Cabrera G, Baur LA. Soft drink consumption and excess weight gain in Australian school students: Results from the Nepean study. Int J Obes. 2006;30(7):1091–3.

91. Thurber KA, Dobbins T, Neeman T, Banwell C, Banks E. Body mass index trajectories of Indigenous Australian children and relation to screen time, diet, and demographic factors. Obesity. 2017;25(4):747–56.

92. Traub M, Lauer R, Kesztyüs T, Wartha O, Steinacker JM, Kesztyüs D, et al. Skipping breakfast, overconsumption of soft drinks and screen media: Longitudinal analysis of the combined influence on weight development in primary schoolchildren. BMC Public Health. 2018;18(1):1–10.

93. Durão C, Severo M, Oliveira A, Moreira P, Guerra A, Barros H, et al. Evaluating the effect of energy-dense foods consumption on preschool children’s body mass index: a prospective analysis from 2 to 4 years of age. Eur J Nutr. 2015;54(5):835–43.

94. Wan L, Jakkilinki PD, Singer MR, Bradlee ML, LL Moore LL, LL ML. A longitudinal study of fruit juice consumption during preschool years and subsequent diet quality and BMI. BMC Nutr. 2020;6–25.

95. Wang N, Huang J, Li K, Zhao Y, Wen J, Ye Y, et al. Prevalence and risk factors of overweight and obesity among infants in Chongqing urban area. Chin J Contemp Pediatr. 2013;15(3).

96. Weijs PJMM, Kool LM, Van Baar NM, Van Der Zee SC. High beverage sugar as well as high animal protein intake at infancy may increase overweight risk at 8 years: A prospective longitudinal pilot study. Nutr J. 2011;10(1):1–8.

97. Zheng M, Rangan A, Olsen NJ, Bo Andersen L, Wedderkopp N, Kristensen P, et al. Sugar-sweetened beverages consumption in relation to changes in body fatness over 6 and 12 years among 9-year-old children: The European Youth Heart Study. Eur J Clin Nutr. 2014;68(1):77–83.

98. Jensen BW, Nielsen BM, Husby I, Bugge A, El-Naaman B, Andersen LB, et al. Association between sweet drink intake and adiposity in Danish children participating in a long-term intervention study. Pediatr Obes. 2013;8(4):259–70.

99. Welsh JA, Cogswell ME, Rogers S, Rockett H, Mei Z, Grummer-Strawn LM. Overweight among low-income preschool children associated with the consumption of sweet drinks: Missouri, 1999-2002. Pediatrics. 2005;115(2):1999–2002.

100. World Health Organization. WHO Child Growth Standards: length/height-for-age, weight-for-age, weight-for-length, weight-for-height and body mass index-for-age: methods and development. Geneva; 2006.

101. Kuczmarski R, Ogden C, Guo S, Grummer-Strawn LM, Flegal KM, Mei Z, et al. 2000 CDC growth charts for the United States: Methods and development. Natl Cent Heal Stat Vital Heal Stat. 2002;(Series 11, Number 246).

102. Cole TJ, Bellizzi MC, Flegal KM, Dietz WH. Establishing a standard definition for child overweight and obesity worldwide: International survey. Br Med J. 2000 May 6;320(7244):1240–3.

103. Ruekit S, Wangchuk S, Dorji T, Tshering KP, Pootong P, Nobthai P, et al. Molecular characterization and PCR-based replicon typing of multidrug resistant Shigella sonnei isolates from an outbreak in Thimphu, Bhutan. BMC Res Notes. 2014 Feb 20;7:95.

104. Xu F, Wang X, Ware RS, Tse LA, Wang Z, Hong X, et al. A school-based comprehensive lifestyle intervention among Chinese kids against Obesity (CLICK-Obesity) in Nanjing City, China: The baseline data. Asia Pac J Clin Nutr. 2014;23(1):48–54.

105. Macintyre AK, Marryat L, Chambers S. Exposure to liquid sweetness in early childhood: artificially-sweetened and sugar-sweetened beverage consumption at 4–5 years and risk of overweight and obesity at 7–8 years. Pediatr Obes. 2018;13(12):755–65.

106. Cole TJ, Freeman J V, Preece MA. British 1990 growth reference centiles for weight, height, body mass index and head circumference fitted by maximum penalized likelihood. Stat Med. 1998 Feb 28;17(4):407–29.

107. Cole TJ, Lobstein T. Extended international (IOTF) body mass index cut-offs for thinness, overweight and obesity. Pediatr Obes. 2012 Aug;7(4):284–94.

108. Cole TJ, Green PJ. Smoothing reference centile curves: the LMS method and penalized likelihood. Stat Med. 1992;11(10):1305–19.

109. Cole TJ, Freeman J V., Preece M a. British 1990 growth reference centiles for weight, height, body mass index and head circumference fitted by maximum penalized likelihood. Stat Med. 1998;17(4):407–29.

110. Skinner JD, Carruth BR. A longitudinal study of children’s juice intake and growth: the juice controversy revisited. J Am Die. 2001;101:432–7.

111. Story M, Hannan PJ, Fulkerson JA, Rock BH, Smyth M, Arcan C, et al. Bright start: Description and main outcomes from a group-randomized obesity prevention trial in american Indian children. Obesity. 2012;20(11):2241–9.
